# CD4-directed nanoblades enable selective genome editing in CD4^+^ cells and HIV suppression in vitro and in vivo

**DOI:** 10.1038/s44321-026-00481-x

**Published:** 2026-07-06

**Authors:** Jolien Van Cleemput, Maaike De Cock, Rianne Verbeek, Elianne Burg, Bram Van den Eeckhout, Wojciech Witkowski, Willem van Snippenberg, Zoe Stylianidou, Emily Brugger Galetic, Mareva Delporte, Ken Bracke, Sarah Gerlo, Linos Vandekerckhove

**Affiliations:** 1 HIV Cure Research Center, Department of Internal Medicine and Pediatrics, Faculty of Medicine and Health Sciences, Ghent University, Ghent, Belgium; 2 Laboratory of Virology, Department of Translational Physiology, Infectiology, and Public Health, Faculty of Veterinary Medicine, Ghent University, Merelbeke, Belgium; 3 Biopharmaceutical Technology Unit, Department of Pharmaceutics, Faculty of Pharmaceutics, Ghent University, Ghent, Belgium; 4 Laboratory for Translational Research of Obstructive Pulmonary Disease, Department of Respiratory Medicine, Ghent University Hospital and Ghent University, Ghent, Belgium; 5 Department of Biomolecular Medicine, Faculty of Medicine and Health Sciences, Ghent University, Ghent, Belgium

**Keywords:** Genetics, Gene Therapy & Genetic Disease, Immunology, Microbiology, Virology & Host Pathogen Interaction

## Abstract

Current antiretroviral therapies suppress HIV replication but fail to eliminate integrated proviral DNA in long-lived CD4⁺ cells, precluding a cure. CRISPR-Cas9 offers potential for HIV eradication but efficient and cell-specific delivery into HIV target cells remains a major hurdle. We developed CD4-directed Nanoblades (CD4-NBs), murine leukemia virus-like particles pseudotyped with anti-CD4 nanobodies and a fusogenic glycoprotein VSV Gmut, to selectively deliver Cas9-gRNA ribonucleoproteins into CD4⁺ cells. CD4-NBs selectively delivered cargo to CD4⁺ cells in vitro and in vivo, achieving efficient gene disruption in primary CD4^+^ cells. Dual-guide CD4-NBs targeting conserved HIV *tat*/*rev*/*env* regions disrupted proviral DNA, suppressing HIV infection in CD4^+^ cells. In HIV-infected, ART-pretreated humanized mice, CD4-NBs significantly reduced plasma viremia. While full tissue reservoir clearance was not achieved, repeated dosing did reduce viral RNA and proviral DNA in bone marrow and lungs, respectively. As such, this proof-of-concept study supports the promise of CD4-NBs as a minimally invasive, CD4⁺ cell-targeted gene editing strategy for HIV therapy.

## Synopsis

Virus-like particles engineered with anti-CD4 nanobodies (i.e., CD4-directed nanoblades) present a promising HIV cure strategy as they achieve CD4^+^ cell-specific CRISPR-Cas9 delivery, efficient gene editing, HIV proviral DNA disruption and reduced viremia in ART-pretreated humanized mice.

- CD4-NBs were engineered by pseudotyping murine leukemia virus-like particles with anti-CD4 nanobodies and a fusogenic glycoprotein to enable targeted Cas9-gRNA ribonucleoprotein delivery.
- Selective cargo delivery was achieved in vitro and in vivo, with efficient gene disruption observed specifically in (primary) CD4⁺ cells.
- HIV proviral DNA was disrupted using dual-guide CD4-NBs targeting conserved HIV *tat*/*rev*/*env* regions, resulting in suppressed HIV infection in CD4⁺ cells.
- In HIV-infected, ART-pretreated humanized mice, viremia was significantly reduced following CD4-NB administration.
- Repeated CD4-NB dosing was associated with reductions in viral RNA and proviral DNA in bone marrow and lungs, respectively, though full tissue reservoir clearance was not achieved.

## Abstract (web summary)

Virus-like particles engineered with anti-CD4 nanobodies (i.e., CD4-directed nanoblades) present a promising HIV cure strategy as they achieve CD4^+^ cell-specific CRISPR-Cas9 delivery, efficient gene editing, HIV proviral DNA disruption and reduced viremia in ART-pretreated humanized mice.

## Graphical abstract

**Figure:**
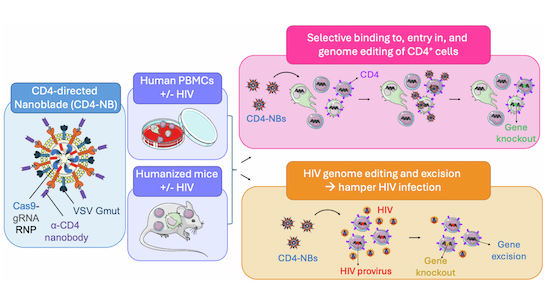

**Figure:**
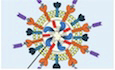

The paper explained **Problem**

HIV hides as a permanent reservoir inside the genome of certain immune cells called CD4⁺ cells. Current HIV medicines can stop the virus from replicating, but cannot remove these hidden copies, meaning people must stay on treatment for life. New genome-editing tools like CRISPR-Cas9 could cut out the virus from these cells, but getting the CRISPR toolbox safely and specifically into the right cells is very difficult.

**Results**

We created small delivery particles called CD4-directed nanoblades that can carry the CRISPR-Cas9 system directly into CD4⁺ cells. More specifically, CD4-directed nanoblades (i) delivered CRISPR-Cas9 efficiently and specifically to the right cells, enabling genome engineering in these cells, and (ii) excised essential parts of the viral genome, thereby reducing the amount of virus in cells in vitro and in the blood of HIV-infected mice.

**Impact**

This approach could bring us closer to a lasting cure, where HIV can be removed from the body. This holds more potential over current lifelong treatment.

## Introduction

The prospect of a functional or sterilizing cure for HIV has been significantly advanced by recent developments in gene-editing technologies, particularly CRISPR-Cas9. This system enables precise genome modifications and has demonstrated the potential to mutate and even excise integrated HIV proviral DNA from host genomes, plausibly offering a permanent genetic solution to HIV infection. However, despite encouraging proof-of-concept studies, a major obstacle to clinical translation remains the efficient and selective delivery of Cas9-gRNA ribonucleoproteins (RNPs) to HIV-infected cells in vivo (Das et al, 2019; Dash et al, 2019, 2023). CD4^+^ T cells, the primary target cells of HIV, pose unique challenges to genome-editing delivery. Their small size, high nucleus-to-cytoplasm ratio, nonphagocytic nature, and low endocytic activity severely limit uptake of gene-editing complexes (Cevaal et al, 2021). Over the recent years, a lot of progress has been made in optimizing delivery systems (e.g., Cas9 virus-like particles [VLPs], lentiviral vectors, adeno-associated viral vectors [AAVs] and lipid nanoparticles [LNPs]) for T cell editing ex vivo, especially in regard to immunotherapies (Herskovitz et al, 2021; Tombácz et al, 2021a; Banskota et al, 2022; Hamilton et al, 2021; Foss et al, 2022; Mangeot et al, 2019; Wang et al, 2020; Demircan et al, 2024). Still, these strategies have shown limited efficacy in vivo, as classical delivery vehicles are often sequestered by phagocytes, cleared by the liver and kidneys, or fail to reach HIV tissue reservoirs. As a countermeasure, nanomedicines have been decorated with targeting molecules (e.g., antibodies) against general T cell markers (e.g., CD3, CD8, CD4, and CD5), which improves their efficacy through lowering sequestration by bystander cells and reducing off-target effects and toxicity (Cevaal et al, 2021; Tombácz et al, 2021a; Schmid et al, 2017; Hamann et al, 2021; Xu et al, 2025). The group of Jennifer Doudna showed that displaying HIV envelope glycoprotein on Cas9-HIV VLPs directs genome editing exclusively to CD4^+^ T cells within a mixed-cell population (Hamilton et al, 2021). However, given the presence of preexisting immunity against HIV structural proteins in people living with HIV (PLWH), these nanoparticles would rapidly be neutralized or evoke immune reactions. Further, lentiviruses or AAVs pseudotyped with anti-CD4 or anti-CD8 designed ankyrin repeat proteins (DARPins) were able to transduce up to 2% of human CD4 or CD8 T cells in a humanized mouse model, respectively (Demircan et al, 2024; Zhou et al, 2015). However, viral transduction results in poor to moderate in vivo transduction efficacy, suffers from toxicity issues, limited cargo capacity, gene persistence, potential oncogenicity, and immunogenicity. Similarly, displaying anti-CD4 antibody fragments on Cas9-VLPs or lipid nanoparticles (LNPs) can target cargo delivery to CD4^+^ cells (Tombácz et al, 2021a; Hamilton et al, 2024). Still, receptor targeting via antibody binding in vivo can induce receptor-mediated signaling, potentially triggering unintended cellular responses. In contrast, nanobodies, the VHH domain of “heavy-chain-only” immunoglobulin molecules in *Camelidae*, are smaller, less immunogenic and easier to manipulate compared to antibody fragments (Muyldermans, 2013). In addition, the convex binding surface of nanobodies is better suited to interact with concave surfaces on antigens, in contrast to the concave binding surface of DARPins. In order to overcome the above-mentioned barriers, we sought to decorate a transient RNP delivery system, Cas9-VLPs, with anti-CD4 nanobodies and fusogenic glycoproteins to obtain CD4-directed nanoblades (CD4-NBs). By equipping these CD4-NBs with gRNAs targeting multiple essential HIV genes, we aimed to generate a specific anti-HIV therapeutic strategy.

## Results

### Generation and characterization of CD4-directed nanoblades

**Figure 1:**
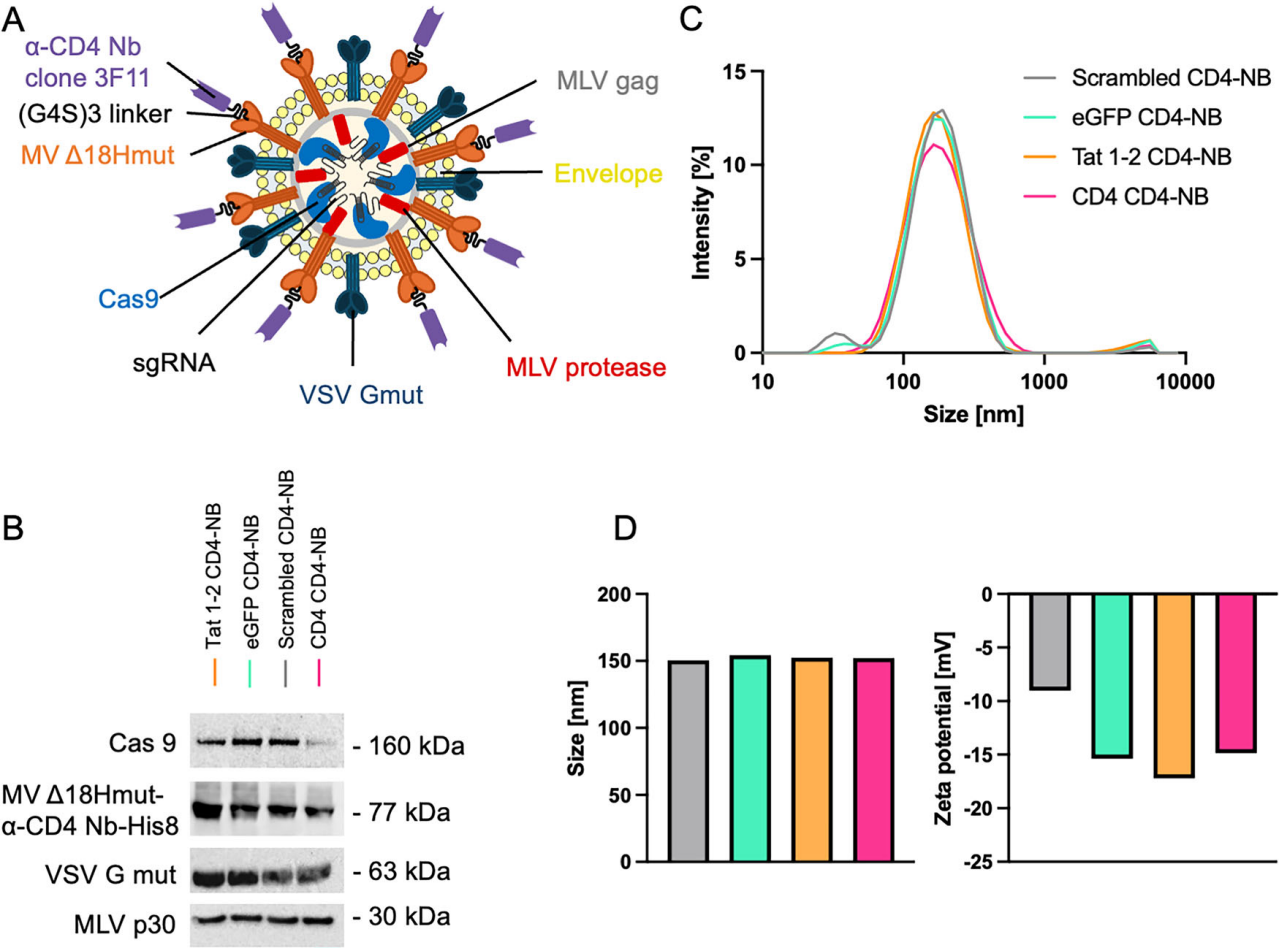
Characterization of CD4-directed nanoblades. (**A**) Schematic illustration of the CD4-NB structure. Abbreviations: α-CD4 Nb: anti-CD4 nanobody; MV Δ18Hmut: measles virus mutated hemagglutinin protein; sgRNA: single gRNA; VSV Gmut: mutated vesicular stomatitis virus glycoprotein; MLV: murine leukemia virus; gag: group-specific antigen. (**B**) Western blot analysis of exemplary CD4-NBs with four different gRNA compositions (scrambled gRNA, anti-eGFP gRNA, anti-CD4 gRNA, and anti-tat1 and anti-tat2 gRNAs). Full blots can be found in Source Data. (**C**,** D**) Physicochemical characteristics of exemplary CD4-NBs as measured by dynamic light scattering (DLS). Bar charts show the mean of three technical replicates. Source data are available online for this figure.

To obtain specific RNP delivery in CD4^+^ cells, the surface of the original nanoblades, i.e., Cas9 MLV-VLPs (Mangeot et al, 2019), was decorated with modified viral glycoproteins to obtain CD4-directed nanoblades (CD4-NBs) (Fig. 1A). More precisely, we fused the His-tagged anti-CD4 nanobody clone 3F11-coding sequence C-terminally to that of truncated mutated measles virus (MV) hemagglutinin (Δ18Hmut) via a long flexible (G4S)3 linker (Kneissl et al, 2012; Roobrouck et al, 2015). MV Hmut has four mutations in the receptor recognition regions to prevent MV receptor binding (Kneissl et al, 2012). Further, we codisplayed mutant VSV G (K47Q, R354A; VSV Gmut) on the VLP surface (Yu et al, 2022). VSV Gmut is defective in native receptor binding but retains its fusion ability. As such, specific binding of CD4-NBs to CD4 cells is enabled through MV Δ18Hmut-3F11 while cargo release is mediated through the fusogenic activity of VSV Gmut. In addition, the Gag-NLS-Cas9 production plasmid was exchanged for a third-generation version (v3) where a 3x nuclear export signal (NES) motif was installed upstream of the MLV protease cleavable linker to encourage cytoplasmic localization of Gag-3xNES-Cas9 in producer cells but nuclear localization of RNPs in transduced cells. As such, more RNP cargo can be incorporated in VLPs, enhancing gene-editing efficacy (Banskota et al, 2022). As detected by Western blot, all four CD4-NBs used throughout the study, each differing in gRNA composition, contain the key building blocks, including MV Δ18Hmut-3F11 (his-tag), VSV Gmut, Cas9, and MLV p30 capsid proteins (Fig. 1B). The amount of Cas9 was quantified using ELISA to standardize Cas9 doses per experiment. The unimodal distribution and narrow polydispersity index or PDI (0.25 ± 0.01) confirm uniform VLP assembly (Fig. 1C,D). The average diameter (152.9 ± 4.1 nm) is comparable to previously described VLP-based delivery systems, suggesting proper self-assembly of the Cas9-VLPs (Bezbaruah et al, 2022). The moderately negative zeta potential (−14.1 ± 3.4 mV) implies good colloidal stability while still allowing interaction with cellular membranes.

### CD4-directed nanoblades specifically deliver cargo to CD4^+^ cells in vitro and in vivo

**Figure 2:**
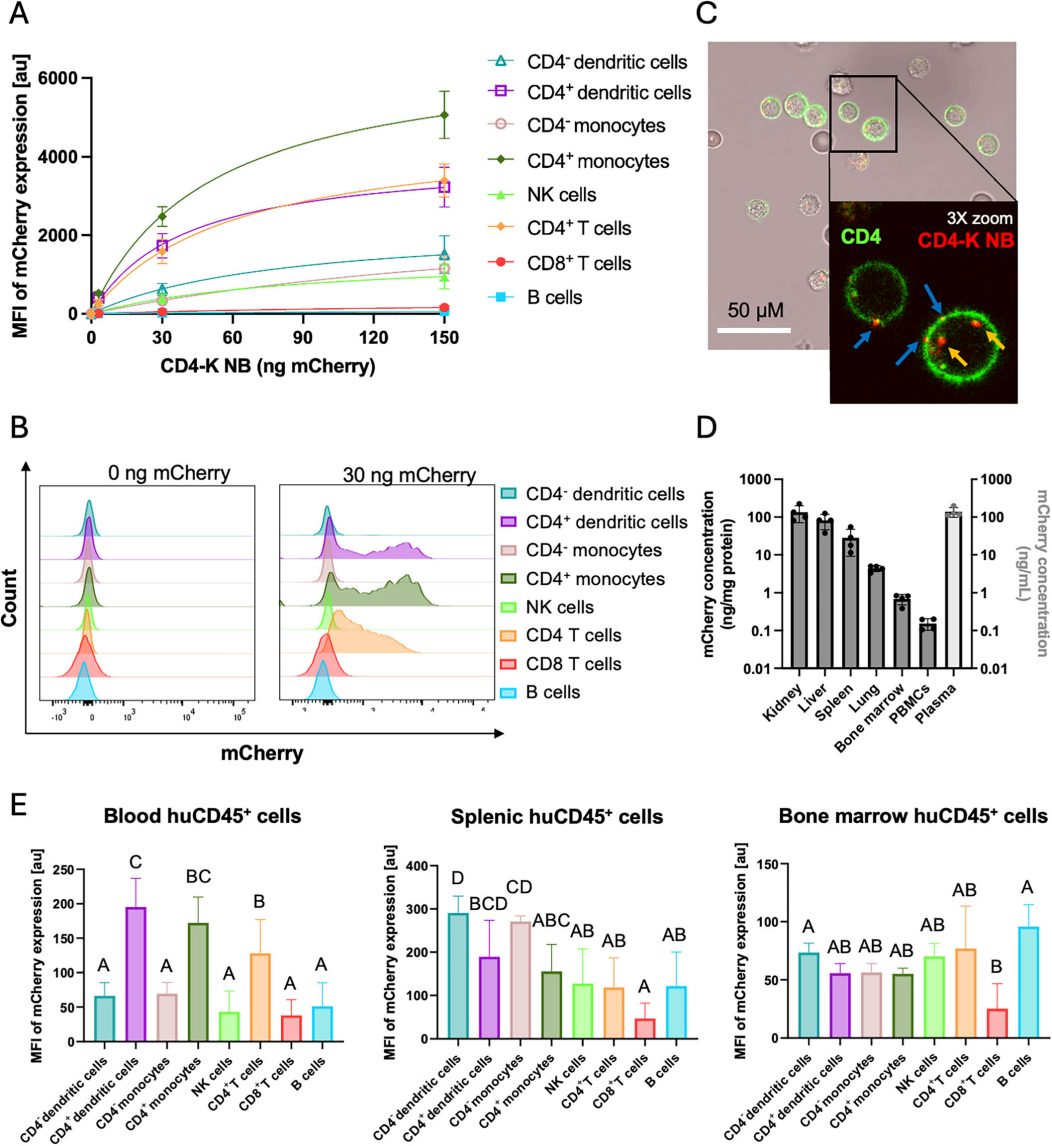
CD4-directed kirsch nanoblades selectively bind to and enter primary CD4^+^ cells in vitro and in vivo. (**A**) Dose-response curve of mCherry signal after in vitro CD4-directed kirsch nanoblade (CD4 K-NB) delivery, expressed as mCherry mean fluorescence intensity (MFI), across different PBMC subsets after 1 h incubation (mean ± SEM, *n* = 3 technical replicates of three different donors). (**B**) Representative flow cytometry histograms of PBMC subsets (donor 1) after 1 h PBMC incubation with 0 ng (left) or 30 ng (right) mCherry-loaded CD4 K-NBs. (**C**) Representative confocal image of CD4 K-NB attachment to (blue arrows) and entry into (orange arrows) primary CD4^+^ cells. (**D**) Tissue and plasma distribution of CD4 K-NBs, expressed as mCherry signal, 3 h post injection in humanized mice. Dark gray bars: tissues; light gray bar: plasma; mean ± SD; *n* = 4 mice. (**E**) Binding of CD4 K-NBs to huCD45^+^ immune cell subsets, expressed as mCherry mean fluorescence intensity (MFI), in blood, spleen, and bone marrow of humanized mice 3 h post injection. Analysis was done with one-way ANOVA followed by a Tukey post hoc test (Mean ± SD, different letters indicate significant [*p* < 0.05, exact *p* values are reported in Appendix Tables S1–3] differences, *n* = 5 mice (spleen and bone marrow) or 7 mice (blood). Source data are available online for this figure.

**Figure EV1:**
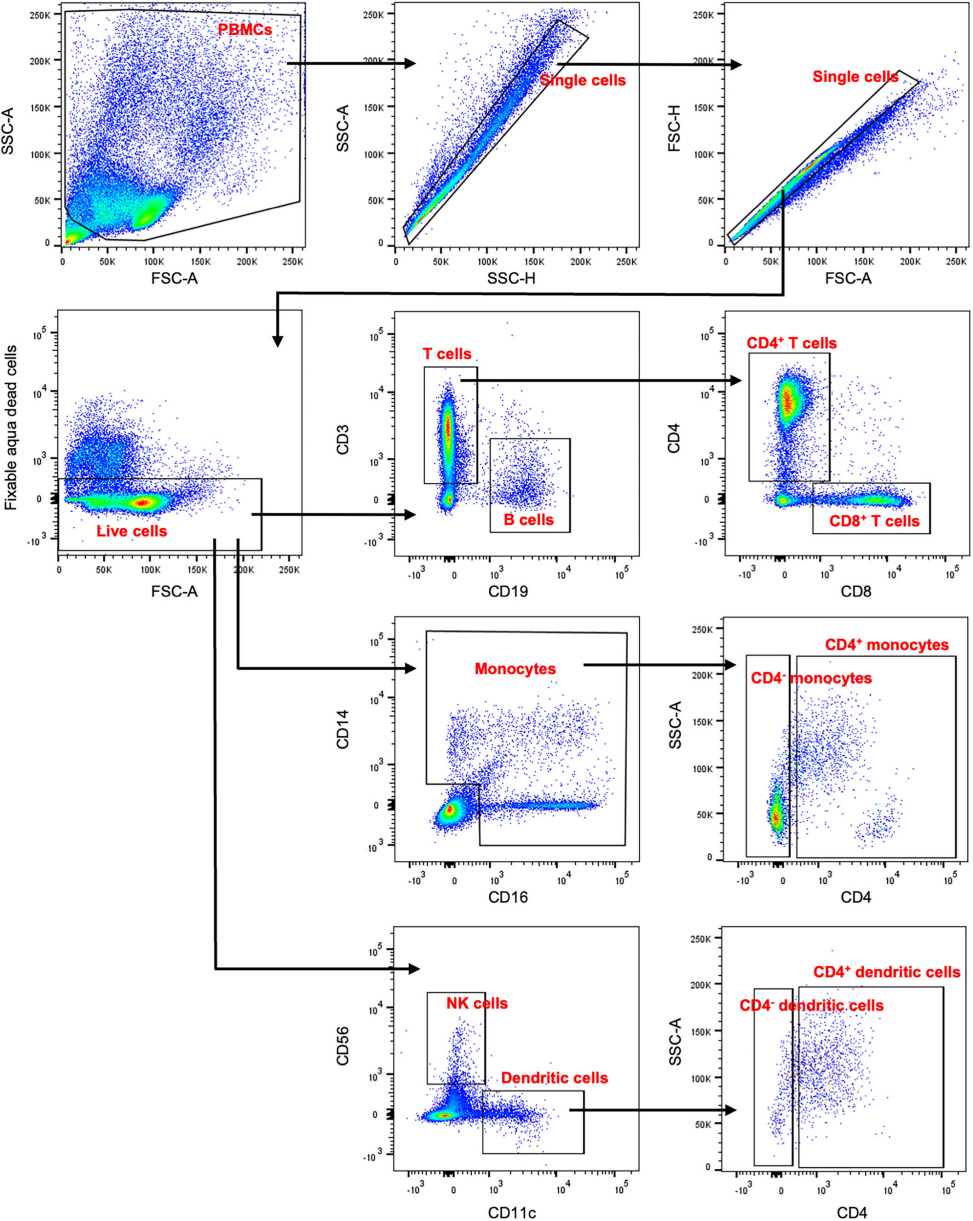
Sequential gating to identify specific human PBMC subsets incubated with CD4-directed kirsch nanoblades (CD4 K-NBs). The gating strategy of a representative sample (0 ng mCherry donor 1) is shown by arrows.

To track cell binding and entry of CD4-NBs, we first produced CD4-directed kirsch nanoblades (CD4 K-NBs), which contain mCherry in place of Cas9. Similarly as for CD4-NBs, dosage was standardized using ELISA quantifying mCherry protein. One hour post addition to primary peripheral blood mononuclear cells (PBMCs), dose-responsive binding to CD4^+^ T cells, CD4^+^ CD11c^+^ dendritic cells and CD4^+^ monocytes was observed using flow cytometry (Figs. 2A,B and EV1). The mCherry mean fluorescence intensity (MFI) of all CD4^+^ subsets was on average 6.7 ± 3.8-fold higher than CD4^-^ cell subsets across all mCherry concentrations and donors. Selective binding to and entry into CD4^+^ cells was also confirmed using confocal microscopy (Fig. 2C).

**Figure EV2:**
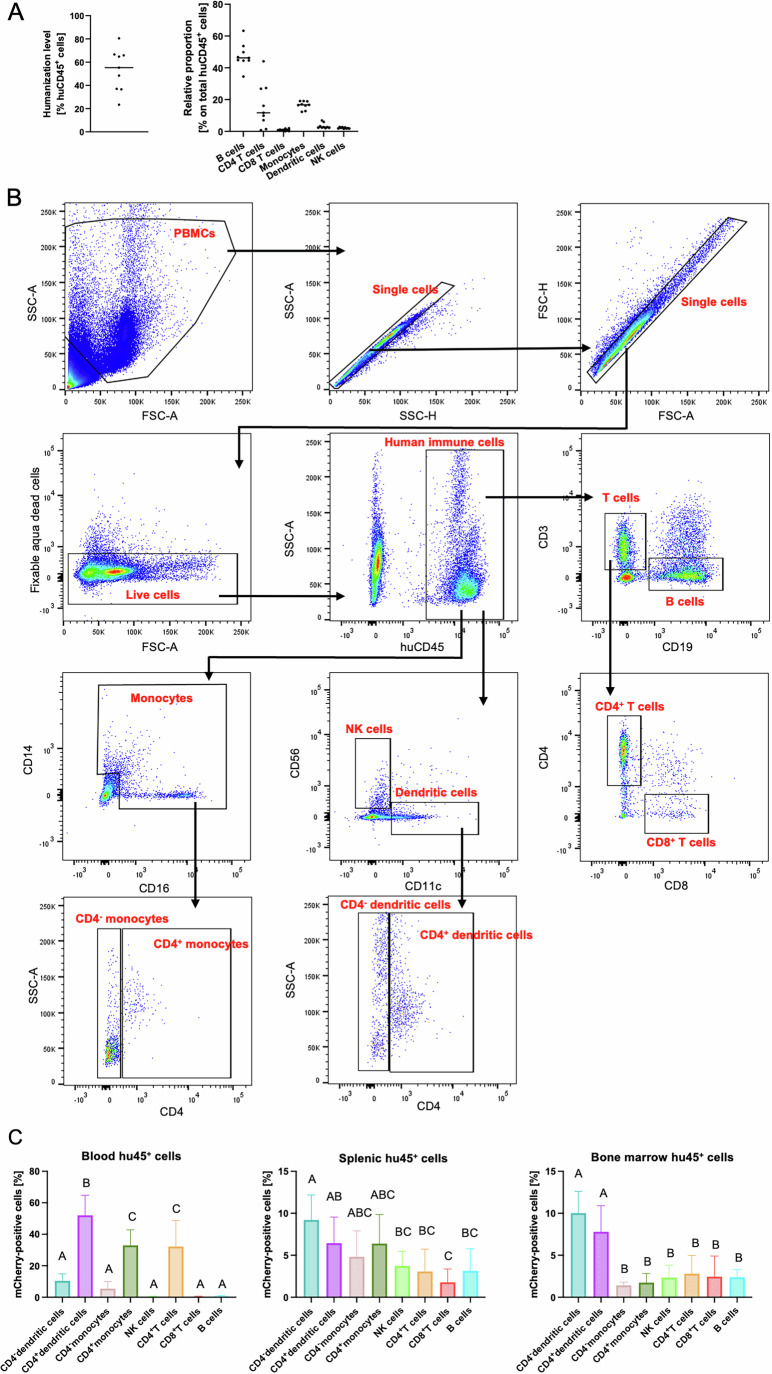
Characterization of humanized mice and gating strategy to determine in vivo binding of CD4 K-NBs to human immune cells. (**A**) Left panel: dot blot with grand mean of humanization levels of mice (*n* = 9) prior to injection with CD4 K-NBs shown as % of huCD45^+^ cells over total muCD45^+^ and huCD45^+^ cells. Right panel: Relative proportion of specific human immune cell subsets in these humanized mice represented as % of specific cell types over total huCD45^+^ cells. (**B**) Sequential gating to identify specific human immune cell subsets in humanized mice injected with CD4 K-NBs. The gating strategy of a representative sample (0 ng mCherry) is shown by arrows. (**C**) Binding of CD4 K-NBs to huCD45^+^ immune cell subsets, expressed as % of mCherry-positive cells in blood, spleen, and bone marrow of humanized mice 3 h post injection. Analysis was done with one-way ANOVA followed by a Tukey post hoc test (mean + SD, different letters indicate significant [*p* < 0.05] differences, *n* = 7 mice for blood and 5 mice for spleen and bone marrow).

Next, we verified the in vivo organ distribution and CD4⁺ cell targeting by CD4 K-NBs. Since nanobody 3F11 does not cross-react with murine CD4, CD4 K-NBs (2 µg mCherry) were injected intravenously into 11-week-old humanized NSG-SGM3 mice with humanization levels >20% (Fig. EV2A). In contrast to regular NSG mice, NSG-SGM3 mice enable stronger human immune cell engraftment, particularly in the lymphoid and myeloid compartments, which include CD4^+^ cells (Billerbeck et al, 2011). Three hours post injection, plasma mCherry levels reached 143.37 ± 39.47 ng/mL. As shown in Fig. 2D, CD4 K-NBs preferentially accumulated in the kidney (153.57 ± 52.94 ng mCherry/mg protein), followed by the liver (96.96 ± 20.18 ng/mg), and spleen (33.91 ± 15.51 ng/mg). Minimal accumulation was observed in the lungs (4.21 ± 0.70 ng/mg), PBMC pellets (0.14 ± 0.04 ng/ mg) and bone marrow cell pellets (0.80 ± 0.05 ng/mg). Despite low overall mCherry concentration in PBMCs, significantly (*p* < 0.05) higher levels of mCherry MFI and percentage of mCherry-positive cells were observed in human CD4^+^ T cells, CD4^+^ CD11c^+^ dendritic cells and CD4^+^ monocytes, compared to human CD4^-^ cell subsets (Figs. 2E and EV2B,C). However, this CD4⁺-specific enrichment was not observed in huCD45⁺ cells isolated from the spleen or bone marrow. These findings show that CD4-NBs rapidly target blood CD4^+^ cells. Still, despite their presence in different organs, their capacity to target CD4^+^ cells is limited in peripheral tissues.

### CD4-directed nanoblades specifically edit CD4^+^ cell genomes

**Figure 3:**
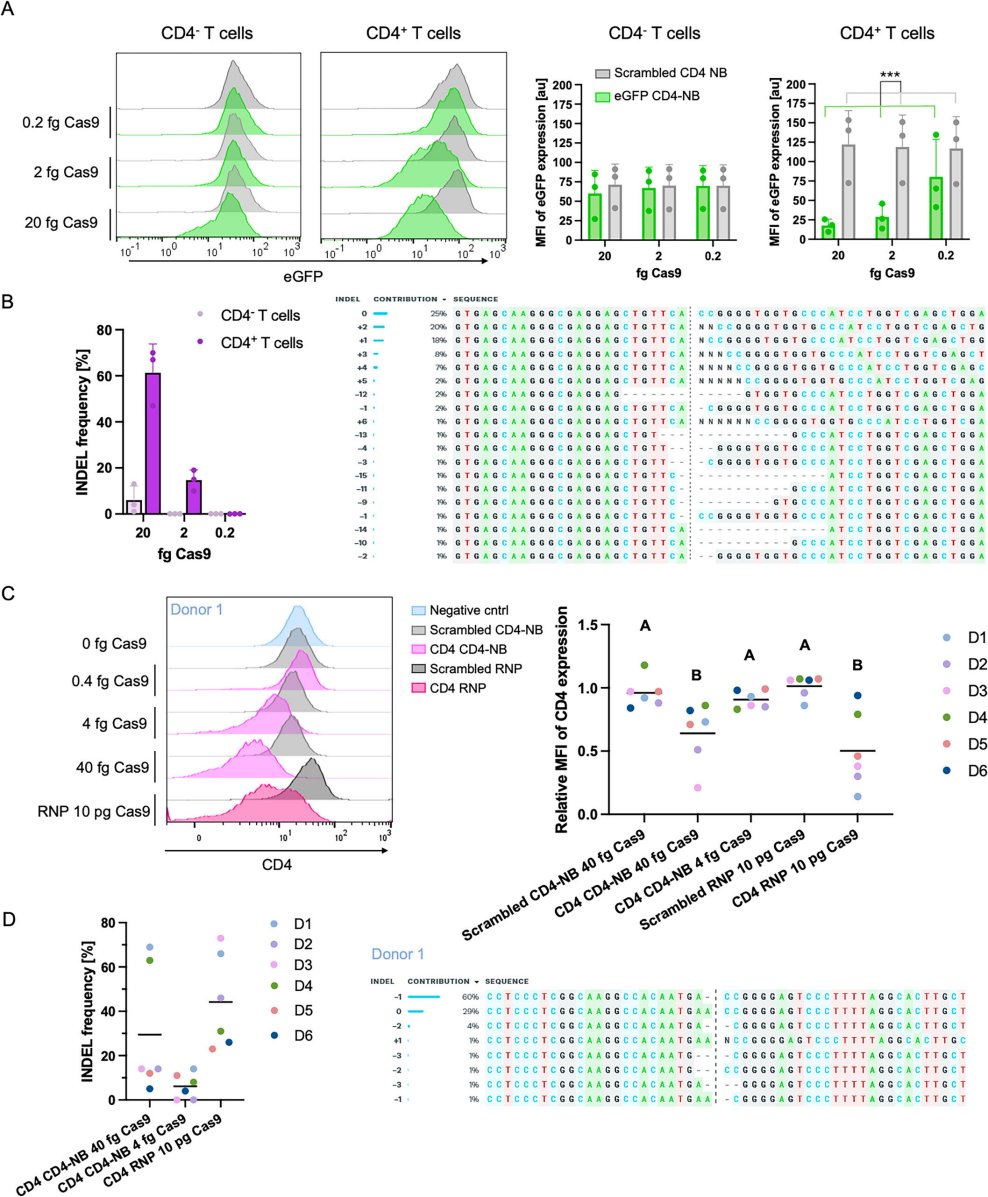
CD4-directed nanoblades specifically edit CD4^+^ cell genomes. (**A**) Flow cytometry histograms (left) and bar charts (right) of eGFP expression in CD4^+^ and CD4^-^ Sup-T1 cells 5 days post treatment with different doses of scrambled or eGFP CD4-NBs. Analysis was done with two-way ANOVA (*n* = 3 technical replicates of 3 experimental replicates, averages of each experiment are shown as individual dots, bars represent grand mean + SD, ***p < 0.001). (**B**) Bar chart of INDEL detection frequency at the *eGFP* locus of CD4^+^ and CD4^-^ Sup-T1 cells (*n* = 3 experimental replicates shown as individual dots, bars represent mean + SD) (left) and INDEL sequences and their corresponding introduction efficiencies at the Cas9 cut site from one biological replicate of CD4^+^ Sup-T1 cells treated with eGFP CD4-NBs (right). (**C**) Flow cytometry histograms (left) and dot plots with grand means (right) showing CD4 knockdown, expressed as CD4 MFI, in primary CD4^+^ cells following scrambled or CD4 CD4-NB transduction or RNP electroporation. Each dot represents the average of three technical replicates. One-way ANOVA was done followed by a Tukey post hoc test (*n* = 3 technical replicates of six donors). Different letters indicate significant differences (F_5,60_ = 11.812, *p* < 0.001). (**D**) Bar chart of INDEL detection frequency at the *CD4* locus of primary CD4^+^ cells (*n* = 6 biological replicates) (left) and INDEL sequences and their corresponding introduction efficiencies at the Cas9 cut site from CD4^+^ cells from donor 1 treated with CD4 CD4-NBs (right). Source data are available online for this figure.

CD4 cell specificity of CD4-NBs was further confirmed by treating a pool of CD4^+^ and CD4^-^ Sup-T1 cells carrying random integrations of the GFP reporter gene with different doses (0.2, 2, and 20 fg Cas9/cell) of CD4-NBs carrying either a single guide RNA (sgRNA) designed to target *eGFP* (eGFP CD4-NBs) or a scrambled sgRNA sequence as a control (scrambled CD4-NBs). eGFP CD4-NBs specifically and significantly knocked out eGFP protein expression in CD4^+^ Sup-T1 cells (F_1,47_ = 86.504, *p* < 0.001) but not in CD4^-^ Sup-T1 cells (F_1,47_ = 1.164, *p* = 0.286) in a dose-dependent manner, as analyzed by flow cytometry 5 days post treatment (Fig. 3A). Target cell editing activity was specific to the CD4 nanobody, as a CD4-NBs pseudotyped with MV Δ18Hmut and VSV Gmut did not result in detectable eGFP knockout in CD4^+^ nor CD4^-^ cells. Genetic knockout was confirmed by Sanger sequencing of PCR amplicons and Synthego inference of CRISPR edits analysis. Indeed, 61 ± 10% of eGFP sequences in the CD4^+^ cell pool treated at maximal dose (20 fg Cas9/cell) carried eGFP gRNA-specific insertions and deletions (INDELs) at the *eGFP* locus, compared to 6 ± 5% of those in the CD4^-^ cell pool (Fig. 3B, left panel). A range of different INDELs was observed around the gRNA cut site, ranging from 15 bp deletions (1% of total sequences) to +2 bp insertions (20% of total sequences) (Fig. 3B, right panel).

**Figure EV3:**
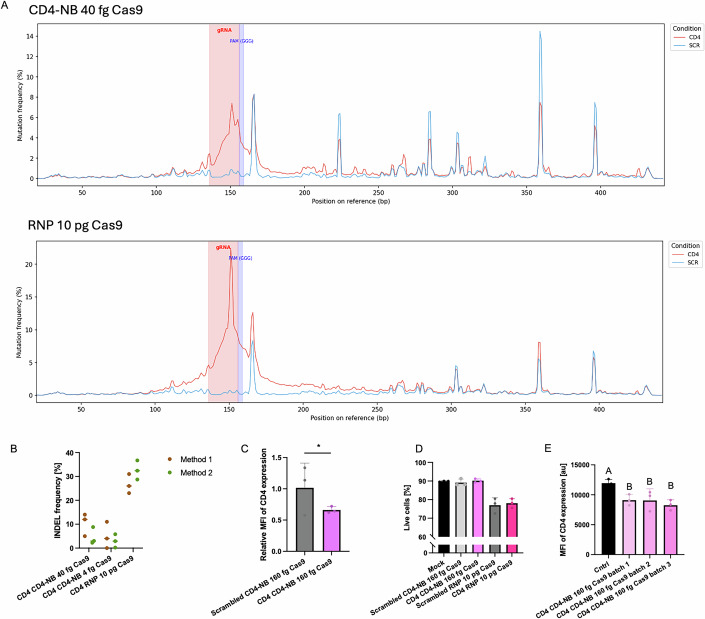
Additional analyses of CD4 gene editing by CD4-NBs in primary human CD4^+^ cells. (**A**) Mutation frequencies of *CD4* following treatment with CD4-NBs (upper panel) or sgRNA-Cas9 RNPs (lower panel) in primary CD4^+^ cells. The scrambled and CD4 gRNA conditions are depicted in blue and red, respectively. The gRNA and PAM sequences are shaded in red and blue on the graphs, respectively. (**B**) Bar chart representing INDEL detection frequency at the *CD4* locus following CD4-NB treatment (40 or 4 fg Cas9/cell) or sgRNA-Cas9 electroporation (10 pg Cas9/cell) using two different methods on PCR amplicons. Method 1: Sanger sequencing followed by tracking INDELs via Synthego Inference of CRISPR Edits analysis (Nakamae and Bono, 2022); method 2: Oxford Nanopore Technologies (ONT) sequencing to determine mutation frequencies 10 bp around the gRNA cut site of *CD4* (PathoSense, Gent, Belgium). Analysis was done using a two-way ANOVA (F_1,12_ = 0.086, *p* = 0.774, *n* = 3 biological replicates). (**C**) Bar chart representing CD4 knockdown, expressed as CD4 MFI, in primary CD4^+^ cells following a high dose of scrambled or CD4 CD4-NB transduction. Analysis was done using a one-way ANOVA (*n* = 3 technical replicates of three biological replicates, *p = 0.05). (**D**) Bar chart showing cell viability following different doses of CD4-NB transduction or sgRNA-Cas9 RNP electroporation (*n* = 3 technical replicates of 3 biological replicates). (**E**) Bar chart representing CD4 knockdown, expressed as CD4 MFI, in primary CD4^+^ cells following transduction with three different batches of CD4 CD4-NBs on one donor. Analysis was done with one-way ANOVA followed by a Tukey post hoc test (*n* = 3 technical replicates). Significant letters are indicated by different letters (*p* < 0.05, exact *p* values are reported in Appendix Table S4).

To further explore the utility of CD4-NBs, we assessed their ability to genetically edit primary CD4^+^ cells of six different donors. Primary CD4^+^ cells were incubated with different doses (0.4, 4 and 40 fg Cas9/cell) of CD4-NBs carrying a gRNA designed to target *CD4* (CD4 CD4-NBs) or a scrambled gRNA sequence as a control (scrambled CD4-NBs) and CD4 expression (MFI) was analyzed 5 days later using flow cytometry. In parallel, CD4 sgRNA- or scrambled sgRNA-Cas9 RNPs (dose of 10 pg Cas9/cell) were electroporated into cells as positive and negative controls, respectively. As expected, cells electroporated with CD4 sgRNA-Cas9 RNPs showed significantly lower CD4 expression compared to controls (*p* < 0.05, Fig. 3C). Interestingly, treatment with the highest dose of CD4 CD4-NBs resulted in a similar knockout of CD4 expression compared to electroporation of CD4 sgRNA-Cas9 RNPs. Still, the highest dose of CD4-NBs contained only 40 fg Cas9 per cell while the RNP EP delivered a 250-fold higher concentration of Cas9 per cell (10 pg/cell), highlighting the efficacy of CD4-NBs to edit primary CD4 cells. *CD4* genetic knockout following treatment with CD4 CD4-NBs was confirmed in all six donors by Sanger sequencing of PCR amplicons and Synthego inference of CRISPR edits analysis (Fig. 3D). However, there was a larger donor variability as compared to CD4 protein expression, as the CD4 gene of donors 1 and 4 contained over 60% of INDELs, while that of the other donors only 5–14%. To exclude potential bias introduced by Sanger sequencing and subsequent sequence trace decomposition, we additionally assessed the mutation frequency around the Cas9 cut site in PCR amplicons from three donors using Oxford Nanopore Technologies (ONT) sequencing (Fig. EV3A,B). The percentage of INDELs detected did not significantly differ between both methods (F_1,12_ = 0.086, *p* = 0.774). Still, this does not rule out potential PCR bias. Alternatively, a disproportionate impact of frameshift mutations or allelic effects in certain donors could explain these variations. INDELs were observed around the gRNA cut site in all three donors, ranging from 20 bp deletions to 4 bp insertions, with the 1 bp deletion being the most frequent (up to 60% of total sequences) (Fig. 3D, right panel).

Finally, we confirmed even higher CD4 knockout efficiencies at an increased CD4-NB dose (160 fg Cas9/cell) without significant toxicity compared to control conditions (Fig. EV3C,D). In contrast, sgRNA-Cas9 RNP-electroporated cells exhibited ~10% lower viability. We also tested two additional batches of CD4 CD4-NBs on one donor to ensure reproducibility of CD4 knockout when using CD4-NBs standardized for Cas9 dose per cell (Fig. EV3E). These data further support the utility of CD4-NBs for targeted editing of primary CD4⁺ T cells.

### CD4-directed nanoblades hamper HIV infection in vitro

**Figure 4:**
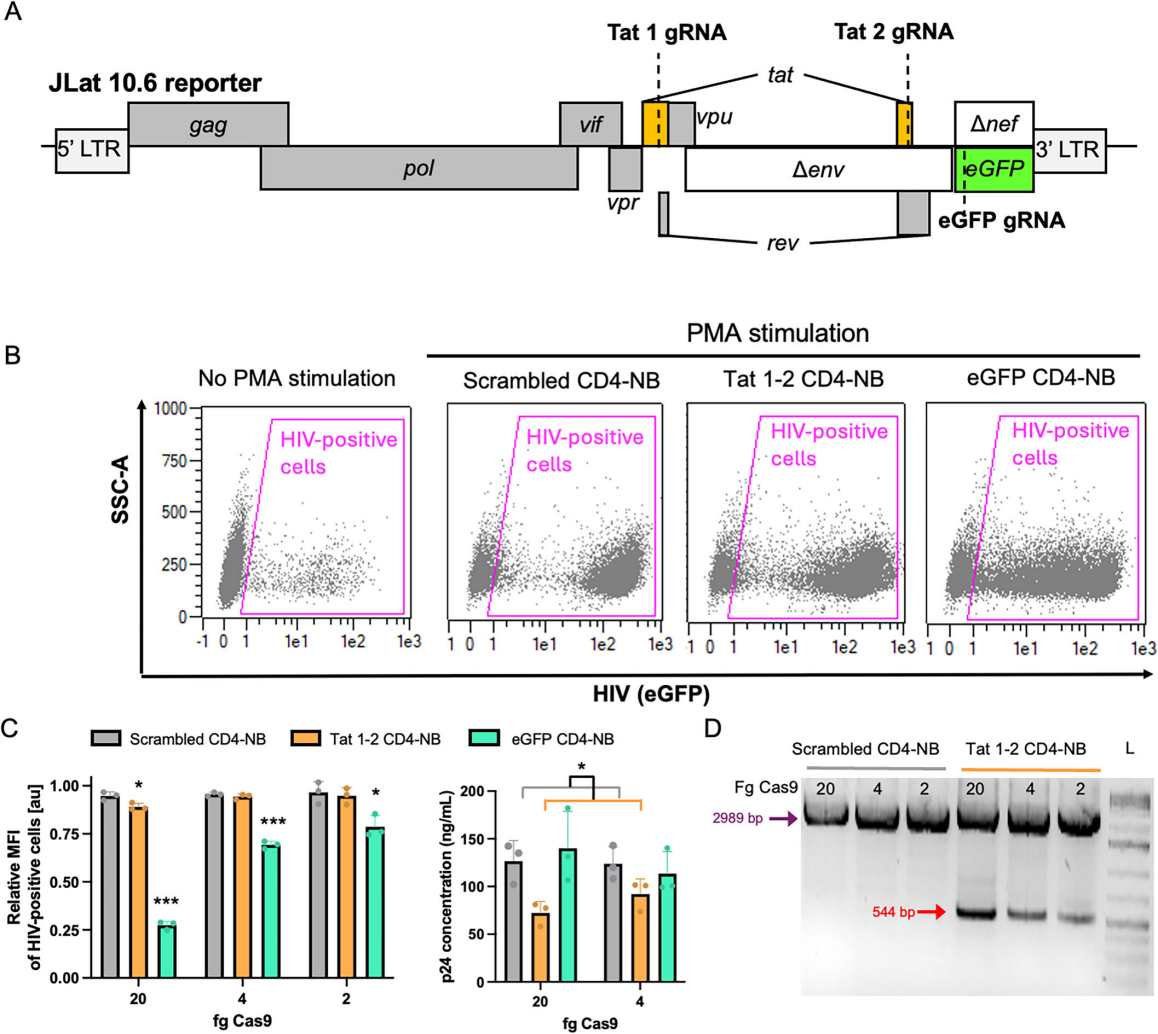
CD4-directed nanoblades target HIV proviral DNA of latently infected cells. (**A**) Schematic representation of the HIV proviral genome integrated in J.Lat 10.6 cells with the approximate location of each gRNA. (**B**) Representative scatter dot plots analyzed by flow cytometry for HIV gene (eGFP) expression of live J.Lat 10.6 cells either non-stimulated or stimulated with PMA and treated with scrambled, tat 1-2, or eGFP CD4-NBs. (**C**) Left panel: bar charts representing relative eGFP MFI of PMA-stimulated J.Lat 10.6 cells treated with different CD4-NBs at different dosages (normalized to mock-transduced cells). Analysis was done using two-way ANOVA (*n* = 3 experimental replicates shown as individual dots, bars represent mean + SD). Due to interaction effects, one-way ANOVA followed by a Dunnett post hoc test was conducted per Cas9 concentration. *p = 0.023 and 0.011 for Tat 1-2 CD4 NBs at 20 fg Cas9 and eGFP CD4-NBs at 2 fg Cas9, respectively, ***p < 0.001. Right panel: HIV p24 concentration in the supernatant of these cells. Two-way ANOVA indicated a significant effect of treatment (F_5,12_ = 3.494, *p* = 0.009) and was followed by a Dunnett post hoc test (*n* = 3 experimental replicates shown as individual dots, bars represent mean + SD) *p = 0.014. (**D**) Agarose gel electrophoresis of PCR products for the *tat* locus of J.Lat 10.6 cells transduced with different doses of scrambled or tat 1-2 CD4-NBs. The purple arrow depicts the full-length PCR product (2989 bp), while the red arrow shows the 544 bp excised fragment. Source data are available online for this figure.

Next, we generated CD4-NBs aimed to eliminate HIV proviral DNA by incorporating two gRNAs directed against the *tat* locus (tat1 and tat2). These gRNAs have previously been designed to target a broad range of HIV strains and cause multi HIV exon disruption by simultaneously targeting *tat*, *rev*, and *env* without causing off-target effects (Herskovitz et al, 2021). Tat 1-2 CD4-NBs were first applied to a latently infected T cell line that bears a single proviral copy of an *env*- and *nef*-deficient HIV variant on chromosome 9 (i.e., J.Lat 10.6 cells) (Chung et al, 2020). Instead of *nef*, *eGFP* is incorporated in the HIV proviral genome, of which transcription is driven by the viral long terminal repeat (LTR) promoter (Fig. 4A). Scrambled and eGFP CD4-NBs were included as negative and positive controls, respectively. The binding regions of tat1, tat 2, and eGFP gRNAs on the J.Lat 10.6 HIV proviral genome are depicted in Fig. 4A. Treatment with PMA stimulates LTR-mediated full-length genome transcription, including that of *eGFP*, which is further stimulated by the action of HIV tat. Three days post-PMA treatment, relative MFI of eGFP (normalized to mock-transduced cells) was significantly lower in eGFP CD4-NB-treated J.Lat cells compared to control-treated cells at all tested concentrations (*p* = 0.011 for 2, *p* < 0.001 for 4, and *p* < 0.001 for 20 fg Cas9/cell), while tat 1-2 CD4-NBs exhibited a significant effect on eGFP expression only at the highest concentration (*p* = 0.023) (Fig. 4B,C left panel). This is likely because eGFP expression is still driven by PMA-stimulated LTR action in the absence of HIV tat. HIV p24 concentrations in cell supernatants were significantly (*p* = 0.014) lower only in tat 1-2 CD4-NB-treated conditions compared to control, meaning that *tat* and *rev* disruption affected efficient HIV capsid production (Fig. 4C, right panel). We then confirmed viral gene excision by nested PCR for the *tat* locus on genomic DNA. A predictable 544 bp excision band was present in cells treated with tat 1-2 CD4-NBs, and the band intensity decreased with decreasing concentrations of Cas9 (Fig. 4D).

**Figure 5:**
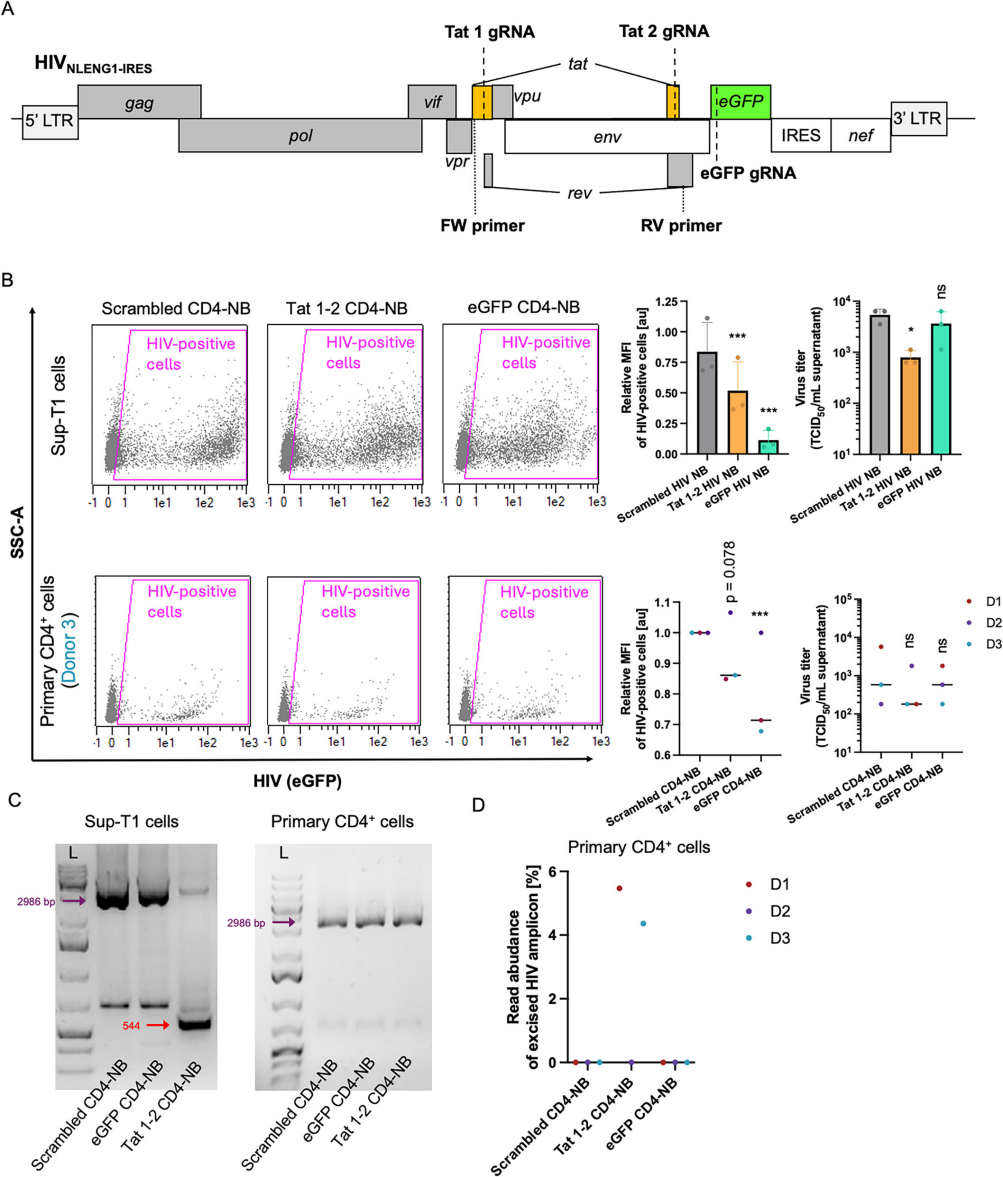
CD4-directed nanoblades hamper active HIV infection in CD4^+^ cells. (**A**) Schematic representation of the HIV_NLENG1-IRES_ proviral genome with the approximate location of each gRNA. (**B**) Left panels: Representative scatter dot plots analyzed by flow cytometry for HIV gene (eGFP) expression of live Sup-T1 cells (upper panels) and primary CD4^+^ cells (lower panels) either mock- or HIV-infected and treated with scrambled, tat 1-2, or eGFP CD4-NBs. (**B**) Middle panels: bar chart representing mean + SD and dot blot with grand median representing eGFP MFI of HIV-infected Sup-T1 cells (upper panel) and primary CD4^+^ cells (lower panel) treated with different CD4-NBs at a dose of 40 fg Cas9/cell. Analysis was done using one-way ANOVA followed by a Dunnett post hoc test (Sup-T1 cells: F_2,19_ = 42.703, *n* = 3 technical replicates of 3 experimental replicates. Primary CD4^+^ T cells: F_2,17_ = 16.283, *n* = 3 technical replicates of 3 biological replicates, ***p < 0.001). Right panels: virus titer, expressed as TCID_50_/mL, in supernatant of HIV_NLENG1-IRES_-infected Sup-T1 cells (bar chart representing mean + SD) and primary CD4^+^ cells (dot blot with grand medians) transduced with different CD4-NBs. Analysis was done using one-way ANOVA (Sup-T1 cells: F_2,6_ = 5.178, *n* = 3 experimental replicates, *p = 0.033, ns: *p* = 0.431. Primary CD4^+^ T cells: F_2,6_ = 1.406, *n* = 3 biological replicates, ns: *p* = 0.420 and 0.260 for tat 1-2 and eGFP CD4-NBs, respectively. (**C**) Agarose gel electrophoresis of PCR products for the *tat* locus of HIV_NLENG1-IRES_-infected Sup-T1 cells and primary CD4^+^ cells treated with different CD4-NBs. The purple arrow depicts the full-length PCR product (2989 bp), while the red arrow shows the 544 bp excised fragment. (**D**) Relative abundance of excised HIV gene fragment over intact fragment in CD4-NB treated HIV-infected primary CD4^+^ T cells, as identified using nanopore sequencing (*n* = 3 biological replicates). Source data are available online for this figure.

**Figure EV4:**
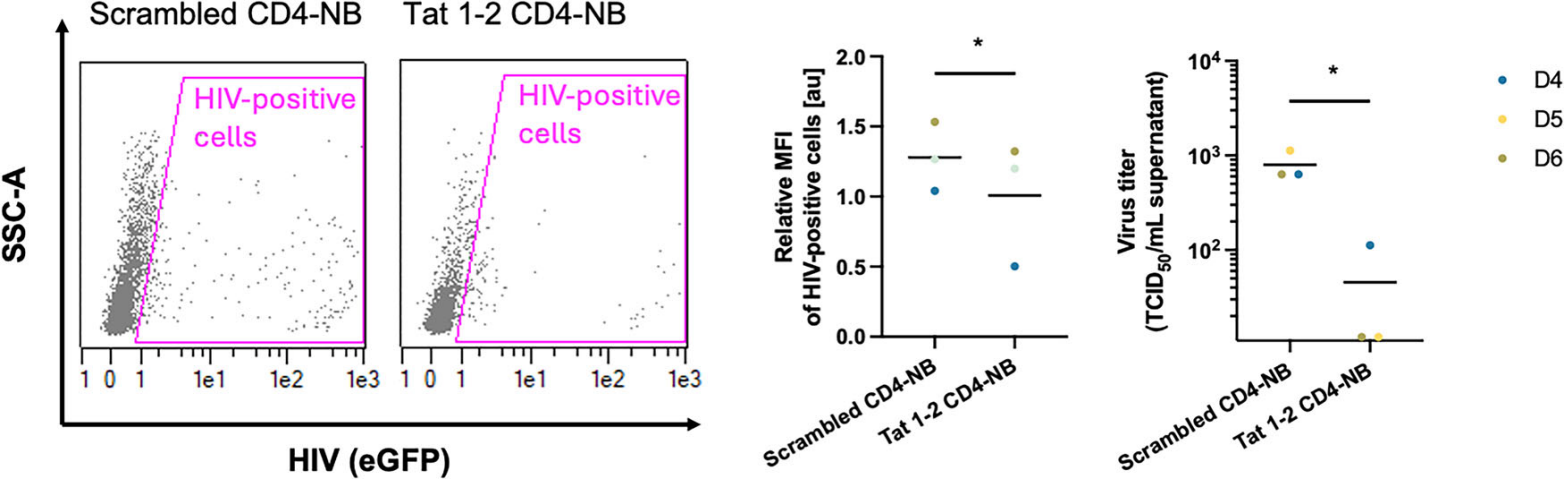
CD4-NB treatment of three additional donors following HIV infection at lower MOI (0.01). Left panel: Representative scatter dot plots analyzed by flow cytometry for HIV gene (*eGFP*) expression of live primary CD4^+^ cells treated with scrambled or tat 1-2 CD4-NBs. Middle panel: dot blot with grand median representing average eGFP MFI of HIV-infected primary CD4^+^ cells treated with different CD4-NBs at a dose of 40 fg Cas9/cell. Analysis was done using one-way ANOVA (three technical replicates on three biological replicates, *p = 0.042). Right panel: dot blot with grand mean representing virus titer, expressed as TCID_50_/mL, in supernatant of HIV_NLENG1-IRES_-infected primary CD4^+^ cells treated with different CD4-NBs at a dose of 40 fg Cas9/cell. Analysis was done using an independent samples *t*-test (three biological replicates, *p = 0.029).

After demonstrating the ability of tat 1-2 CD4-NBs to excise latent replication-incompetent HIV genomes and suppress viral protein production, we next investigated whether they also inhibit replication-competent HIV infection in Sup-T1 cells and primary CD4^+^ cells. To do so, we first infected these T cells with a full-length, replication-competent HIV NL_4.3_ strain expressing eGFP under the control of the viral LTR promoter (HIV_NLENG1-IRES_, Fig. 5A) prior to CD4-NB treatment. Tat 1-2 CD4-NB treatment clearly hampered HIV gene expression, as eGFP MFI was significantly lower in cells treated with tat 1-2 CD4-NBs compared to control in Sup-T1 cells (*p* < 0.001) as well as in two out of three donors of primary CD4^+^ cells (*p* = 0.078) (Fig. 5B). As expected, eGFP CD4-NBs directly targeting the eGFP reporter gene of HIV_NLENG1-IRES_ knocked down eGFP expression to an even higher extent in both cell types. More importantly, tat 1-2 CD4-NB-treated cells, but not eGFP CD4-NB-treated cells, produced 6- to 17-fold less infectious HIV particles compared to control in Sup-T1 and primary CD4^+^ cells (two out of three donors), respectively (Fig. 5B). Viral gene excision was confirmed in genomic DNA from Sup-T1 cells treated with tat 1-2 CD4-NBs by gel electrophoresis of PCR amplicons (Fig. 5C). The excised fragment could not be visualized by gel electrophoresis in tat 1-2 CD4-NB-treated primary CD4^+^ T cells, but was identified in 4.36–5.46% of their amplicon reads via ONT sequencing (Fig. 5D). In a repeat experiment using a fourfold lower MOI on three new donors, tat 1-2 CD4-NBs significantly (p < 0.05) lowered HIV gene expression as well as infectious virus production (Fig. EV4) compared to scrambled CD4-NBs. These results cumulatively support the utility of tat 1-2 CD4-NBs in hampering active HIV infection.

### Tat 1-2 CD4-directed nanoblades lower plasma HIV viremia levels in vivo, but cannot eliminate HIV from tissues

**Figure 6:**
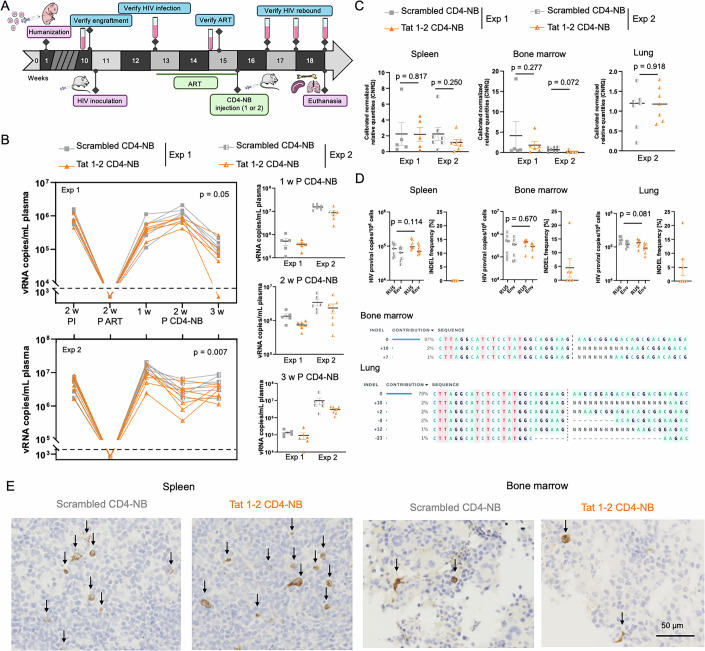
CD4-directed nanoblades lower plasma HIV viremia levels in vivo, but cannot eliminate HIV from tissues. (**A**) Study design of the in vivo experiment, including different performed procedures (purple), treatments (green), and sampling periods (blue). (**B**) Plasma viral loads of individual animals for the two treatment groups (scrambled CD4-NBs, shown in gray, and tat1-2 CD4-NBs, shown in orange) were assayed at 2 weeks (w) post infection (PI), 2 weeks post ART initiation (P ART), 1, 2, and 3 weeks post ART cessation and CD4-NB treatment (P CD4-NB) for HIV RNA. Data from two separate experiments are shown (exp 1 and 2). The dashed lines represent the limit of detection. Analysis was done using repeated measures ANOVA (exp 1: F_1,9_ = 4.824, *n* = 5–6 mice; exp 2: F_1,11_ = 11.049, *n* = 7 mice). The scatter plots on the right show the mean ± SEM. (**C**) Calibrated normalized relative quantities (CNRQ) of HIV RNA loads, as measured with RT-qPCR, in spleen, bone marrow, and lungs of individual animals for the two treatment groups (scrambled CD4-NBs, shown in gray, and tat 1-2 CD4-NBs, shown in orange) of the two individual experiments (exp 1 and 2) or exp 2 only (lungs). Analysis was done using one-way ANOVA (spleen exp 1: F_1,8_ = 0.057, *n* = 5 mice; spleen exp 2: F_1,12_ = 1.463, *n* = 7 mice; bone marrow exp 1: F_1,8_ = 1.358, *n* = 5–6 mice; bone marrow exp 2: F_1,12_ = 3.889, *n* = 7 mice; lung exp 2: F_1,12_ = 0.011, *n* = 7 mice). Scatter plots show the mean ± SEM. (**D**) Impact of CD4-NBs on HIV proviral DNA in spleen, bone marrow, and lungs of individual animals for the two treatment groups of exp 2. Upper panel, left graphs: HIV proviral DNA loads represented by copies *RU5* and *env* per million human cells. Analysis was done using a repeated measures ANOVA (spleen: F_1,12_ = 2.446, *n* = 7 mice; bone marrow: F_1,12_ = 23.982, *n* = 6–7 mice; lung: F_1,12_ = 250.548, *n* = 7 mice). Scatter plots show the median. Upper panel, right graphs: INDEL frequencies detected at the tat 1 locus for tat 1-2 CD4-NB-treated animals. Middle and lower panel: corresponding INDEL introduction efficiencies at the Cas9 cut site from bone marrow, and lungs of mice treated with CD4 CD4-NBs. Scatter plots show the mean ± SEM. (**E**) Representative images of HIV env-positive cells (indicated by arrows) in spleen and bone marrow of mice upon administration of scrambled or tat 1-2 CD4-NBs in exp 1. Source data are available online for this figure.

**Figure EV5:**
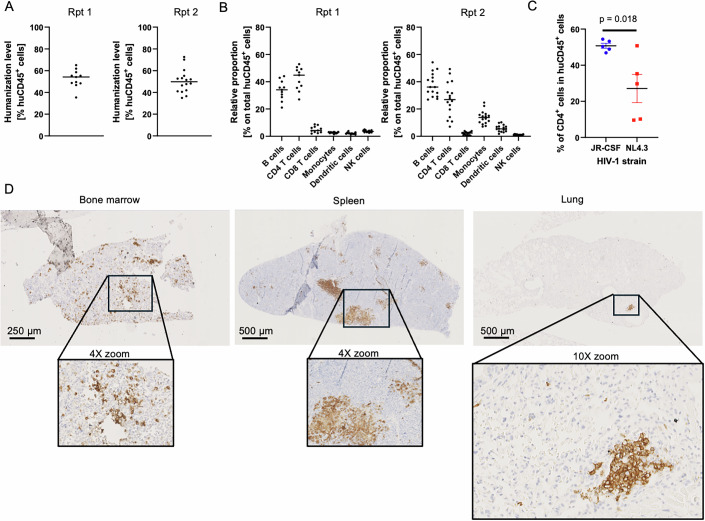
Characterization of humanized mice used for HIV infection experiments. (**A**) Humanization levels of mice prior to HIV_JR-CSF_ infection shown as % of huCD45^+^ cells over total muCD45^+^ and huCD45^+^ cells (*n* = 11 and 16 for repeat [rpt] 1 and 2, respectively). (**B**) Relative proportion of specific human immune cell subsets in these humanized mice represented as % of specific cell types over total huCD45^+^ cells (*n* = 11 and 16 for rpt 1 and 2, respectively). (**C**) CD4 cell levels in humanized mice upon infection with HIV_JR-CSF_ or HIV_NL4.3_. Analysis was done using an independent samples *t*-test (*n* = 5 biological replicates). The scatter plot shows the mean ± SEM. (**D**) Expression of human HLA-DR in the spleen and bone marrow of a representative humanized mouse (scrambled CD4-NB) confirms human cell reconstitution in different tissues at the end of the experiment.

The robust in vitro gene-editing capacity specific to CD4^+^ cells and antiviral activity in primary cells, along with their ability to reach HIV tissue reservoir sites such as spleen and bone marrow, suggested that CD4-NBs might be a promising curative strategy against HIV. Therefore, we next investigated their potential to suppress HIV infection in an in vivo model. As HIV does not infect murine cells, we first humanized NSG-SGM3 mice with cord blood-derived huCD34^+^ hematopoietic stem cells. A schematic experimental overview is given in Fig. 6A. Correct engraftment was verified in blood 10 weeks later, and mice with humanization levels above 20% were selected for infection experiments (Fig. EV5A,B). In addition, human cell reconstitution in tissues (spleen, bone marrow, and lungs) was also confirmed at the end of the experiments through immunohistochemical detection of human HLA-DR (Fig. EV5D). Mice were infected with HIV_JR-CSF_, a replication-competent CCR5-tropic strain known to infect humanized mice and which depletes CD4^+^ cells, our target cells, to a significantly (*p* = 0.018) lower extent compared to HIV_NL4.3_ (Fig. EV5C) (Adams et al, 2021). Two weeks post infection, all mice exhibited plasma viral RNA loads exceeding 10^5.65^ viral (v) RNA copies/mL plasma (Fig. 6B, upper panel). Next, mice were put on antiretroviral therapy for 2 weeks to restrict ongoing HIV replication and lower absolute proviral DNA loads, a strategy that previously already enhanced CRISPR-Cas9-based HIV clearance (Dash et al, 2019). In addition, ART administration restores patient CD4^+^ cell numbers, our primary target cells. All plasma HIV levels dropped below the limit of detection before injecting mice with scrambled (control) or tat 1-2 CD4-NBs. During the first experiment, mice received a single dose of 300 ng Cas9-loaded CD4-NBs prior to ART cessation. Plasma viral rebound was monitored over time and occurred in all mice. Interestingly, plasma viremia levels were significantly lower in tat 1-2 CD4-NB-treated mice compared to control (*p* = 0.05) over time, and in one tat 1-2 CD4-NB-treated mouse, vRNA copies in plasma even dropped below the detection limit at the end of the experiment (Fig. 6B, upper panel). In contrast, vRNA loads in the spleens and bone marrow of tat 1-2 CD4-NB-treated mice did not significantly differ from control-treated mice (Fig. 6C). Similarly, the number of HIV env-positive cells did not significantly differ between the organs of both groups (Fig. 6E). Repeated CRISPR-Cas9 administration has already been proven to enhance its anti-HIV activity (Liu et al, 2021). Therefore, in a second experiment, we verified if increasing the number of doses would result in better HIV clearance from spleen, bone marrow, and lungs. All mice received two doses of 300 ng Cas9 CD4-NBs, 2 days apart, prior to ART cessation. Although plasma viremia was again significantly lower in mice treated with tat 1-2 CD4-NBs compared to control (Fig. 6B, lower panel), there was again no significant difference in vRNA loads in both organs, nor in the lungs between the two groups. However, *p* values were lower than in the single-dose experiment, approaching significance in bone marrow (*p* = 0.072, Fig. 6C), suggesting a potential dosing-dependent tissue effect. We next assessed the effect of CD4-NBs on HIV proviral DNA by quantifying HIV *RU5* and *env* copy numbers per 10^6^ human cells (Fig. 6D, left graphs) and by analyzing INDEL formation around tat 1 gRNA locus. No significant differences in proviral DNA loads were observed between treatment groups across all three organs, although a trend toward lower HIV loads was noted in the lungs (*p* = 0.081). Interestingly, INDELs at the tat 1 gRNA locus were detected in the bone marrow and lungs of three out of seven mice treated with tat 1-2 CD4-NBs, but not in the six mice receiving scrambled CD4-NBs (Fig. 6D right graphs, middle and lower panels). These findings highlight the potential of CD4-NBs for HIV suppression in vivo.

## Discussion

Antiretroviral therapy (ART) has transformed HIV from a fatal disease into a manageable chronic condition. Still, HIV infection remains non-curative and requires lifelong ART adherence. Worldwide funding for HIV cure programs is facing increasing pressure (Cavalcanti et al, 2025). In addition, challenges such as drug resistance, adverse drug effects, and inability to clear persistent viral reservoirs highlight the urgent need for alternative cure options that can achieve prolonged viral remission or elimination. Removal of HIV proviral genomes from latently infected CD4^+^ cells using gene therapy represents a promising route toward a cure (Das et al, 2019; Dash et al, 2019, 2023). CRISPR-Cas9-mediated excision of HIV proviral DNA using AAV delivery has already been shown to reduce and even clear HIV infection in mouse models and is currently under active clinical investigation (NCT02140944, NCT03164135, and NCT03666871) (Dash et al, 2019, 2023). However, complete HIV clearance was achieved in only a subset of treated mice, likely due to suboptimal delivery of the CRISPR system to latently infected CD4^+^ T cells. A challenge in this strategy is that enough HIV proviral copies need to be cleared, as in theory, one remaining copy could lead to HIV rebound. Here, we sought to increase the efficacy of gene therapy by directing the CRISPR-Cas9 system specifically toward HIV target cells, i.e., CD4^+^ cells, thereby minimizing RNP uptake by bystander cells. To do so, we aimed to combine the efficacy and in vivo applicability of viral delivery with the safety of transient Cas9 expression, avoiding the risks associated with viral genome integration and prolonged expression of the editing agent by using virus-like particles (VLPs) devoid of any viral genome (Banskota et al, 2022; Hamilton et al, 2021). More specifically, we constructed v3 Cas9-VLPs decorated with anti-CD4 nanobodies, named CD4-NBs, which enable specific targeting of CD4^+^ cells, including T lymphocytes and myeloid cells, the main HIV reservoirs. A possible critique is that CD4 is known to be downmodulated upon HIV infection. However, this is mostly reversed by prior ART administration, especially when given early upon infection (Le et al, 2013; Sponaugle et al, 2023). At present, no universal marker for HIV-infected cells is known. CD4 remains the most reliable surface marker expressed on all HIV-susceptible cells, including lymphocytes and certain myeloid subsets. If a specific marker for HIV-infected cells is identified in the future, the versatility of our platform would permit rapid replacement of the targeting ligand (e.g., anti-CD4 nanobodies) with a more selective alternative.

CD4-NBs specifically bind, enter and edit CD4^+^ cells, with minimal uptake observed in CD4^−^ cells, including phagocytes, in vitro and in vivo in blood. This highlights their potential to reduce the bystander effect and underscores their advantage over previously developed CD4-targeted delivery vehicles. Indeed, murine CD4 antibody-targeted lipid nanoparticles (LNPs), despite selective CD4 targeting to the CD4^+^ T cell populations, were still taken up preferentially by dendritic cells and macrophages (Tombácz et al, 2021b). There was no distinction between CD4^+^ and CD4^-^ myeloid cells because murine myeloid cells do not express CD4 at the cell surface (Moore et al, 1992). For other murine CD4 antibody-coated LNPs or for human CD4-targeted delivery vehicles, like human CD4 DARPIN-pseudotyped lentiviruses and CD4 nanobody-modified AAVs, phagocyte uptake was not investigated (Hamann et al, 2021; Zhou et al, 2015; Jamali et al, 2019; Ramishetti et al, 2015; Cao et al, 2018).

Despite efficient targeting of CD4^+^ cells in blood in vivo, CD4-NBs did not achieve similar specific binding to resident CD4^+^ cells in lymphoid tissues. A previous study reported selective CD4^+^ cell targeting in tissues using human CD4-DARPIN-coated lentiviruses (Zhou et al, 2015). However, their analysis was limited to the CD3^+^ human T cell population, excluding other key human immune cells such as dendritic cells and monocytes, which are known to be highly phagocytic. This could have been related to the mouse strain they used, as NSG mice do not allow the engraftment of human myeloid cells. Instead, we used NSG-SGM3 mice, which do enable human myeloid cell engraftment (Billerbeck et al, 2011). If we only compare the mCherry signal in CD3^+^ T cells of lymphoid tissues, we also observed significantly more uptake in CD4^+^ T cells compared to CD8^+^ T cells (Fig. 2E), suggesting similar selectivity in tissues compared to CD4-DARPIN lentiviruses. In addition, their protocol required prestimulation of T cells via OKT3 or IL-7 injection prior to lentivirus administration, which complicates clinical translation, whereas our CD4-NBs required no such stimulation. Similarly, as stated above for in vitro targeting, myeloid cell compartments were either not analyzed or showed comparable uptake of CD4-targeted delivery vehicles in vivo in previous studies (Tombácz et al, 2021a; Ramishetti et al, 2015). Tissue-resident immune cells may exhibit increased nonspecific uptake of particles, and inefficient extravasation could hinder CD4-NBs from accessing their target CD4^+^ cells within tissues (Gustafson et al, 2015). Additionally, sequestration by non-lymphoid organs such as the liver and kidneys may further reduce their bioavailability. Strategies to improve tissue targeting could include the incorporation of ligands with high affinity for capillary wall proteins (e.g., addressins) to promote vascular extravasation of VLPs, leading to increased local tissue concentrations. Furthermore, the addition of stealth-enhancing molecules such as polyethylene glycol (PEG) or “don’t eat me” signals such as CD47 may prolong circulation time and reduce clearance by liver, kidneys, and phagocytes, thereby improving delivery efficiency to tissue-resident CD4^+^ cells (Xu et al, 2025; Rohovie et al, 2017). Nonetheless, CD4-NBs represent a promising platform for CD4-targeted CRISPR-Cas9 delivery, demonstrating superior performance over classical ex vivo electroporation by achieving comparable editing efficiencies with lower toxicity in primary CD4^+^ T cells with approximately 250-fold lower Cas9 doses. This higher efficacy might also be related to the extended time of cargo delivery by CD4-NBs, whereas RNP uptake during electroporation is restricted to the brief duration of the electric pulse. Additionally, CD4-NBs also achieved gene knockout in up to 69% of primary CD4^+^ T cells. Although this is higher than the 15% achieved at the highest dose with earlier platforms (Hamilton et al, 2021), these systems are not directly comparable, as the latter co-delivers a transgene that may influence Cas9 delivery efficiency.

To abrogate HIV infection, we employed a dual gRNA strategy targeting the highly conserved HIV *tat* locus. Among a library of gRNAs, tat1 and tat2 gRNAs showed the highest on-target activity across multiple HIV strains with no detectable off-target effects in the human genome (Herskovitz et al, 2021). Moreover, this region is the only one allowing to disrupt three essential HIV genes (i.e., *tat*, *rev*, and *env*) within overlapping open reading frames, thereby enhancing proviral inactivation and reducing the likelihood of viral escape. While LTR-directed CRISPR approaches aim to excise the entire HIV genome, multiple studies have shown inconsistent success, as double-strand breaks are frequently repaired by non-homologous end joining (NHEJ), leading to INDELs with little viral genome-disrupting effects in the LTR region (Yoder and Bundschuh, 2016; Mefferd et al, 2018). In contrast to targeting non-coding regions like the LTR or single genes such as *gag*, introducing INDELs or gene excisions within conserved and essential open reading frames, such as the *tat* locus, has a greater functional impact on proviral inactivation (Herskovitz et al, 2021; Mefferd et al, 2018; Ophinni et al, 2020). In line with these studies, tat 1-2 CD4-NBs induced genome excision in both replication-competent and replication-incompetent HIV proviruses, effectively suppressing viral gene (*eGFP*) expression and, more importantly, the production of viral particles, as *tat*, *rev*, and *env* are indispensable for productive infection. Interestingly, our study is the first to demonstrate the efficacy of a CRISPR-Cas9 strategy targeting *tat* in replication-competent HIV within primary CD4^+^ T cells, cells that are notoriously difficult to transfect, highlighting the potential of CD4-NBs as a curative approach for HIV. While CD4 CD4-NBs edited *CD4* in 6 out of 6 primary CD4 T cell donors, HIV gene excision or editing was achieved only in two out of three donors using tat 1-2 or eGFP CD4 NBs. This lower efficacy may be related to viral-mediated downregulation of surface CD4 expression in actively HIV-infected T cells. Still, from a therapeutic perspective, this limitation may be mitigated in vivo, as CD4 expression is known to be restored upon administration of antiretroviral therapy (ART).

Indeed, in vivo, CD4-NB-treated mice following ART pretreatment exhibited reduced plasma viremia, evidence of proviral gene disruption and a trend toward lower HIV proviral loads in tissues compared to controls, consistent with both in vitro inactivation of replication-competent HIV and the observed in vivo targeting of CD4^+^ cells. We hypothesize that the impact on proviral DNA may have been more pronounced at earlier time points, resulting in prolonged lower plasma viral loads. However, after three weeks, new rounds of infection by wild-type HIV might partially mask these effects in our analyses. Still, these findings underscore the importance of enhancing tissue-specific CD4^+^ cell targeting, particularly within lymphoid tissues that harbor key HIV reservoirs and fuel ongoing viremia. Our findings establish a proof-of-concept, but do not yet demonstrate direct clinical applicability. Additional advances in delivery and efficacy will be required before clinical implementation can be considered. For example, besides multiple dosing, improving tissue penetration may even further suppress viral loads following treatment. Another strategy could be to additionally incorporate CCR5-specific gRNAs in CD4-NBs. CCR5 is the primary co-receptor for HIV transmission and replication in vivo (Clapham and McKnight, 2001), and individuals with a homozygous CCR5Δ32 deletion are naturally resistant to infection. Still, CCR5 editing alone is insufficient for long-term viral control, as shown in clinical trials using ex vivo zinc-finger-edited T cells, which failed to achieve durable HIV remission in PLWH (Tebas et al, 2014). A dual editing strategy of HIV proviral DNA and *CCR5* has previously demonstrated efficacy in eliminating HIV from infected humanized mice using AAV-based delivery (Dash et al, 2023). However, due to the limited packaging capacity of AAVs, two separate vectors were required, which poses challenges for clinical translation.

Achieving a functional or sterilizing cure for HIV will likely require a combination of therapeutic approaches. In this context, CD4-NBs offer a promising strategy to reduce the initial HIV proviral reservoir, prior to HIV shock and kill strategies. For instance, a shock strategy could be mediated by tat-encoding LNPs our group previously reported (Pardons et al, 2023) and followed by the administration of anti-HIV CAR T cells as a kill strategy. While broad latency reversal in PLWH with a large viral reservoir may pose risks, preconditioning with CD4-NBs could reduce proviral burden to levels manageable by the autologous anti-HIV CAR T cells or perhaps the host’s immune system. Interestingly, defective virus in elite controllers is thought to prime the immune system to eliminate cells harboring replication-competent HIV proviruses (Jiang et al, 2020). CD4-NBs also do not completely abolish all HIV genes, leaving some viral genes transcribed and translated, thereby promoting immune-mediated clearance in PLWH. Finally, besides as a curative strategy for HIV, CD4-NBs could also be used for treating other diseases requiring in vitro or in vivo CD4^+^ cell targeting, such as autoimmune disorders or T cell malignancies (Horwitz et al, 2021).

Together, we developed a CD4^+^ cell-specific genome editing vehicle capable of targeting human blood CD4^+^ cells in vitro and in vivo. Equipping CD4-NBs with mosaic HIV *tat*-directed gRNAs enabled efficient HIV gene knockout and viral suppression in vitro and in vivo. Still, a limitation of our study is the low in vivo efficacy observed. Nevertheless, with further optimization for tissue targeting and combination with other curative approaches, CD4-NBs hold significant promise toward a definitive HIV cure.

## Methods

**Table:** Reagents and tools table

Reagent/resource	Reference or source	Identifier or catalog number
**Experimental models: Cell lines**		
HEK293T cells	ATCC	CRL-3216
TZM-bl cells	NIH HIV Reagent Program	ARP-8129
J-Lat 10.6 cells	NIH HIV Reagent Program	ARP-9849
CD4^+^ eGFP^-^ Sup-T1 cells	ATCC	CRL-1942
CD4^-^ eGFP^-^ Sup-T1 cells	This study	N/A
CD4^+^ eGFP^+^ Sup-T1 cells	This study	N/A
CD4^-^ eGFP^+^ Sup-T1 cells	This study	N/A
**Experimental models: Organisms/strains**		
Mouse: NSG-SGM3	The Jackson Laboratory	JAX: 013062
**Experimental models: Bacterial and viral strains**		
NEB® 5-alpha cells	New England Biolabs	C2987
NEB® 10-beta cells	New England Biolabs	C3019
pYK-JR-CSF	NIH HIV Reagent Program	ARP-2708
pNL4-3	NIH HIV Reagent Program	ARP-114
pNLENG1-IRES	(Levy et al, 2004)	N/A
**Recombinant DNA**		
pMLV Gag-Pol	(Mangeot et al, 2019)	N/A
pBLADE	(Mangeot et al, 2019)	Addgene plasmid #134912
pMLV Gag-mCherry	(Mangeot et al, 2019)	N/A
pMLV Gag-3xNES-Cas9	Addgene	Addgene plasmid #181752
pMV Hmut-3F11	This study	N/A
pVSV Gmut	This study	N/A
pCMV-VSV G	Addgene	Addgene plasmid #8454
pSpCas9-2A-Puro	Addgene	Addgene plasmid #62988
**Antibodies**		
Mouse monoclonal anti-Cas9 (clone 10C11-A12)	Thermo Fisher Scientific	Cat# MA1-202, RRID: AB_2633310
Mouse monoclonal anti-MLV p30 (clone 4B2)	Abcam	Cat# ab130757, RRID: AB_2895874
Goat polyclonal anti-mouse IgG (H + L), HRP	Thermo Fisher Scientific	Cat# 31430, RRID:AB_228307
Mouse monoclonal anti-His-tag, HRP	BioLegend	Cat# 652503, RRID: AB_2734520
Rabbit polyclonal anti-VSV-G tag, HRP	Abcam	Cat# ab3556, RRID: AB_303903
Human TruStain FcX™	BioLegend	Cat# 422302, RRID: AB_2818986
Rat monoclonal anti-mouse CD16/CD32 (clone 93)	Thermo Fisher Scientific	Cat# 14-0161-82, RRID: AB_467133
Rat monoclonal anti-mouse CD45, PE-Cy7 (clone 30-F11)	BioLegend	Cat# 103114, RRID: AB_312979
Mouse monoclonal anti-human CD3, PE-Cy5.5 (clone 7D6)	Thermo Fisher Scientific	Cat# MHCD0318, RRID: AB_10376001
Mouse monoclonal anti-human CD16, PE-TexasRed (clone 3G8)	Thermo Fisher Scientific	Cat# MHCD1617, RRID: AB_10373685
Mouse monoclonal anti-human CD4, PE (clone RPA-T4)	BD Biosciences	Cat# 555347, RRID: AB_395752
Mouse monoclonal anti-human CD8, FITC (G42-8)	BD Biosciences	Cat# 551347, RRID: AB_394159
Mouse monoclonal anti-human CD56, APC-Cy7 (clone HCD56)	BioLegend	Cat# 318332, RRID: AB_10896424
Mouse monoclonal anti-human CD14, Alexa Fluor 700 (clone TuK4)	Thermo Fisher Scientific	Cat# MHCD1429, RRID: AB_10373535
Mouse monoclonal anti-human CD11c, APC (clone B-ly6)	BD Biosciences	Cat# 559877, RRID: AB_398680
Mouse monoclonal anti-human CD19, BV605 (clone SJ25C1)	BD Biosciences	Cat# 562653, RRID: AB_2722592
Mouse monoclonal anti-human CD45, Pacific Blue (clone HI30)	BioLegend	Cat# 304022, RRID: AB_493655
LIVE/DEAD™ Fixable Aqua	Thermo Fisher Scientific	Cat# L34957
Mouse monoclonal anti-human CD16, PE-Cy7 (clone 3G8)	BioLegend	Cat# 302016, RRID: AB_314216
Mouse monoclonal anti-human CD4, BB515 (clone RPA-T4)	BD Biosciences	Cat# 564419, RRID: AB_2744419
Mouse monoclonal anti-human CD8, RY586 (clone RPA-T8)	BD Biosciences	Cat# 568114, RRID: AB_3684046
Mouse monoclonal anti-human HLA-DR + DP + DQ (clone CR3/43)	Abcam	Cat# ab7856, RRID: AB_306142
Mouse monoclonal anti-HIV Env (clone 3B3)	NIH HIV Reagent Program	Cat# ARP-12560
Goat polyclonal anti-Mouse, HRP	Immunologic	Cat# DPVM-HRP
Mouse monoclonal isotype control (clone B11/6)	Abcam	Cat# ab91353, RRID: AB_2811128
Mouse monoclonal anti-Cas9 (clone 10C11-A12)	Thermo Fisher Scientific	Cat# MA1-202, RRID: AB_2633310
**Oligonucleotides and other sequence-based reagents**		
Primers and probes for HIV integrase, murine GAPDH, murine RER129, murine TBP, human YWHAZ, see Table 1	IDT	N/A
Primers for RU5, Env, RPP30, see Table 1	IDT	N/A
Primers for CD4, eGFP, Tat, see Table 1	IDT	N/A
Duplex DNA oligos for cloning gRNA target sequences in pBLADE eGFP: 5’-CACCGGGCGAGGAGCTGTTCACCG-3’ and 5’-AAACCGGTGAACAGCTCCTCGCCC-3’ CD4: 5’-CACCGGCAAGGCCACAATGAACCG-3’ and 5’-AAACCGGTTCATTGTGGCCTTGCC-3’ Scrambled: 5’-CACCGAAGCGCGTTGCCGAGTGGC-3’ and 5’-AAACGCCACTCGGCAACGCGCTTC-3’ HIV Tat 1: 5’-CACCGTCTCCTATGGCAGGAAGAAG-3’ and 5’-AAACCTTCTTCCTGCCATAGGAGAC-3’ HIV Tat 2: 5’-CACCGAAGGAATCGAAGAAGAAGG-3’ and 5’-AAACCCTTCTTCTTCGATTCCTTC-3’	IDT	N/A
Single guide RNA for RNP formation CD4: mG*mG*mC* rArArG rGrCrC rArCrA rArUrG rArArC rCrGrG rUrUrU rUrArG rArGrC rUrArG rArArA rUrArG rCrArA rGrUrU rArArA rArUrA rArGrG rCrUrA rGrUrC rCrGrU rUrArU rCrArA rCrUrU rGrArA rArArA rGrUrG rGrCrA rCrCrG rArGrU rCrGrG rUrGrC mU*mU*mU* rU Scrambled: mG*mA*mA* rGrCrG rCrGrU rUrGrC rCrGrA rGrUrG rGrCrG rUrUrU rUrArG rArGrC rUrArG rArArA rUrArG rCrArA rGrUrU rArArA rArUrA rArGrG rCrUrA rGrUrC rCrGrU rUrArU rCrArA rCrUrU rGrArA rArArA rGrUrG rGrCrA rCrCrG rArGrU rCrGrG rUrGrC mU*mU*mU* rU	IDT	N/A
**Chemicals, enzymes and other reagents**		
Fetal calf serum	Biowest	S1400
DMEM	Thermo Fisher Scientific	41965039
RPMI	Thermo Fisher Scientific	61870010
Opti-MEM™	Thermo Fisher Scientific	31985070
L-Glutamine	Thermo Fisher Scientific	25030024
Penicillin/Streptomycin	Thermo Fisher Scientific	15140122
Lymphoprep	StemCell Technologies	07811
PBS pH 7.2	Thermo Fisher Scientific	20012019
EDTA	Thermo Fisher Scientific	15575020
Puromycin	Thermo Fisher Scientific	A1113803
Recombinant IL-2	Miltenyi Biotec	130-097-743
JetOPTIMUS® reagent	Sartorius	101000006
Chloroquine diphosphate salt	Sigma-Aldrich	C6628, CAS: 50-63-5
Millex-HV filter unit 0.45 µm pore size	Sigma-Aldrich	SLHVM33RS
Amicon® Ultra 15 Centrifugal Filters, 100 kDa MWCO	Sigma-Aldrich	UFC9100
2X Laemmli sample buffer	Bio-Rad	1610737EDU
2-mercaptoethanol	Bio-Rad	1610710
Nitrocellulose membrane	Bio-Rad	1620094
10X Tris-buffered saline	Bio-Rad	1706435
Tween 20	Bio-Rad	1706531
Blotting-Grade Blocker	Bio-Rad	1706404
SuperSignal™ Western Blot Enhancer	Thermo Fisher Scientific	46640
HEPES (1 M)	Thermo Fisher Scientific	15630080
Microvette® 200 K3 EDTA collection tubes	Sarstedt	20.1288.100
10X BD Pharm Lyse™ lysing buffer	BD Biosciences	555899
Polybrene	Sigma-Aldrich	TR-1003
Corning™ Cell strainers 50 µM	Thermo Fisher Scientific	Corning™ 431750
Propidium iodide	Miltenyi Biotec	130-093-233
T Cell TransAct™	Miltenyi Biotec	130-111-160
RetroNectin®	Takara Bio	T100A/B
autoMACS® Rinsing Solution	Miltenyi Biotec	130-091-222
MACS® BSA Stock Solution	Miltenyi Biotec	130-091-376
Phorbol 12-myristate 13-acetate (PMA)	Thermo Fisher Scientific	J63916.MCR
*S. pyogenes* Cas9	IDT	1081059
IDT duplex buffer	IDT	11-01-03-01
Lonza P3 buffer and supplement	Lonza	V4XP-3032
Triumeq®	ViiV Healthcare	N/A
Sucralose MediDrop®	Clear H2O	75-01-1001
MediGel® Hazelnut cups	Clear H2O	71-10-5022
Stainless Steel Beads, 5 mm	Qiagen	69989
RLT buffer	Qiagen	79216
ATL buffer	Qiagen	939011
Cell Extraction Buffer PTR	Abcam	ab193970
EDTA powder	Sigma-Aldrich	EDS, CAS: 60-00-4
DAB+	Agilent	K3467
Paraformaldehyde 32% aqueous solution	Electron Microscopy Sciences	15714-S
NucleoBond Xtra Midi kit	Macherey-Nagel	740410.50
Cas9 ELISA kit	Cell Biolabs	PRB-5079
mCherry ELISA kit	Abcam	ab221829
Q5® High-Fidelity DNA Polymerase	New England Biolabs	M0491
CD4 MicroBeads, human	Miltenyi Biotec	130-097-048
CD34 MicroBead Kit	Miltenyi Biotec	130-046-702
DNeasy Blood & Tissue Kit	Qiagen	69504
RNeasy Mini Kit	Qiagen	74106
QIAamp Viral RNA Mini Kit	Qiagen	52904
Qubit™ Protein Assay Kit	Thermo Fisher Scientific	Q33211
qScript cDNA Synthesis Kit	Quantabio	95047
LightCycler® 480 Probes Master mix	Roche	04707494001
LightCycler® 480 SYBR Green I Master mix	Roche	04707516001
**Software**		
FlowJo™ Software	BD Biosciences	https://www.bdbiosciences.com/
IBM SPSS Software	IBM	https://www.ibm.com/
Image Lab Software	Bio-Rad	https://www.bio-rad.com/
Prism	GraphPad	https://www.graphpad.com/
Benchling	Benchling	https://www.benchling.com/
NDP.view2	Hamamatsu	https://www.hamamatsu.com/
LightCycler 480 v1.5.0.39	Roche	https://lifescience.roche.com
qbase+	Biogazelle	https://biogazelle-qbaseplus.software.informer.com/2.0/
Synthego Inference of CRISPR Edits analysis	Synthego	https://www.synthego.com/help/ice
**Other**		
96-well plates, flat bottom, TC-treated	VWR	734-2327
96-well plates, U bottom, TC-treated	VWR	734-2328
Six-well plates, flat bottom, TC-treated	VWR	734-2323
10 cm cell culture dish	VWR	734-2817

### Cells

HEK293T cells (ATCC #CRL-3216) and HeLa-derived TZM-bl cells (NIH HIV Reagent Program, #ARP-8129, contributed by John C. Kappes, Xiaoyun Wu and Tranzyme Inc.) were grown in DMEM (Thermo Fisher Scientific, Waltham, MA, USA) supplemented with 10% fetal calf serum (FCS; Biowest, Bradenton, FL, USA), 2 mM L-glutamine (Thermo Fisher Scientific), and 1% antibiotics (penicillin/streptomycin; Thermo Fisher Scientific) at 37 °C, 5% CO_2_.

CD4^+/−^ eGFP^+/−^ Sup-T1 cells were grown in complete RPMI medium (i.e., RPMI 1640 medium supplemented with 2 mM L-glutamine, 10% FCS, and 1% antibiotics). CD4 knockout Sup-T1 cells (CD4^-^ eGFP^-^ Sup-T1 cells) were derived from Sup-T1 cells (ATCC #CRL-1942) using CRISPR-Cas9 gene editing. Briefly, 10^6^ parental Sup-T1 cells were electroporated for 10 mS at 1325 V with 10 µg SpCas9-2A-Puro (CRISPR RNA sequence 5’-ggcaaggccacaatgaaccg-3’) and subjected to puromycin selection (1 µg/mL, Thermo Fisher Scientific). A monoclonal cell line was generated via limiting dilution and flow cytometry (CD4 staining using BB515-labeled mouse anti-human CD4 antibodies [diluted 1:50, BD Biosciences, Franklin Lakes, NJ, USA, #564416]), as well as PCR amplification and Sanger sequencing of the CD4-encoding region (forward primer 5’-ctgagggagaggttgtgtatc-3’ and reverse primer 5’-gataaagattctgggaaatcaggg-3’ [Integrated DNA Technologies, San Diego, CA, USA], Q5 high-fidelity polymerase [New England Biolabs, Ipswich, MA, USA], cycling conditions: 30 s 98 °C, 35 cycles of 10 s 98 °C, 20 s 64 °C, 20 s 72 °C, and final elongation of 2 min 72 °C) were done to confirm *CD4* bi-allelic knock out. Lentiviral transduction of CD4^+/-^ Sup-T1 cells was performed to knock in the enhanced green fluorescent protein (*eGFP*) gene under the control of an EF1α promoter randomly into the genome of these cells. Fluorescence-activated cell sorting (FACS) was performed to generate polyclonal CD4^+/−^ eGFP^+^ Sup-T1 cells.

J-Lat 10.6 cells were obtained through the NIH HIV Reagent Program, Division of AIDS, NIAID, NIH (#ARP-9849, contributed by Dr. Eric Verdin) and maintained in complete RPMI medium.

All cell lines were regularly screened for mycoplasma contamination by PCR.

Human peripheral blood mononuclear cells (PBMCs) were isolated from Belgian Red Cross buffy coats by Lymphoprep™ (StemCell Technologies, Vancouver, BC, Canada) density gradient centrifugation. Donors included both male and female individuals aged between 18 and 60 years. Primary CD4^+^ cells were isolated from PBMCs by positive magnetic selection using CD4 MicroBeads (Miltenyi Biotec, Bergisch Gladbach, Rhineland, Germany, #130-097-048). Primary PBMCs and CD4^+^ cells were cultured in complete RPMI supplemented with 1 µg/mL recombinant IL-2 (Miltenyi Biotec).

Human CD34^+^ hematopoietic stem cells (HSCs) were isolated from human umbilical cord blood via Ficoll density gradient centrifugation followed by positive magnetic-activated cell sorting separation using the human CD34 MicroBead Kit (Miltenyi, #130-046-702). Human cord blood was obtained from the Cord Blood Bank of Ghent University Hospital and used following the guidelines of the Medical Ethical Committee of Ghent University Hospital (BC-10607).

### Viruses

HIV_JR-CSF_, HIV_NL4.3_, and HIV_NLENG1-IRES_ were produced by transfecting HEK293T cells seeded in 10 cm dishes with 10 µg of the respective infectious molecular clones using JetOPTIMUS reagent (Sartorius, Göttingen, Germany) in presence of 25 µM chloroquine diphosphate (Sigma-Aldrich, St. Louis, MO, USA). Medium was replaced by Opti-MEM (Thermo Fisher Scientific) 4 h post transfection. Supernatants were collected at 48 h as well as 72 h post transfection, including a media change with fresh Opti-MEM at 48 h. HIV virion-containing supernatants of these two collection time points were pooled and clarified by a short centrifugation step (300×*g*, 7 min, 4 °C) followed by filtration through a 0.45-μm Millex-HV filter unit (Sigma-Aldrich). Virus particles were concentrated over Opti-MEM-prewashed Amicon® Ultra 15 Centrifugal Filters (100 kDa MWCO; Sigma-Aldrich) by centrifugation (2000×*g*, 15 min, 4 °C), aliquoted and stored at −80 °C. Virus stocks were titrated by endpoint dilution in the TZM-bl cell line and by measuring β-galactosidase activity. The 50% endpoint was calculated according to the method of Reed and Muench (Reed and Müench, 1938).

### Humanized mice

NOD.Cg-*Prkdc*^*scid*^*Il2rg*^*tm1Wjl*^ Tg(CMV-IL3,CSF2,KITLG)1Eav/MloySzJ (NSG-SGM3) mice were obtained from the Jackson Laboratories (#013062) and bred under specific pathogen-free conditions at the animal facility of the Faculty of Medicine and Health Sciences, Ghent University. Mice were kept in Isocage (N)™ systems (Techniplast, Buguggiate, VA, Italy) designed for housing mice under biohazard A3 conditions. Approval of breeding and of all animal experiments was given by the Animal Ethics Committees of Ghent University (ECD21-33K and ECD21-34). A total of 10^5^ CD34^+^ HSCs were transplanted by intrahepatic injection in 100 cGy-irradiated newborn NSG-SGM3 pups (equal number of males and females). Human hematopoietic chimerism was verified in the blood of 9–10-week-old mice by flow cytometry (see below). Mice with human immune cell engraftment at 20% or higher of human CD45^+^ cells over total CD45^+^ cells were selected for experiments.

### Plasmids

pMLV Gag-Pol, pBLADE (gRNA), and pMLV Gag-mCherry were gifts from Els Verhoeyen (Mangeot et al, 2019). pMLV Gag-3xNES-Cas9 was a gift from David Liu (Addgene plasmid #181752). pMV Hmut-3F11 and pVSV Gmut were constructed through HiFi-assembly of PCR-amplified fragments in an eukaryotic expression plasmid harboring the human cytomegalovirus early promoter (CMV), the rabbit beta-globin intron and polyadenylation signals (backbone pCMV-VSV G was a gift from Bob Weinberg, Addgene plasmid #8454). Gene fragments encoding the anti-CD4 nanobody clone 3F11 (Roobrouck et al, 2015) and MV Hmut (Kneissl et al, 2012) were purchased from Integrated DNA Technologies (IDT, Newark, NJ, USA). HIV-1 JR-CSF infectious molecular clone (pYK-JR-CSF) was obtained through the NIH HIV Reagent Program, Division of AIDS, NIAID, NIH (ARP-2708) and contributed by Irvin S. Y. Chen and Yoshio Koyanagi. HIV-1_NL4.3_ infectious molecular clone (pNL4-3) was obtained through the NIH HIV Reagent Program, Division of AIDS, NIAID, NIH (ARP-114) and contributed by Dr. M. Martin. pNLENG1-IRES was kindly provided by David N. Levy. This construct contains replication-competent HIV_NL__4.3_ DNA expressing eGFP under the control of the viral long terminal repeat (LTR), and nef translation is mediated by an internal ribosome entry sequence (IRES) (Levy et al, 2004).

CD4-NB plasmids and infectious molecular clones were propagated in NEB® 5-alpha cells and NEB® 10-beta cells (#C2987 and #C3019, New England Biolabs, Ipswich, MA, USA), respectively. Plasmids were purified with the NucleoBond Xtra Midi kit (#740410.50, Macherey-Nagel, Düren, Germany).

### CD4-directed nanoblade production and characterization

CD4-NBs were generated in HEK293T cells plated at 4 × 10^6^ cells per 10 cm plate 24 h before transfection with plasmids pMLV Gag-Pol (2.8 µg), pMLV Gag-3xNES-Cas9 (1.4 µg), pMV Hmut-3F11 (720 ng), pVSV Gmut (400 ng), and pBLADE(s) (total of 4.4 µg) using JetOPTIMUS reagent in the presence of 25 µM chloroquine diphosphate. Medium was replaced by Opti-MEM 4 h post transfection. CD4-NBs were harvested, purified and concentrated from cell supernatants as described above for virus production. The amount of Cas9 was quantified using the Cas9 ELISA kit (Cell Biolabs, San Diego, CA, USA, #PRB-5079) to standardize Cas9 doses per experiment.

Western blotting was performed to detect Cas9, MLV p30, MV Hmut-3F11-His-tagged protein, and VSV Gmut. To do so, CD4-NBs were lysed using 2X Laemmli sample buffer (Bio-Rad, Hercules, CA, USA) supplemented with 5% 2-mercaptoethanol (Bio-Rad). Lysates were separated by SDS-PAGE and transferred onto nitrocellulose membranes. Membranes were blocked in tris-buffered saline with 0.1% Tween® 20 (TBS T, Bio-Rad) and 5% milk (Bio-Rad) for 1 h at room temperature and incubated with SuperSignal™ Western Blot Enhancer (Thermo Fisher Scientific, #46640) prior to incubation overnight at 4 °C with primary antibodies against Cas9 (diluted 1:200, clone 10C11-A12, Thermo Fisher Scientific, #MA1-202) or MLV p30 (diluted 1:1000, clone 4B2, Abcam, Cambridge, UK, #ab130757) followed by incubation with HRP-conjugated goat anti-mouse IgG antibodies (diluted 1:10,000, Thermo Fisher Scientific, #31430) for 1 h at room temperature. Directly HRP-conjugated antibodies against the His-tag (diluted 1:1000, clone J099B12, BioLegend, San Diego, CA, USA, #652503) or the VSV G-tag (diluted 1:10,000, Abcam, #ab3556) were incubated for 1 h at room temperature. Protein bands were visualized using chemiluminescent substrate.

Dynamic light scattering (DLS) and electrophoretic light scattering (ELS) were measured on a Zetasizer Nano ZS (Malvern Instruments Ltd, Malvern, UK) equipped with a HeNe laser (λ = 633 nm), and detection was done at a scattering angle of 173°. The samples were diluted 10X in 5 mM HEPES buffer (pH 7.4, Thermo Fisher Scientific) and measured in triplicate. Size (z-average) and charge (zeta potential) of CD4-NB particles were determined through the Stokes–Einstein equation and Smoluchowski equation, respectively.

Assessments were not performed under blinded conditions.

### gRNA design and sequences

gRNAs targeting *eGFP* and human *CD4*, and a scrambled sequence were designed using the Benchling CRISPR design tool (https://benchling.com). gRNAs targeting HIV *tat* have been described before (Herskovitz et al, 2021). The CRISPR RNA sequences are provided below:

*eGFP*: 5’ GGGCGAGGAGCTGTTCACCG 3’

Human *CD4*: 5’ GGCAAGGCCACAATGAACCG 3’

Scrambled: 5’ GAAGCGCGTTGCCGAGTGGC 3’

HIV *tat 1*: 5’ TCTCCTATGGCAGGAAGAAG 3’

HIV *tat 2*: 5’ GAAGGAATCGAAGAAGAAGG 3’

### Immunofluorescent staining and flow cytometry

To verify human immune cell engraftment in NSG-SGM3 mice, 200 µL blood was collected in Microvette® 200 K3 EDTA collection tubes (Sarstedt, Nümbrecht, Germany). Erythrocytes were lysed using BD Pharm Lyse™ lysing buffer (BD Biosciences). Bone marrow was flushed with PBS from the femurs and tibias of humanized mice. Cells were pre-incubated with Human TruStain FcX™ diluted 1:20 (BioLegend, #422302) and CD16/CD32 monoclonal antibodies diluted 1:100 (Thermo Fisher Scientific, #14-0161-82) in PBS for 30 min at 4 °C to block human and murine Fc receptors. Next, the following antibodies and cell viability tracker diluted in PBS were added to the cells for 1 h at 4 °C: muCD45 PE-Cy7 diluted 1:100 (clone 30-F11, BioLegend, San Diego, CA, USA, #103114), huCD3 PE-Cy5.5 diluted 1:200 (clone 7D6, Thermo Fisher Scientific, #MHCD0318), huCD16 PE-TexasRed diluted 1:100 (clone 3G8, Thermo Fisher Scientific, #MHCD1617), huCD4 PE diluted 1:50 (clone RPA-T4, BD Biosciences, Franklin Lakes, NJ, USA, #555347), huCD8 FITC diluted 1:100 (clone G42-8, BD Biosciences, #551347), huCD56 APC-Cy7 diluted 1:100 (clone HCD56, BioLegend, #318332), huCD14 Alexa Fluor 700 diluted 1:200 (clone TuK4, Thermo Fisher Scientific, #MHCD1429), huCD11c APC diluted 1:100 (clone B-ly6, BD Biosciences, #559877), huCD19 BV605 diluted 1:50 (clone SJ25C1, BD Biosciences, #562653), huCD45 Pacific Blue diluted 1:50 (clone HI30, BioLegend, #304022), LIVE/DEAD™ Fixable Aqua diluted 1:1000 (Thermo Fisher Scientific, #L34957). After two washing steps in PBS, samples were analyzed on a LSRFortessa™ Cell Analyzer (BD Biosciences).

To detect mCherry signal of CD4-directed kirsch nanoblades (CD4 K-NBs) on specific human cell subsets, humanized mouse-derived leukocytes were pretreated as described above and primary human PBMCs were pre-incubated only with Human TruStain FcX™ (BioLegend, #422302). The above-described staining panel was slightly adjusted by removing muCD45 PE-Cy7 and/or huCD45 Pacific Blue and changing to the following antibodies: huCD16 PE-Cy7 diluted 1:100 (clone 3G8, BioLegend, #302016), huCD4 BB515 diluted 1:50 (clone RPA-T4, BD Biosciences, #564419), huCD8 RY586 diluted 1:200 (clone RPA-T8, BD Biosciences, #568114). Samples were analyzed on a FACSymphony™ A5 device (BD Biosciences). Gating strategies for buffy coat-derived PBMCs and humanized mouse-derived PBMCs are shown in Figs. EV2 and EV3B, respectively.

### In vitro incubation of PBMCs with CD4-directed kirsch nanoblades

PBMCs were plated in 96-well flat-bottom plates (Avantor, Radnor, PA, USA) at a density of 1,000,000 cells per well in coRPMI containing a final concentration of 1 µg/mL recombinant IL-2 (Miltenyi Biotec). Randomization was not required as all conditions were tested on all donors. CD4 K-NBs were diluted in Opti-MEM and added directly to the cell culture media in each well for 1 h at 37 °C. Suspension cells were collected, and attached cells were dislodged from the bottom by adding 5 mM EDTA (Thermo Fisher Scientific) dissolved in PBS and incubating the plate on ice for 40 min. Cells were then immunofluorescently stained as described above. Analyses were not performed under blinded conditions.

### In vivo distribution of CD4-directed kirsch nanoblades

Humanized mice (humanization level >20%) were randomized to different groups and injected retro-orbitally (intravenously) with CD4 K-NBs diluted in opti-MEM (2 µg mCherry) or opti-MEM alone (control). Three hours later, mice were sacrificed to collect blood, kidney, liver, lung, spleen, and femurs. Two hundred µL blood was centrifuged at 500×*g* for 10 min at 4 °C. Plasma was transferred to separate Eppendorf tubes and, together with the remaining cell pellets, frozen at −80 °C. Two hundred µL of blood was prepared for immunofluorescent staining as described above. Bone marrow was flushed with PBS from both femurs and divided into two parts. One part was pelleted at 500×*g* for 10 min at 4 °C prior to freezing at −80 °C. The other part was prepared for immunofluorescent staining as described above. One third of the spleens were minced mechanically on 50-μm cell strainers (Thermo Fisher Scientific), after which the RBCs were lysed, and cells were immunofluorescently stained as described above for blood-derived PBMCs. Pieces of ~25 mg were cut from the liver, lungs, spleen, and kidney prior to snap-freezing in liquid nitrogen and further storage at −80 °C prior to protein extraction. Assessments were performed under blinded conditions by different investigators.

### Transduction of cells with CD4-directed nanoblades

CD4^+/−^ eGFP^+/−^ Sup-T1 cells were plated in 96-well flat-bottom plates at a density of 50,000 cells per well in coRPMI containing a final concentration of 5 µg/mL polybrene (Sigma-Aldrich, #TR-1003). CD4-NBs were diluted in Opti-MEM and added directly to the cell culture media in each well. Cells were spinoculated by centrifugation at 1000×*g* for 90 min at 32 °C and further incubated for 3 h at 37 °C, 5% CO_2_. CD4-NB media was removed by a centrifugal wash step at 500×*g* for 7 min at 4 °C, and the cells were cultured in coRPMI. Two days post transduction, cellular genomic DNA was isolated from cell pellets with the DNeasy Blood & Tissue Kit (Qiagen, Venlo, The Netherlands, #69504). Expression of eGFP was analyzed five days post transduction by flow cytometry on a MACSQuant® Analyzer 10 (Miltenyi Biotec). Propidium iodide (Miltenyi Biotec, #130-093-233) was included as a cell viability marker.

Primary human CD4^+^ cells were transduced and analyzed in a similar manner, but the medium was supplemented with 1 µg/mL recombinant IL-2 and cells were activated using 0.5 µL human T Cell TransAct™ (Miltenyi Biotec, #130-111-160) per well after transduction. RetroNectin® (Takara Bio Inc., Kusatsu, Japan)-precoated plates were used during CD4-NB transduction of primary CD4^+^ cells. Randomization was not required as all conditions were tested on all donors. Expression of CD4 was analyzed (not blinded) five days post transduction via flow cytometry following immunofluorescent staining with anti-huCD4 BB515-labeled antibodies diluted 1:50 (clone RPA-T4, BD Biosciences, #564419) in MACS® buffer (i.e., autoMACS® Rinsing Solution containing MACS® BSA Stock Solution [diluted 1:20, Miltenyi Biotec, #130-091-222 and #130-091-376]) for 20 min at 4 °C.

J.Lat 10.6 cells were transduced as described for Sup-T1 cells. The next day, half of the cells were used to extract cellular genomic DNA. The other half was treated with 50 nM phorbol 12-myristate 13-acetate (PMA, Thermo Fisher Scientific) for 72 h prior to analysis by flow cytometry as described for Sup-T1 cells.

### Electroporation with sgRNA-Cas9 ribonucleoproteins

*S. pyogenes* Cas9 and single guide RNAs (sgRNAs) were purchased from IDT (#1081059). Ten µg Cas9 was mixed with sgRNAs diluted to 30 µM in IDT duplex buffer (IDT, #11-01-03-01) for 30 min at room temperature to allow RNP formation. RNP complexes were electroporated in 10^6^ primary CD4^+^ cells resuspended in 20 μl of P3 buffer and supplement (Lonza V4XP-3032) in a 4D nucleofector (Lonza, Basel, Switzerland) using the EH-115 pulse code. Cells were incubated for 10 min at 37 °C, then rescued with 100 µl of pre-warmed antibiotic-free coRPMI medium before diluting in coRPMI containing 1 µg/mL recombinant IL-2 for further cell culture.

### In vitro HIV infection

CD4^+^ eGFP^-^ Sup-T1 cells and primary human CD4^+^ cells were inoculated with HIV_NLENG1-IRES_ in coRPMI containing 5 µg/mL polybrene at an MOI of 0.01 and 0.2, respectively. Cells were spinoculated as described above and further incubated for 3 h at 37 °C, 5% CO_2_. HIV inoculum was removed by a centrifugal wash step at 500×*g*, 7 min, 4 °C and cells were cultured in coRPMI overnight prior to CD4-NB transduction. Primary human CD4^+^ cells were cultured in coRPMI containing 1 µg/mL recombinant IL-2.

### In vivo HIV infection, antiretroviral treatment, and CD4-directed nanoblade treatment

Humanized mice (humanization level >20%) were infected with 10^5^ TCID_50_ of HIV_JR-CSF_ by intraperitoneal injection. Two weeks post inoculation, blood was collected by retro-orbital bleeding and plasma viremia levels were verified by RT-qPCR (see below). Next, cART was initiated and continued for 14 days to stop ongoing virus replication prior to CD4-NB treatment using a single tablet regimen of dolutegravir/abacavir/lamivudine (Triumeq®, ViiV Healthcare, London, UK). Tablets were crushed and dissolved in Sucralose MediDrop® (Clear H2O, Westbrook, ME, USA) and subsequently mixed in MediGel® Hazelnut cups (Clear H2O) at the therapeutic concentration of 24 mg per cup. Cups were replaced every other day. After 14 days of cART treatment, mice were randomized to two different groups and CD4-NBs (300 ng Cas9) with either scrambled sgRNAs or sgRNAs against HIV *tat* were injected intravenously as well as intraperitoneally. In the second experiment, mice received a second dose of CD4-NBs two days later. Blood was collected once per week by retro-orbital bleeding for three weeks. At the end of the experiment, mice were sacrificed to collect blood, lungs, spleen, femurs, and tibias.

### Tissue homogenization

Tissue homogenates (lungs, spleen, liver, and kidney) were prepared using bead mill technology by high-speed shaking (50 Hz) of tissues with 5 mm stainless steel beads (Qiagen) for 5 min in a TissueLyser LT (Qiagen). Ten percent (w/v) solutions of tissues were made in RLT buffer (Qiagen, Hilden, Germany, #79216) supplemented with 1% 2-mercaptoethanol (Bio-Rad) prior to RNA extraction, ATL buffer (Qiagen, #939011) prior to DNA extraction, or cell extraction buffer PTR (Abcam, #ab193970) for protein extraction. Tissue homogenates were then clarified by centrifugation at 13,000 × *g* for 10 min at 4 °C.

### Protein extraction, quantification, and mCherry ELISA

Cell pellet and tissue lysates were prepared in cell extraction buffer PTR (Abcam, #ab193970) and protein concentrations were measured using a Qubit™ Protein Assay Kit (Thermo Fisher Scientific, #Q33211) according to the manufacturer’s instructions. mCherry concentrations were determined using an mCherry ELISA kit (Abcam, #ab221829).

### Nucleic acid extraction and cDNA synthesis

The DNeasy Blood & Tissue kit (#69504, Qiagen) and the RNeasy Mini Kit (#74106, Qiagen) were used according to the manufacturer’s instructions to isolate DNA and RNA, respectively, from cells and clarified tissue homogenates. Viral RNA was extracted from plasma using the QIAamp Viral RNA Mini Kit (#52904, Qiagen) according to the manufacturer’s instructions. A total of 1 µg (cells and tissues) or 12 µL (plasma) RNA was reverse transcribed to cDNA using the qScript cDNA Synthesis Kit (Quantabio, Beverly, MA, USA, #95047) following the manufacturer’s instructions.

### qPCR analysis

HIV integrase qPCR: Two microliters of cDNA was subjected in duplicate to quantitative PCR (qPCR) using a qPCR probe assay (Table 1) with LightCycler® 480 Probes Master mix (#04707494001, Roche). For plasma samples, a tenfold dilution series of pYK-JR-CSF plasmid control, corresponding to a range of 10^1.8^ to 10^6.8^ HIV copies, functioned as a standard. qPCR was performed with a Light-Cycler® 480 Real-Time PCR System (Roche) with the following amplification conditions: preincubation at 95° for 5 min with 45 cycles of denaturation (10 s at 95 °C), annealing (20 s at 58 °C) and elongation (30 s at 72 °C) followed by a melting curve from 65 to 95 °C. LightCycler 480 v1.5.0.39 was used for data collection. For plasma samples, sample CT values were plotted against standard dilution values to determine the exact HIV RNA copies.

**Table 1:** Primers and probes used for (q)PCR analysis.

Target	Forward primer (5’ to 3’)	Reverse primer (5’ to 3’)	Probe (5’-3’)
HIV integrase	GGTTTATTACAGGGACAGCAGAGA	ACCTGCCATCTGTTTTCCATA	FAM-ACTACTGCCCCTTCACCTTTCCARAG-BHQ1
Murine GAPDH	GTGATGGGTGTGAACCACGAGAA	ACTGTGGTCATGAGCCCTTCC	/
Murine RER129	AGTGGATCCTTCCTTGATGG	ATGCCTTTTGTAGCTGCG	/
Murine TBP	CGGCATCAGATGTGCGTCA	CAGAAACCTAGCCAAACCGC	/
Human YWHAZ	ACTTTTGGTACATTGTGGCTTCAA	CCGCCAGGACAAACCAGTAT	/
HIV RU5	TTAAGCCTCAATAAAGCTTGCC	GTTCGGGCGCCACTGCTAGA	/56ROXN/CCAGAGTCACACAACAGACGGGCACA/3IAbRQSp/
HIV env	AGTGGTGCAGAGAGAAAAAAGAGC	GTCTGGCCTGTACCGTCAGC	/5HEX/CCTTGGGTT/ZEN/CTTGGGA/3IABkFQ/
Human RPP30 1	GCTAACCTTTGATAGGCTCAGT	CAAATGGTAAGAAGGGCAGAAAC	56FAM/AGCTAAGTG/ZEN/TTGATCCATCTCTTTCTAGCT/3IABkFQ/
Human RPP30 2	AGATTTGGACCTGCGAGCG	GAGCGGCTGTCTCCACAAGT	5Cy5/TTCTGACCT/TAO/GAAGGCTCTGCGCG/3IAbRQSp/
Human CD4	CTGAGGGAGAGGTTGTGTATC	CCCTGATTTCCCAGAATCTTTATC	/
eGFP	CGAGGATTGTGGAACTTCTGG	TGTGGCTGTTGTAGTTGTACTC	/
HIV tat 1-2	AAGCCACCTTTGCCTAGTGT	CCAGAAGTTCCACAATCCTCG	/
HIV tat 1-2 nested	AGATGGAACAAGCCCCAGAAGA	CAAGAGTAAGTCTCTCAAGCGGTGGTA	/

Housekeeping genes qPCR: Two microliters of cDNA, corresponding to 25 ng RNA, was subjected in duplicate to qPCR using LightCycler® 480 SYBR Green I Master mix (#04707516001, Roche) and specific primers for murine GAPDH, murine RER129, murine TBP, and human YWHAZ (Table 1). No template sample was used as a negative control. The qPCR was performed on a LightCycler® 480 Real-Time PCR System (Roche) with the following amplification conditions: preincubation at 95° for 2 min with 45 cycles of denaturation (15 s at 95 °C), annealing (30 s at 60 °C), and extension (15 s at 72 °C), followed by a melting curve from 55 to 95 °C. LightCycler 480 v1.5.0.39 was used for data collection. Relative gene expression was calculated using qbase+ software v3.2 (Biogazelle, Zwijnaarde, Belgium).

Analyses were done in a blinded manner by different investigators.

### dPCR analysis

The levels of RU5 and env HIV-1 DNA were quantified using a multiplex digital PCR assay similar to the Rainbow proviral HIV-1 DNA assay (Delporte et al, 2025). Adaptations included the replacement of the psi and gag targets with two RPP30 targets for cellular input quantification, allowing immediate normalization within one reaction. All sequences were ordered and synthesized by IDT. Digital PCR reactions were prepared with four primer/probe sets (Table 1) at different concentrations, 10 µL 4X concentrated QIAcuity Probe Master Mix (ID: 250102), 15 µL template DNA at 75 ng/µL where possible and nuclease-free water up to 40 µL total volume per reaction. The dPCR reaction mixes were transferred into a 26 K 24-well nanoplate (ID: 250001, Qiagen) and loaded into the QIAcuity Four dPCR platform for partitioning, PCR cycling and imaging. The digital PCR program started with 2 min at 95 °C for the activation of the Qiacuity probe mix, followed by 40 cycles of 94 °C for 30 s and 56 °C for 60 s. Default imaging settings for exposure times were used for all fluorescent channels (green: 500 ms, yellow: 500 ms, red: 200 ms, crimson: 400 ms). All samples were analyzed in duplicate. The QIAcuity Software Suite (version 3.2.0.0) was used to analyse the data. Thresholds were set above the partitions observed in no template controls and applied to the samples on the same nanoplate. Copy numbers were calculated using Poisson statistics implemented by the software. The cellular input per microliter reaction was determined by averaging the copy numbers of the two RPP30 targets and dividing by two, reflecting diploid human genomes. HIV-1 DNA levels of RU5 and env were then normalized to this cellular input and reported as copies per million human cells. Analyses were done in a blinded manner by different investigators.

### PCR amplification, Sanger and Oxford Nanopore Technologies (ONT) sequencing

Conventional PCR was performed using a thermal cycler on 150 ng template DNA in the presence of 12.5 µL Q5 high-fidelity polymerase mix (NEB, #M0491), 0.5 µM forward and reverse primers (Table 1) and water, bringing the total volume to 25 µL. The following cycling conditions were used for (i) *CD4*: 30 s 98 °C; 35 cycles of 10 s 98 °C, 20 s 64 °C, 20 s 72 °C; and 2 min 72 °C, and (ii) *eGFP*: 30 s 98 °C; 35 cycles of 10 s 98 °C, 20 s 65 °C, 60 s 72 °C; and 2 min 72 °C. These PCR amplicons were purified, Sanger sequenced (Eurofins Scientific, Luxembourg, Luxembourg) and subjected to tracking INDELs by Synthego Inference of CRISPR Edits or Tracking of Indels by DEcomposition (TIDE) analysis (Nakamae and Bono, 2022). PCR amplicons of primary CD4^+^ T cells were additionally sequenced via Oxford Nanopore Technologies (ONT) sequencing. Reads were mapped to patient-specific allele reference sequences built from the control samples. When both alleles of a patient are identical, a single reference sequence was used. Mutations frequencies were analysed around 10 bp of the gRNA cut site of *CD4* for both alleles (PathoSense, Gent, Belgium). Analyses were not blinded.

CRISPR-mediated excision of proviral DNA was determined by gel electrophoresis of nested PCR amplicons (Herskovitz et al, 2021) as well as ONT sequencing (PathoSense). Briefly, 150 ng template DNA was mixed with 12.5 µL Q5 high-fidelity polymerase mix (#M0491, NEB), 0.5 µM forward and reverse primers and water, bringing the total volume to 25 µL. Conventional PCR was performed on a thermal cycler: 30 s 98 °C; 15 cycles of 10 s 98 °C, 20 s 66 °C, 60 s 72 °C; 2 min 72 °C. This PCR product was then diluted 1:10 in water, and 2 µL hereof functioned as template DNA for 30 additional rounds of the same conventional PCR thermal cycle conditions and setup, but with nested-specific primers. The final PCR product was subjected to gel electrophoresis or sent for ONT-based sequencing to determine the read abundance of the excised 544 bp fragment compared to the intact 2989 bp fragment (PathoSense). In addition, INDELs around the tat 1 gRNA cut site were tracked using the Synthego Inference of CRISPR Edits analysis or TIDE tools on Sanger sequencing data (Eurofins) obtained from the gel-purified 2989 bp fragment. Analyses were not blinded.

### Immunohistochemistry

Spleens and femur bones were fixed for 24 h in 1% paraformaldehyde (Sigma-Aldrich) diluted in PBS. Bone marrow was additionally decalcified for 3 days in 14% EDTA dissolved in PBS (Sigma-Aldrich). Following a 2 h washing step in PBS, all tissues were embedded in paraffin. Three µm thick sections were cut and mounted on glass slides. Slides were deparaffinized and stained using mouse monoclonal antibodies for HLA DQ + DP + DR (clone CR3/43, diluted 1:100, #ab7856, Abcam) and HIV-1 env (Clone 3B3, 1:50, #ARP-12560 obtained through the NIH Aids Reagent Program Division of AIDS, NIAID and contributed by Duke Human Vaccine Institute, Duke University Medical Center) following heat-mediated antigen retrieval for 45 min at 95 °C in citrate buffer pH 6 (human HLA) or Tris/EDTA buffer pH 9 (HIV env). Next, horseradish peroxidase (HRP)-labeled poly anti-mouse IgG antibodies (undiluted, # DPVM-HRP, Immunologic, Duiven, The Netherlands) were added. Immunostaining was visualized using DAB+ (Agilent, Santa Clara, CA, USA, #K3467) and hematoxylin II was used for counterstaining. Mouse IgG (Abcam, #ab91353) served as a negative control. All slides were digitally scanned using the Hamamatsu NanoZoomer 2.0RS and analyzed using Hamamatsu NDP.view v2.9.25.

Analyses were done in a blinded manner by different investigators.

### Quantification and statistical analysis

Significant differences (*P* < 0.05) among cell subsets, treatment types (e.g., various CD4-NBs, electroporation, mock), or Cas9 doses were determined using analysis of variance (ANOVA), followed by either a Tukey post hoc test (for comparisons among all groups) or a Dunnett post hoc test (for comparisons against a control group). When multiple donors were included, donor identity was treated as a blocking factor. In case interactions between multiple factors were identified, cases were split by the latter factor prior to ANOVA. If homoscedasticity of the variables was not met, as assessed by Levene’s test, the data were log-transformed prior to ANOVA. The normality of the residuals was verified using the Shapiro–Wilk test. If the variables remained heteroscedastic or normality was not met after log transformation, a Kruskal–Wallis test followed by a Mann–Whitney post hoc test were performed. Differences in plasma viremia levels between scrambled and tat 1-2 CD4-NBs across time points were analyzed using a repeated measures model. All analyses were conducted in IBM SPSS Statistics for Windows, version 29.0 (IBM, Armonk, NY, USA). No data were omitted from analyses except when one technical replicate was lost (Fig. 5B, Sup-T1 cells experiment 3 and D2 treated with eGFP CD4-NBs) or an organ was lost during processing (bone marrow exp 2 from one mice treated with tat 1-2 CD4-NBs).

## Supplementary information

Appendix

Peer Review File

Source data Fig. 1

Source data Fig. 2

Source data Fig. 3

Source data Fig. 4

Source data Fig. 5

Source data Fig. 6

Expanded View Figures

## Data Availability

This study includes no data deposited in external repositories. The source data of this paper are collected in the following database record: biostudies:S-SCDT-10_1038-S44321-026-00481-x.
