# Supplementary material for: CD4-directed nanoblades enable selective genome editing in CD4+ cells and HIV suppression in vitro and in vivo
Source: EMBO Mol Med. 2026 Jul 6;18(8):3345–73. doi: 10.1038/s44321-026-00481-x (PMC13470122; doi:10.1038/s44321-026-00481-x)
Supplement: Supplementary file 1 [file 44321_2026_481_MOESM1_ESM.pdf]

Appendix

Table of contents

Table of contents 1

Appendix table S1: exact p values for blood huCD45$^+$ cells of Figure 2E 2

Appendix table S2: exact p values for splenic huCD45$^+$ cells of Figure 2E 3

Appendix table S3: exact p values for bone marrow huCD45$^+$ cells of Figure 2E 5

Appendix table S4: exact p values for batch comparison in Figure EV3 E 6

Appendix table S1: exact p values for blood huCD45^{+} cells of Figure 2E

Cell_type 1	Cell_type 2	p value
B cells	CD8 T cells	,996
	CD4 T cells	,002
	NK cells	1,000
	CD4+ monocytes	<,001
	CD4- monocytes	,965
	CD4+ dendritic cells	<,001
	CD4- dendritic cells	,988
CD8 T cells	B cells	,996
	CD4 T cells	<,001
	NK cells	1,000
	CD4+ monocytes	<,001
	CD4- monocytes	,642
	CD4+ dendritic cells	<,001
	CD4- dendritic cells	,749
CD4 T cells	B cells	,002
	CD8 T cells	<,001
	NK cells	<,001
	CD4+ monocytes	,219
	CD4- monocytes	,035
	CD4+ dendritic cells	,009
	CD4- dendritic cells	,022
NK cells	B cells	1,000
	CD8 T cells	1,000
	CD4 T cells	<,001
	CD4+ monocytes	<,001
	CD4- monocytes	,818
	CD4+ dendritic cells	<,001
	CD4- dendritic cells	,894
CD4+ monocytes	B cells	<,001
	CD8 T cells	<,001
	CD4 T cells	,219
	NK cells	<,001
	CD4- monocytes	<,001
	CD4+ dendritic cells	,889

	CD4- dendritic cells	<,001
CD4- monocytes	B cells	,965
	CD8 T cells	,642
	CD4 T cells	,035
	NK cells	,818
	CD4+ monocytes	<,001
	CD4+ dendritic cells	<,001
	CD4- dendritic cells	1,000
CD4+ dendritic cells	B cells	<,001
	CD8 T cells	<,001
	CD4 T cells	,009
	NK cells	<,001
	CD4+ monocytes	,889
	CD4- monocytes	<,001
	CD4- dendritic cells	<,001
CD4- dendritic cells	B cells	,988
	CD8 T cells	,749
	CD4 T cells	,022
	NK cells	,894
	CD4+ monocytes	<,001
	CD4- monocytes	1,000
	CD4+ dendritic cells	<,001

Appendix table S2: exact p values for splenic $huCD45^+$ cells of Figure 2E

Cell_type 1	Cell_type 2	p value
B cells	CD8 T cells	,564
	CD4 T cells	1,000
	NK cells	1,000
	CD4+ monocytes	,988
	CD4- monocytes	,013
	CD4+ dendritic cells	,677
	CD4- dendritic cells	,003
CD8 T cells	B cells	,564
	CD4 T cells	,615
	NK cells	,473
	CD4+ monocytes	,144

	CD4- monocytes	<,001
	CD4+ dendritic cells	,020
	CD4- dendritic cells	<,001
CD4 T cells	B cells	1,000
	CD8 T cells	,615
	NK cells	1,000
	CD4+ monocytes	,980
	CD4- monocytes	,011
	CD4+ dendritic cells	,627
	CD4- dendritic cells	,003
NK cells	B cells	1,000
	CD8 T cells	,473
	CD4 T cells	1,000
	CD4+ monocytes	,996
	CD4- monocytes	,019
	CD4+ dendritic cells	,763
	CD4- dendritic cells	,005
CD4+ monocytes	B cells	,988
	CD8 T cells	,144
	CD4 T cells	,980
	NK cells	,996
	CD4- monocytes	,101
	CD4+ dendritic cells	,988
	CD4- dendritic cells	,032
CD4- monocytes	B cells	,013
	CD8 T cells	<,001
	CD4 T cells	,011
	NK cells	,019
	CD4+ monocytes	,101
	CD4+ dendritic cells	,458
	CD4- dendritic cells	1,000
CD4+ dendritic cells	B cells	,677
	CD8 T cells	,020
	CD4 T cells	,627
	NK cells	,763
	CD4+ monocytes	,988
	CD4- monocytes	,458
	CD4- dendritic cells	,203

CD4- dendritic cells	B cells	,003
	CD8 T cells	<,001
	CD4 T cells	,003
	NK cells	,005
	CD4+ monocytes	,032
	CD4- monocytes	1,000
	CD4+ dendritic cells	,203

Appendix table S3: exact p values for bone marrow $huCD45^{+}$ cells of Figure 2E

Cell_type 1	Cell_type 2	p value
B cells	CD8 T cells	,001
	CD4 T cells	1,000
	NK cells	1,000
	CD4+ monocytes	,132
	CD4- monocytes	1,000
	CD4+ dendritic cells	,359
	CD4- dendritic cells	1,000
CD8 T cells	B cells	,001
	CD4 T cells	,387
	NK cells	,069
	CD4+ monocytes	1,000
	CD4- monocytes	1,000
	CD4+ dendritic cells	1,000
	CD4- dendritic cells	,020
CD4 T cells	B cells	1,000
	CD8 T cells	,387
	NK cells	1,000
	CD4+ monocytes	1,000
	CD4- monocytes	1,000
	CD4+ dendritic cells	1,000
	CD4- dendritic cells	1,000
NK cells	B cells	1,000
	CD8 T cells	,069
	CD4 T cells	1,000
	CD4+ monocytes	1,000
	CD4- monocytes	1,000

	CD4+ dendritic cells	1,000
	CD4- dendritic cells	1,000
CD4+ monocytes	B cells	,132
	CD8 T cells	1,000
	CD4 T cells	1,000
	NK cells	1,000
	CD4- monocytes	1,000
	CD4+ dendritic cells	1,000
	CD4- dendritic cells	1,000
CD4- monocytes	B cells	1,000
	CD8 T cells	1,000
	CD4 T cells	1,000
	NK cells	1,000
	CD4+ monocytes	1,000
	CD4+ dendritic cells	1,000
	CD4- dendritic cells	1,000
CD4+ dendritic cells	B cells	,359
	CD8 T cells	1,000
	CD4 T cells	1,000
	NK cells	1,000
	CD4+ monocytes	1,000
	CD4- monocytes	1,000
	CD4- dendritic cells	1,000
CD4- dendritic cells	B cells	1,000
	CD8 T cells	,020
	CD4 T cells	1,000
	NK cells	1,000
	CD4+ monocytes	1,000
	CD4- monocytes	1,000
	CD4+ dendritic cells	1,000

Appendix table S4: exact p values for batch comparison in Figure EV3 E

Batch 1	Batch 2	p value
Batch 1	Batch 2	,970
	Batch 3	,351
	Control	,012

Batch 2	Batch 1	,970
	Batch 3	,333
	Control	,013
Batch 3	Batch 1	,351
	Batch 2	,333
	Control	,003
Control	Batch 1	,012
	Batch 2	,013
	Batch 3	,003
