# Supplementary material for: CD4-directed nanoblades enable selective genome editing in CD4+ cells and HIV suppression in vitro and in vivo
Source: EMBO Mol Med. 2026 Jul 6;18(8):3345–73. doi: 10.1038/s44321-026-00481-x (PMC13470122; doi:10.1038/s44321-026-00481-x)
Supplement: Supplementary file 2 [file 44321_2026_481_MOESM2_ESM.pdf]

EMBO Molecular Medicine

CD4-directed Nanoblades enable selective genome editing in CD4+ cells and HIV suppression in vitro and in vivo

Jolien Van Cleemput, Maaike De Cock, Rianne Verbeek, Elianne Burg, Bram Van den Eeckhout, Wojciech Witkowski, Willem van Snippenberg, Zoe Stylianidou, Emily Brugger Galetic, Mareva Delporte, Ken Bracke, Sarah Gerlo, and Linos Vandekerckhove

Corresponding author: Jolien Van Cleemput (jolien.vancleemput@ugent.be)

Review Timeline:

Submission Date:	1st Sep 25
Editorial Decision:	6th Oct 25
Revision Received:	7th May 26
Editorial Decision:	10th Jun 26
Revision Received:	22nd Jun 26
Accepted:	24th Jun 26

Editor: Zeljko Durdevic

Transaction Report:

(Note: With the exception of the correction of typographical or spelling errors that could be a source of ambiguity, letters and reports are not edited. Depending on transfer agreements, referee reports obtained elsewhere may or may not be included in this compilation. Referee reports are anonymous unless the Referee chooses to sign their reports.)

6th Oct 2025

Dear Prof. Van Cleemput,

Thank you for the submission of your manuscript to EMBO Molecular Medicine. We have now received feedback from the two reviewers who agreed to evaluate your manuscript. As you will see from the reports below, the referees acknowledge the interest of the study but also raise serious and largely overlapping concerns particularly regarding in vivo experiments and experiments with primary cell.

Taking the referee reports in consideration, it is clear that publication of the paper cannot be considered at this stage. I note that addressing the reviewers concerns in full appears to require a lot of additional work and experimentation. Therefore, we think six months rather than three months would be more appropriate to provide the complete revision. If you would like to discuss further the points raised by the referees, I am available to do so via email or video. Let me know if you are interested in this option.

Further consideration of a revision that addresses reviewers' concerns in full will entail a second round of review. EMBO Molecular Medicine encourages a single round of revision only and therefore, acceptance or rejection of the manuscript will depend on the completeness of your responses included in the next, final version of the manuscript. For this reason, and to save you from any frustrations in the end, I would strongly advise against returning an incomplete revision.

We would welcome the submission of a revised version within six months for further consideration. Please let us know if you require longer to complete the revision.

I look forward to receiving your revised manuscript.

Yours sincerely,

Zeljko Durdevic

Zeljko Durdevic
Senior Editor
EMBO Molecular Medicine

*****

When submitting your revised manuscript, please carefully review the instructions that follow below. We perform an initial quality control of all revised manuscripts before re-review; failure to include requested items will delay the evaluation of your revision.

We require:

1) A .docx formatted version of the manuscript text (including legends for main figures, EV figures and tables). Please make sure that the changes are highlighted to be clearly visible.

2) Individual production quality figure files as .eps, .tif, .jpg (one file per figure). For guidance, download the 'Figure Guide PDF': (https://www.embopress.org/page/journal/17574684/authorguide#figureformat).

3) A .docx formatted letter INCLUDING the reviewers' reports and your detailed point-by-point responses to their comments. As part of the EMBO Press transparent editorial process, the point-by-point response is part of the Review Process File (RPF), which will be published alongside your paper.

4) A complete author checklist, which you can download from our author guidelines (https://www.embopress.org/page/journal/17574684/authorguide#submissionofrevisions). Please insert information in the checklist that is also reflected in the manuscript. The completed author checklist will also be part of the RPF.

5) Please note that all corresponding authors are required to supply an ORCID ID for their name upon submission of a revised manuscript.

6) It is mandatory to include a 'Data Availability' section after the Materials and Methods. Before submitting your revision, primary datasets produced in this study need to be deposited in an appropriate public database, and the accession numbers and database listed under 'Data Availability'. Please remember to provide a reviewer password if the datasets are not yet public (see https://www.embopress.org/page/journal/17574684/authorguide#dataavailability).

In case you have no data that requires deposition in a public database, please state so in this section. Note that the Data Availability Section is restricted to new primary data that are part of this study.

7) For data quantification: please specify the name of the statistical test used to generate error bars and P values, the number (n) of independent experiments (specify technical or biological replicates) underlying each data point and the test used to calculate p-values in each figure legend. The figure legends should contain a basic description of n, P and the test applied. Graphs must include a description of the bars and the error bars (s.d., s.e.m.). See also 'Figure Legend' guidelines: https://www.embopress.org/page/journal/17574684/authorguide#figureformat

8) At EMBO Press we ask authors to provide source data for the main manuscript figures. You will receive a separate email with instructions for providing source data with your revised manuscript, including how to upload and organize the files.

9) Our journal encourages inclusion of *data citations in the reference list* to directly cite datasets that were re-used and obtained from public databases. Data citations in the article text are distinct from normal bibliographical citations and should directly link to the database records from which the data can be accessed. In the main text, data citations are formatted as follows: "Data ref: Smith et al, 2001" or "Data ref: NCBI Sequence Read Archive PRJNA342805, 2017". In the Reference list, data citations must be labeled with "[DATASET]". A data reference must provide the database name, accession number/identifiers and a resolvable link to the landing page from which the data can be accessed at the end of the reference. Further instructions are available at .

10) We replaced Supplementary Information with Expanded View (EV) Figures and Tables that are collapsible/expandable online. A maximum of 5 EV Figures can be typeset. EV Figures should be cited as 'Figure EV1, Figure EV2" etc... in the text and their respective legends should be included in the main text after the legends of regular figures.

- For the figures that you do NOT wish to display as Expanded View figures, they should be bundled together with their legends in a single PDF file called *Appendix*, which should start with a short Table of Content. Appendix figures should be referred to in the main text as: "Appendix Figure S1, Appendix Figure S2" etc.

- Additional Tables/Datasets should be labeled and referred to as Table EV1, Dataset EV1, etc. Legends have to be provided in a separate tab in case of .xls files. Alternatively, the legend can be supplied as a separate text file (README) and zipped together with the Table/Dataset file.

See detailed instructions here:

.

11) The paper explained: EMBO Molecular Medicine articles are accompanied by a summary of the articles to emphasize the major findings in the paper and their medical implications for the non-specialist reader. Please provide a draft summary of your article highlighting

- the medical issue you are addressing,

- the results obtained and

- their clinical impact.

This may be edited to ensure that readers understand the significance and context of the research. Please refer to any of our published articles for an example.

12) Author contributions: You will be asked to provide CRediT (Contributor Role Taxonomy) terms in the submission system. These replace a narrative author contribution section in the manuscript.

13) A Conflict of Interest statement should be provided in the main text.

14) Every published paper now includes a 'Synopsis' to further enhance discoverability. Synopses are displayed on the journal webpage and are freely accessible to all readers. They include a short stand first (maximum of 300 characters, including space) as well as 2-5 one-sentences bullet points that summarizes the paper. Please write the bullet points to summarize the key NEW findings. They should be designed to be complementary to the abstract - i.e. not repeat the same text. We encourage inclusion of key acronyms and quantitative information (maximum of 30 words / bullet point). Please use the passive voice. Please attach these in a separate file or send them by email, we will incorporate them accordingly.

Please also suggest a striking image or visual abstract to illustrate your article as a PNG file 550 px wide x 300-600 px high.

15) Include a Reagents and Tools Table as part of the Methods section, which can be downloaded from our author guidelines (https://www.embopress.org/page/journal/17574684/authorguide#structuredmethods)

EMBO Molecular Medicine has a "scooping protection" policy, whereby similar findings that are published by others during review or revision are not a criterion for rejection. Should you decide to submit a revised version, I do ask that you get in touch after three months if you have not completed it, to update us on the status.

***** Reviewer's comments *****

Referee #1 (Remarks for Author):

Summary:
Van Cleemput et al. endeavor to design and optimize in vivo delivery of gene editors in the pursuit of an effective HIV cure approach, with the goal of using CRISPR/Cas9 to inactivate replication competent proviruses in CD4+ HIV-infected cells. They leverage recent advances in the construction and optimization of virus-like particles (VLPs) that covalently encapsulate Cas9-guide RNA ribonucleoprotein (RNP) complexes, termed "nanoblades". However, a major hurdle that remains for many in vivo gene editing platforms, including VLP-based nanoblades, is the need to target RNP complexes to specific cell types in tissues outside of the liver. A novel aspect of the approach described by Van Cleemput and colleagues is the addition of an anti-CD4 targeting nanobody fused to the measles virus hemagglutinin in order to target the VLP nanoblades to CD4+ immune cells, which are known to harbor latent HIV proviruses. The authors demonstrate that CD4-targeted nanoblades can selectively deliver cargo to CD4+ immune cells in vitro and in vivo in humanized mice. CD4-targeted nanoblades were able to efficiently edit cell lines and primary cells in vitro expressing CD4 and at low concentrations. Moreover, when engineered to target an overlapping tat/rev/env region in HIV, CD4-targeted nanoblades reduced HIV infection in vitro. Most importantly, CD4-nanoblades targeting HIV were able to significantly reduce post-rebound viral loads in HIV-infected humanized mice, suggesting they may have clinical relevance. This study addresses an important hurdle for the targeted delivery of gene editors in vivo, which has broad clinical relevance. However, there are some major questions regarding the frequency and efficiency of HIV provirus disruption mediated by CD4-targeted nanoblades that must be clarified in order to accurately interpret the potential impact this approach would have with respect to furthering the goal of an HIV cure.

Major critiques:
1) While editing results are compelling in the context of disrupting HIV proviruses in cell lines (JLats or SupT1s), or highly expressed endogenous genes in primary cells (CD4 disrupted in Figure 3C), there is a lack of compelling data for editing of HIV proviruses in primary CD4+ T cells using CD4-targeted nanoblades. Specifically, while there was a reduction in virus production when HIV-reporter infected CD4+ T cells were treated with Tat1-2 nanoblades (Figure 5C), there was no corresponding gene disruption identified in HIV sequences amplified from those infected/treated cells (at least when looking for excised product which requires 2 simultaneous cuts). Though the frequency of infected cells was indeed low (Figure 5B), and this could have affected the sensitivity to detect gene disruption, these experiments should be repeated with optimized conditions. It is typical to achieve 20 to 60% p24+ cells when infecting stimulated primary CD4+ T cells with a high MOI of replication competent HIV, especially when using CXCR4-tropic variants. Achieving a higher infection rate in these important in vitro studies may help to achieve detection of provirus editing by nanoblades, which is a critical piece of data.

2) The results depicted in Figure 6 are impressive, as plasma viral loads are typically difficult to alter in humanized mouse models, where plasma viremia is typically high and the endogenous immune response is weak. However, these impressive results are seemingly paradoxical. The goal of these studies was to deliver CD4-targeted nanoblades while HIV-infected humanized mice are suppressed on ART, in order to disrupt otherwise replication competent proviruses, and thus affect the kinetics or peak of rebound viremia. However, neither kinetics nor peak rebound viremia were consistently affected in either study, but rather the post peak set point viremia, with some signal in peak viral loads from experiment 1. Significant discussion should be added to the manuscript in order to propose mechanisms that may explain this result.
Additionally, and critical to adding mechanistic detail to the in vivo studies, deep sequencing of amplified HIV provirus should be performed to determine the frequency of nanoblade-mediated indels at the gRNA target sites. While this would be most informative in a repeat study where mice are treated with nanoblades and then necropsied under ART, it is possible that even after HIV rebound, nanoblade-edited cells should persist as production HIV gene expression should be blocked or attenuated in these cells.

3) The lack of congruence between CD4 downregulation and CD4 gene disruption in Figure 3C-D should be further interrogated. While results are congruent for donor 1, they are not for donors 2 and 3. While it is true that sequencing bias could have affected results, efficient gene disruption should be evident. Other techniques with droplet-digital PCR probes, deep sequencing, or larger amplicons could be used to clear up this discrepancy.
Otherwise, is it possible that CD4-targeted nanobodies cause receptor-mediated endocytosis leading to transient downregulation of CD4 without concomitant gene disruption? While the CD4-NB scramble should pose an adequate control for this, demonstrating whether this occurs or not and at what doses could help rule out this confounder.

Minor critiques:
1) In the experiment with infection of primary CD4+ T cells with the eGFP reporter HIV (Figure 5), it is not discussed why Tat-focused nanoblades reduce the infected cells (by GFP MFI), but eGFP nanoblades do not, even though the latter would likely be even more direct.

2) While it is assumed toxicity is low, it would be helpful to determine the toxicity profile of a range of concentrations of CD4-targeted nanoblades on primary CD4+ T cells.

3) For figure 2E, it would be more informative to also include a targeting readout as frequency of mCherry+ cells rather than MFI alone, as frequency of targeting is more helpful for evaluating the efficiency of the strategy.
Additionally, this would help determine whether CD4-nanoblade targeting in the spleen or bone marrow is inefficient, or non-specific, as this comparison to PBMC is difficult to make from MFI alone.

4) In Figures 4B and 5B, it would be informative to have the frequencies denoted in the flow plots.

Referee #2 (Comments on Novelty/Model System for Author):

1. More primary cells need to be tested in vitro to conclude the anti-HIV effects.
2. There is no statistic analysis for some in vivo data in the humanized mice models.

Referee #2 (Remarks for Author):

This manuscript described a novel CRISPR-based gene editing system (CD4-directed nanoblades targeting HIV tat locus) that has the potential for HIV elimination/cure. The authors paid great efforts to carefully characterize the CD4-directed nanoblades, including their biochemical and physicochemical properties (Figure 1), their selective binding to CD4+ cells for entry (Figure 2), as well as their specific gene editing in these cells (Figure 3). However, the results are unconvincing regarding the therapeutic potential of these nanoblades for inhibiting/eliminating HIV. Both in vitro and in vivo anti-HIV effects of Tat1-2 CD4-NB appear to be poor. Here are major weaknesses.
1. Figure 4, what is the exact impact of Tat1-2 CD4-NB on HIV proviral genome? A thorough profiling of Tat1-2 CD4-NB induced truncation of HIV tat locus is needed. What is the portion (percentage) of HIV proviruses that are truncated by Tat1-2 CD4-NB?
2. Figure 4 & 5, The p24 level is only modestly reduced due to high dose of Tat1-2 CD4-NB in J-Lat 10.6 cells. Likewise, the HIV viral titer is only modestly reduced due to Tat1-2 CD4-NB in Sup-T1 cells. The results in primary CD4+ cells appear encouraging, but how many donors have been tested for this assay? This is critical to test the anti-HIV effect using primary CD4+ cells from multiple donors to reach the conclusions.
3. For Figure 4B and 5B, there is no quantification regarding the percentage of eGFP-positive cell population.
4. The data in Figure 5B and 5C are not well correlated in primary CD4+ cells. Tat1-2 CD4-NB almost has no effect on eGFP MFI while significantly decreasing HIV viral titer in primary CD4+ cells. Such discrepancy needs to be resolved.
5. Figure 6, it appears that Tat1-2 CD4-NB barely works in the humanized mice models of HIV infection. There is no statistical analysis for quantification data in Figure 6B. Beyond viral load (viral RNAs), does the Tat1-2 CD4-NB in vivo treatment reduce HIV viral reservoirs (viral DNAs) in blood and/or tissues? The authors could consider measuring HIV viral reservoirs in vivo by QVOA and/or IPDA assays.

Referee #3 (Comments on Novelty/Model System for Author):

While this study presents an interesting new strategy to pseudotype VLPs for targeted delivery to CD4-expressing cells, I remain concerned about the strength of the evidence supporting its effectiveness in primary cells and in vivo. The manuscript does not provide direct proof that genome editing has occurred in these settings, and the in vivo effects reported (using humanized mice) are modest and unlikely to be clinically relevant, especially in fully immunocompetent human individuals.

Referee #3 (Remarks for Author):

In this manuscript, Cleemput JV and collaborators describe the use of Nanoblades (Cas9/sgRNA-loaded retroviral virus-like particles) combined with a new pseudotyping approach. This strategy is based on a mutant VSV-G glycoprotein that has lost receptor recognition but retains fusogenic activity, co-expressed on the surface of VLPs with a truncated measles virus hemagglutinin fused to an anti-CD4 nanobody. Using this pseudotyping approach with mCherry-loaded Nanoblades, the authors demonstrate specific transduction of CD4-positive cells in PBMC populations as well as in vivo in a humanized mouse model. With Cas9-loaded VLPs, they further show gene editing in CD4-positive SupT1 cells and in primary CD4+ T cells. The authors then test CD4-targeting VLPs carrying two sgRNAs directed against conserved loci within the HIV-1 genome coding for the Tat protein, or GFP in a CD4-positive cell line harboring a latent, non-replicative HIV-1 provirus. In this system, they report efficient inactivation of proviral gene expression. These results are subsequently validated using a replicative HIV-1 strain in SupT1 cells and primary CD4+ T cells, demonstrating a reduction in viral titers. Finally, the authors test CD4-targeted Nanoblades in a humanized mouse model infected with HIV-1 and observe a significant drop in plasma viral RNA copies after repeated Nanoblade injections.
Overall, the manuscript is well written and presents an interesting technical approach for mediating target-specific delivery of protein cargos into CD4-positive cells. Results obtained in PBMCs and cell lines convincingly demonstrate specific transduction of CD4-positive cells. Data in cell lines and primary T cells also show clear targeted editing of endogenous loci (in primary CD4+ T cells) or integrated GFP copies (in cell lines). The experiments aimed at inactivating HIV-1 in primary cells and in vivo demonstrate functional effects, however, they do not yet clearly establish or quantify the underlying editing efficiency. Below, I provide a list of major and minor comments that should be addressed to strengthen the validation of this tool in vivo.

Major points:
Figure 3C : It is difficult to compare the amount of Cas9 delivered between Nanoblades and RNP electroporation since Nanoblades will deliver their content throughout the incubation time (a VLP that has not encountered a cell in the first few minutes of transduction will still be able to encounter a cell later and deliver its content) while, during electroporation, RNPs can only enter cells during the short electric pulse. It is therefore not surprising that electroporation requires higher amounts of Cas9/sgRNA RNP molecules than VLPs. Furthermore, the example chosen for the figure corresponds to the best replicate for Nanoblades (which on the contrary was the worst replicate for electroporation). To better assess reproducibility, could the authors repeat this experiment using different Nanoblade batches in a single donor to evaluate batch-to-batch variability?

Figure 5B : The percentage of infected cells is very low, particularly in primary CD4+ cells. Were the authors aiming for a maximum of one proviral DNA copy per cell? At such low infection rates, variability between samples can be high, which makes the interpretation of these data more difficult. This is especially relevant since eGFP CD4-NBs, which performed better than Tat 1-2 CD4-NBs in previous assays, did not show a significant effect in this experiment. Would the authors consider repeating the experiment with a higher MOI to increase infection rates? In addition, from the methods it is not entirely clear whether resting cells were infected, then transduced with NBs, and subsequently activated, or whether cells were activated prior to infection. Would activating cells before infection and then transducing them with NBs provide a more robust system?

Figure 5C/D : The strong reduction in viral titers following Tat 1-2 CD4-NB treatment is surprising given that the excised segment was not detected. One possible explanation is that even if only one of the two sgRNAs targeting Tat introduces indels, the resulting disruption could impair Tat protein function. Could the authors perform Sanger sequencing at the Tat1 loci in primary CD4 cells to assess whether indels are detectable? This would help clarify whether the observed reduction in viral titers can be attributed to editing events. If no indels are detectable, it becomes more difficult to exclude alternative explanations for the reduction in viral titers.

Figure 6 : Although the Tat 1-2 CD4-NBs induced a significant reduction in viral titers in treated mice, the effect appears modest and may not correspond to a clinically meaningful restriction of HIV-1 replication. Moreover, no direct evidence of specific gene editing at the targeted HIV-1 loci is provided. To strengthen this point, it would be important to measure indel rates shortly after in vivo transduction to demonstrate genome editing activity. In the absence of such data, it is difficult to distinguish specific from non-specific effects. If assessing indels at proviral loci proves technically challenging, an alternative approach could be to target an endogenous CD4+ T cell locus in vivo to provide evidence of editing efficiency. Additionally, as humanized mice lack functional complement and innate immunity, one might expect the observed effects to be even milder in humans with a fully competent immune system.
Finally, there seems to be a discrepancy between Figure 2B bottom left panel (Exp2) and the bottom right panel (Exp2) for the 3w P HIV NB condition. On the bottom left, the four highest HIV-1 titers correspond to controls, followed by a mix of Tat 1-2

CD4-NBs and control mice, whereas on the bottom right, the five highest values correspond to controls. Could the authors clarify this point?

Minor comments:
Line 98-100 : Authors describe the particle size distribution and zeta potential of the produced Nanoblades but they do not comment these values. For non specialist readers, this information will be difficult to interpret.

Figure 3A : CD4-NBs appear to mediate GFP inactivation at higher VLP amounts (20pg per cell) even in CD4 negative cells. Because the SupT1 CD4+ and CD4- cells do not appear to display the same number of GFP copies, it is difficult to conclude regarding the specificity of the CD4-NBs. Could the authors perform

Line 299-301 : Authors compare their CD4-nanobody pseudotyping strategy to that of HIV-1 envelope pseudotyping described in Hamilton JR 2021. However, it is not straightforward to make such a comparison since Hamilton JR used a hybrid VLP that co-delivers a transgene together with the Cas9 and this could affect the efficiency of Cas9 delivery. Furthermore, the authors claim over 80 % of transduction of primary T CD4 cells but in Figure 3C/D only 70 % of gene editing efficiency was achieved and this was for only one of the three donors as the other two donors showed a 20 % gene editing efficiency.

Dear reviewers,

Thank you for your thorough revisions and critical reflections to improve our manuscript. Please find our detailed answers to your suggestions and concerns below, written in blue. In the new manuscript version, adaptations to the original manuscript are visible in track changes.

Sincerely,
On behalf of all authors,
Prof. dr. Jolien Van Cleemput and Prof. dr. Linos Vandekerckhove

****

Referee #1:

Summary

Van Cleemput et al. endeavor to design and optimize in vivo delivery of gene editors in the pursuit of an effective HIV cure approach, with the goal of using CRISPR/Cas9 to inactivate replication competent proviruses in CD4+ HIV-infected cells. They leverage recent advances in the construction and optimization of virus-like particles (VLPs) that covalently encapsulate Cas9-guide RNA ribonucleoprotein (RNP) complexes, termed "nanoblades". However, a major hurdle that remains for many in vivo gene editing platforms, including VLP-based nanoblades, is the need to target RNP complexes to specific cell types in tissues outside of the liver. A novel aspect of the approach described by Van Cleemput and colleagues is the addition of an anti-CD4 targeting nanobody fused to the measles virus hemagglutinin in order to target the VLP nanoblades to CD4+ immune cells, which are known to harbor latent HIV proviruses. The authors demonstrate that CD4-targeted nanoblades can selectively deliver cargo to CD4+ immune cells in vitro and in vivo in humanized mice. CD4-targeted nanoblades were able to efficiently edit cell lines and primary cells in vitro expressing CD4 and at low concentrations. Moreover, when engineered to target an overlapping tat/rev/env region in HIV, CD4-targeted nanoblades reduced HIV infection in vitro. Most importantly, CD4-nanoblades targeting HIV were able to significantly reduce post-rebound viral loads in HIV-infected humanized mice, suggesting they may have clinical relevance. This study addresses an important hurdle for the targeted delivery of gene editors in vivo, which has broad clinical relevance. However, there are some major questions regarding the frequency and efficiency of HIV provirus disruption mediated by CD4-targeted nanoblades that must be clarified in order to accurately interpret the potential impact this approach would have with respect to furthering the goal of an HIV cure.

Thank you for the positive comments and for the critical reflection on HIV proviral disruption. We have now addressed these issues in the revised manuscript. Please find our detailed answers to your comments below.

Major critiques:

1) While editing results are compelling in the context of disrupting HIV proviruses in cell lines (JLats or SupT1s), or highly expressed endogenous genes in primary cells (CD4 disrupted in Figure 3C), there is a lack of compelling data for editing of HIV proviruses in primary CD4+ T cells using CD4-targeted nanoblades. Specifically, while there was a reduction in virus production when HIV-reporter infected CD4+ T cells were treated with Tat1-2 nanoblades (Figure 5C), there was no corresponding gene disruption identified in HIV sequences amplified from those infected/treated cells (at least when looking for excised product which requires 2 simultaneous cuts). Though the frequency of infected cells was indeed low (Figure 5B), and this could have affected the sensitivity to detect gene disruption, these experiments should be repeated with optimized conditions. It is typical to achieve 20 to 60% p24+ cells when infecting stimulated primary CD4+ T cells with a high MOI of replication competent HIV, especially when using CXCR4-tropic variants. Achieving a higher infection rate in these important in vitro studies may help to achieve detection of provirus editing by nanoblades, which is a critical piece of data.

In the first HIV infection experiment, we used a moi of 0.05 to infect unstimulated primary CD4$^+$ cells and reached only 2-4 % of HIV-infected cells at the end of the experiment (4 days PI). This probably explains why we were unable to detect HIV proviral excision using gel electrophoresis. As suggested, we have now repeated the experiment on three new donors using a 4-fold higher moi (0.2). Higher moi were technically not feasible due to production limitations. Still, this % was enough to demonstrate the effect of our tat1-2 CD4-NBs on replication-competent HIV-infected primary CD4$^+$ cells (Figure 5B). In addition, we have now analysed the tat PCR amplicons using next-generation sequencing based on Oxford-nanopore technologies (ONT) for the excised fragment and could detect this fragment with a read abundance up to 5,47% (Figure 5D). These results have been added in lines 212-214 and discussed in lines 350-355. The data of the original infection experiment has been moved to Figure EV4.

We also attempted infection and treatment of activated CD4$^+$ cells. However, cell viability was insufficient to proceed with CD4-NB treatment. We therefore used non-activated CD4$^+$ T cells for subsequent infection experiments.

2) The results depicted in Figure 6 are impressive, as plasma viral loads are typically difficult to alter in humanized mouse models, where plasma viremia is typically high and the endogenous immune response is weak. However, these impressive results are seemingly paradoxical. The goal of these studies was to deliver CD4-targeted nanoblades while HIV-infected humanized mice are suppressed on ART, in order to disrupt otherwise replication competent proviruses, and thus affect the kinetics or peak of rebound viremia. However, neither kinetics nor peak rebound viremia were consistently affected in either study, but rather the post peak set point viremia, with some signal in peak viral loads from experiment 1. Significant discussion should be added to the manuscript in order to propose mechanisms that may explain this result.
Additionally, and critical to adding mechanistic detail to the in vivo studies, deep sequencing of amplified HIV provirus should be performed to determine the frequency of nanoblade-mediated indels at the gRNA target sites. While this would be most informative in a repeat study where mice are treated with nanoblades and then necropsied under ART, it is possible that even after HIV rebound, nanoblade-edited cells should persist as production HIV gene expression should be blocked or attenuated in these cells.

Thank you for the positive acknowledgement on the efficacy of our CD4-NBs *in vivo*. We agree with this comment and have now additionally analysed the tat PCR amplicons of spleens, bone marrow, and lungs on the repeated dose experiment (exp 2) via Sanger sequencing and identified *in vivo* HIV gene editing in 3 out of 7 CD4 NB-treated mice, demonstrating HIV proviral disruption *in vivo*. Furthermore, we have also now determined the number of HIV proviral copies per 10^6 human cells in these organs. Interestingly, there was a trend for lower HIV copies in tat 1-2 CD4-NB-treated mice compared to control in the lungs (p = 0.081). These results have been added to Figure 6D and lines 254-261. We have also discussed these results in more detail in lines 356-362.

3) The lack of congruence between CD4 downregulation and CD4 gene disruption in Figure 3C-D should be further interrogated. While results are congruent for donor 1, they are not for donors 2 and 3. While it is true that sequencing bias could have affected results, efficient gene disruption should be evident. Other techniques with droplet-digital PCR probes, deep sequencing, or larger amplicons could be used to clear up this discrepancy.

We agree with this comment and believe this discrepancy was related to steps between DNA extraction and INDEL analysis. Therefore, we have repeated this experiment on 3 additional donors to demonstrate broader utility of the CD4-NBs, yet still observed these discrepancies. To exclude potential bias introduced by Sanger sequencing and subsequent sequence trace decomposition, we additionally assessed the mutation frequency around the Cas9 cut site in PCR amplicons from three donors using Oxford nanopore technology (ONT) sequencing (Figure EV3A-B). The percentage of INDELs detected did not significantly differ between both methods ($F_{1,12} = 0.086$, $p = 0.774$). Still, this does not rule out

potential PCR bias. Alternatively, a disproportionate impact of frameshift mutations or allelic effects in certain donors could explain these variations. This has been added to lines 158-166.

Otherwise, is it possible that CD4-targeted nanobodies cause receptor-mediated endocytosis leading to transient downregulation of CD4 without concomitant gene disruption? While the CD4-NB scramble should pose an adequate control for this, demonstrating whether this occurs or not and at what doses could help rule out this confounder.

It is true that two days post CD4-NB transduction, CD4 surface expression in primary $CD4^{+}$ cells is about ± 1.5-fold lower in scrambled- as well as in *CD4*-targeted CD4-NBs (40 fg Cas9/cell) compared to mock-transduced cells, likely due to internalisation. However, CD4 expression reported in our manuscript is analysed 5 days post transduction. At this time, CD4 expression in scrambled CD4-NB-treated primary $CD4^{+}$ cells has been restored and is similar to mock-treated cells, while that of CD4 CD4-NB-treated cells is 1.6-fold lower.

Minor critiques:

1) In the experiment with infection of primary CD4+ T cells with the eGFP reporter HIV (Figure 5), it is not discussed why Tat-focused nanoblades reduce the infected cells (by GFP MFI), but eGFP nanoblades do not, even though the latter would likely be even more direct.

This was likely due to the low % of infection in the experiment reported in the first manuscript version, since we now do observe an effect on eGFP expression by eGFP CD4-NBs in $HIV_{NLENG1\text{-}IRES}$ infected primary $CD4^{+}$ cells (Figure 5B lower panel).

2) While it is assumed toxicity is low, it would be helpful to determine the toxicity profile of a range of concentrations of CD4-targeted nanoblades on primary CD4+ T cells.

We have repeated the experiment on three new donors using an even higher dose of CD4-NBs (160 fg Cas9/cell), confirming even better CD4 knockout without apparent toxicity. These results have been added to lines 170-175 and Figure EV3A.

3) For figure 2E, it would be more informative to also include a targeting readout as frequency of mCherry+ cells rather than MFI alone, as frequency of targeting is more helpful for evaluating the efficiency of the strategy.

Additionally, this would help determine whether CD4-nanoblade targeting in the spleen or bone marrow is inefficient, or non-specific, as this comparison to PBMC is difficult to make from MFI alone.

We agree with the reviewer and now included these data in Figure EV2C.

4) In Figures 4B and 5B, it would be informative to have the frequencies denoted in the flow plots.

We decided not to include the % of HIV-positive cells, because there is little to no effect on this parameter. This is mainly because edited cells still emit remnant eGFP from before eGFP knockout. Therefore, the effect is mainly visible on MFI which was reported in Figures 4B and 5B.

Referee #2

1. More primary cells need to be tested in vitro to conclude the anti-HIV effects.

We have repeated the HIV infection experiment on primary $CD4^{+}$ cells on three new donors using a higher moi and performed additional DNA analyses to confirm HIV proviral excision. The data of the original experiment on three other donors have been moved to Figure EV4 (see below).

2. There is no statistic analysis for some in vivo data in the humanized mice models.

We have included the statistical analyses for our *in vivo* data in the legend of Figure 6 and Method section "Quantification and statistical analysis".

This manuscript described a novel CRISPR-based gene editing system (CD4-directed nanoblades targeting HIV tat locus) that has the potential for HIV elimination/cure. The authors paid great efforts to carefully characterize the CD4-directed nanoblades, including their biochemical and physicochemical properties (Figure 1), their selective binding to CD4+ cells for entry (Figure 2), as well as their specific gene editing in these cells (Figure 3). However, the results are unconvincing regarding the therapeutic potential of these nanoblades for inhibiting/eliminating HIV. Both in vitro and in vivo anti-HIV effects of Tat1-2 CD4-NB appear to be poor. Here are major weaknesses.

Thank you for the positive comments. We agree with this point and have addressed it through additional experiments and DNA analyses, as described below.

1. Figure 4, what is the exact impact of Tat1-2 CD4-NB on HIV proviral genome? A thorough profiling of Tat1-2 CD4-NB induced truncation of HIV tat locus is needed. What is the portion (percentage) of HIV proviruses that are truncated by Tat1-2 CD4-NB?

We understand this point and have repeated the HIV infection experiments in primary $CD4^+$ cells. As the excised fragment was not detectable by gel electrophoresis, we assessed HIV proviral excision using ONT sequencing (see below). In contrast, excision in J-Lat cells was readily detectable by gel electrophoresis. Therefore, due to time and cost considerations, ONT sequencing was not performed on J-Lat DNA.

2. Figure 4 & 5, The p24 level is only modestly reduced due to high dose of Tat1-2 CD4-NB in J-Lat 10.6 cells. Likewise, the HIV viral titer is only modestly reduced due to Tat1-2 CD4-NB in Sup-T1 cells. The results in primary CD4+ cells appear encouraging, but how many donors have been tested for this assay? This is critical to test the anti-HIV effect using primary CD4+ cells from multiple donors to reach the conclusions.

We have repeated this experiment on three new donors using a higher moi, bringing the total number of donors to 6. Using Oxford nanopore sequencing, proviral excision was detected in 2 out of 3 donors (Figure 5D). Consistent effects on eGFP MFI and viral titers in these donors are shown in Figure 5B. Data from the original experiment performed at lower moi have been moved to Figure EV4.

3. For Figure 4B and 5B, there is no quantification regarding the percentage of eGFP-positive cell population.

We decided not to include the % of HIV-positive cells, because there is little to no effect on this parameter. This is mainly because edited cells still emit remnant eGFP from before the eGFP knockout. Therefore, the effect is mainly visible on MFI which was reported in Figures 4B and 5B.

4. The data in Figure 5B and 5C are not well correlated in primary CD4+ cells. Tat1-2 CD4-NB almost has no effect on eGFP MFI while significantly decreasing HIV viral titer in primary CD4+ cells. Such discrepancy needs to be resolved.

This discrepancy was likely related to the fact that there was still eGFP expression in tat-edited cells, since eGFP is located downstream of the tat locus and remains to be expressed independent of tat. However, the capacity to produce infectious virions was affected, resulting in lower virus titers. As described above, we have repeated this experiment using a higher moi to be able to pick up excision of HIV proviral DNA using Oxford nanopore sequencing (Figure 5D). The effect on eGFP MFI and virus titer in these donors is also apparant in Figure 5B. Data from the original experiment performed at lower moi have been moved to Figure EV4.

5. Figure 6, it appears that Tat1-2 CD4-NB barely works in the humanized mice models of HIV infection. There is no statistical analysis for quantification data in Figure 6B.

We have explained the statistical analysis of the mice experiments briefly in the Figure legend, as well as in the Method section under "Quantification and statistical analysis".

Beyond viral load (viral RNAs), does the Tat1-2 CD4-NB in vivo treatment reduce HIV viral reservoirs (viral DNAs) in blood and/or tissues? The authors could consider measuring HIV viral reservoirs in vivo by QVOA and/or IPDA assays.

We agree with this comment and have now analysed the number of HIV RU5 and env copies per 10^6 million human cells in bone marrow, spleen, and lungs of mice in exp 2 using dPCR. These new data have been included in Figure 6D and in lines 254-261. The *in vivo* results are further discussed in lines 356-362.

Referee #3

While this study presents an interesting new strategy to pseudotype VLPs for targeted delivery to CD4-expressing cells, I remain concerned about the strength of the evidence supporting its effectiveness in primary cells and in vivo. The manuscript does not provide direct proof that genome editing has occurred in these settings, and the in vivo effects reported (using humanized mice) are modest and unlikely to be clinically relevant, especially in fully immunocompetent human individuals.

In this manuscript, Cleemput JV and collaborators describe the use of Nanoblades (Cas9/sgRNA-loaded retroviral virus-like particles) combined with a new pseudotyping approach. This strategy is based on a mutant VSV-G glycoprotein that has lost receptor recognition but retains fusogenic activity, co-expressed on the surface of VLPs with a truncated measles virus hemagglutinin fused to an anti-CD4 nanobody. Using this pseudotyping approach with mCherry-loaded Nanoblades, the authors demonstrate specific transduction of CD4-positive cells in PBMC populations as well as in vivo in a humanized mouse model. With Cas9-loaded VLPs, they further show gene editing in CD4-positive SupT1 cells and in primary CD4+ T cells. The authors then test CD4-targeting VLPs carrying two sgRNAs directed against conserved loci within the HIV-1 genome coding for the Tat protein, or GFP in a CD4-positive cell line harboring a latent, non-replicative HIV-1 provirus. In this system, they report efficient inactivation of proviral gene expression. These results are subsequently validated using a replicative HIV-1 strain in SupT1 cells and primary CD4+ T cells, demonstrating a reduction in viral titers. Finally, the authors test CD4-targeted Nanoblades in a humanized mouse model infected with HIV-1 and observe a significant drop in plasma viral RNA copies after repeated Nanoblade injections.

Overall, the manuscript is well written and presents an interesting technical approach for mediating target-specific delivery of protein cargos into CD4-positive cells. Results obtained in PBMCs and cell lines convincingly demonstrate specific transduction of CD4-positive cells. Data in cell lines and primary T cells also show clear targeted editing of endogenous loci (in primary CD4+ T cells) or integrated GFP copies (in cell lines). The experiments aimed at inactivating HIV-1 in primary cells and in vivo demonstrate functional effects, however, they do not yet clearly establish or quantify the underlying editing efficiency. Below, I provide a list of major and minor comments that should be addressed to strengthen the validation of this tool in vivo.

Thank you for the positive feedback on our manuscript and we agree with these issues. Therefore, we have now performed additional experiments and analyses to show editing in (HIV-infected) primary $CD4^+$ cells *in vitro* and *in vivo*, as further specified below.

<u>Major points:</u>

Figure 3C : It is difficult to compare the amount of Cas9 delivered between Nanoblades and RNP electroporation since Nanoblades will deliver their content throughout the incubation time (a VLP that has not encountered a cell in the first few minutes of transduction will still be able to encounter a cell

later and deliver its content) while, during electroporation, RNPs can only enter cells during the short electric pulse. It is therefore not surprising that electroporation requires higher amounts of Cas9/sgRNA RNP molecules than VLPs. Furthermore, the example chosen for the figure corresponds to the best replicate for Nanoblades (which on the contrary was the worst replicate for electroporation). To better assess reproducibility, could the authors repeat this experiment using different Nanoblade batches in a single donor to evaluate batch-to-batch variability?

To ensure reproducibility, we have repeated this experiment on three additional donors and included these in Figure 3C-D (D4-6). The effects on MFI and genomic editing varied substantially between donors and between CD4-NBs and RNP electroporation. Therefore, we reported each donor as a separate dot on the graph. We agree with the fact that for RNP there is a much shorter time for the RNP to get in the cells and also have acknowledged this in lines 326-328. Finally, during this repeat experiment we also tested two additional batches of CD4 CD4-NBs at a high dose (160 fg Cas9/cell) in donor 6, confirming reproducibility between batches when normalized for Cas9 dose (Figure EV3E).

Figure 5B : The percentage of infected cells is very low, particularly in primary CD4+ cells. Were the authors aiming for a maximum of one proviral DNA copy per cell? At such low infection rates, variability between samples can be high, which makes the interpretation of these data more difficult. This is especially relevant since eGFP CD4-NBs, which performed better than Tat 1-2 CD4-NBs in previous assays, did not show a significant effect in this experiment. Would the authors consider repeating the experiment with a higher MOI to increase infection rates? In addition, from the methods it is not entirely clear whether resting cells were infected, then transduced with NBs, and subsequently activated, or whether cells were activated prior to infection. Would activating cells before infection and then transducing them with NBs provide a more robust system?

In the first HIV infection experiment, we used a moi of 0.05 to infect unstimulated primary $CD4^+$ cells and reached only 2-4 % of HIV-infected cells at the end of the experiment (4 days PI). This probably explains why we were unable to detect HIV proviral excision using gel electrophoresis. As suggested, we have now repeated the experiment on three new donors using a 4-fold higher moi (0.2). Higher moi were technically not feasible due to production limitations. Still, this % was enough to demonstrate the effect of our tat1-2 CD4-NBs on replication-competent HIV-infected primary $CD4^+$ cells (Figure 5B). In addition, we have now analysed the tat PCR amplicons using next-generation sequencing based on Oxford-nanopore technologies (ONT) for the excised fragment and could detect this fragment with a read abundance up to 5,47% (Figure 5D). These results have been added in lines 212-214 and discussed in lines 350-355. The data of the original infection experiment has been moved to Figure EV4.

We also attempted infection and treatment of activated $CD4^+$ cells. However, cell viability was insufficient to proceed with CD4-NB treatment. We therefore used non-activated $CD4^+$ T cells for subsequent infection experiments.

Figure 5C/D : The strong reduction in viral titers following Tat 1-2 CD4-NB treatment is surprising given that the excised segment was not detected. One possible explanation is that even if only one of the two sgRNAs targeting Tat introduces indels, the resulting disruption could impair Tat protein function. Could the authors perform Sanger sequencing at the Tat1 loci in primary CD4 cells to assess whether indels are detectable? This would help clarify whether the observed reduction in viral titers can be attributed to editing events. If no indels are detectable, it becomes more difficult to exclude alternative explanations for the reduction in viral titers.

As stated above, we have now provided evidence for HIV gene excision in HIV-infected primary $CD4^+$ cells using ONT sequencing. These results have been added to Figure 5D and lines 212-214.

Figure 6 : Although the Tat 1-2 CD4-NBs induced a significant reduction in viral titers in treated mice, the effect appears modest and may not correspond to a clinically meaningful restriction of HIV-1 replication. Moreover, no direct evidence of specific gene editing at the targeted HIV-1 loci is provided.

To strengthen this point, it would be important to measure indel rates shortly after in vivo transduction to demonstrate genome editing activity. In the absence of such data, it is difficult to distinguish specific from non-specific effects. If assessing indels at proviral loci proves technically challenging, an alternative approach could be to target an endogenous CD4+ T cell locus in vivo to provide evidence of editing efficiency. Additionally, as humanized mice lack functional complement and innate immunity, one might expect the observed effects to be even milder in humans with a fully competent immune system.

We analysed the tat locus for INDELs in the bone marrow, spleens, and lungs of mice and found INDELs at this locus in 3 out of 7 mice in bone marrow and lungs. These data have now been included in Figure 6D and lines 258-261.

Finally, there seems to be a discrepancy between Figure 2B bottom left panel (Exp2) and the bottom right panel (Exp2) for the 3w P HIV NB condition. On the bottom left, the four highest HIV-1 titers correspond to controls, followed by a mix of Tat 1-2 CD4-NBs and control mice, whereas on the bottom right, the five highest values correspond to controls. Could the authors clarify this point?

We have double checked these figures, but the data in Figure 6B bottom left panel are the same as the ones represented in the right panel for Exp3 3w P HIV NV. The exact data can be found in the source data files.

<u>Minor comments:</u>

Line 98-100 : Authors describe the particle size distribution and zeta potential of the produced Nanoblades but they do not comment these values. For non specialist readers, this information will be difficult to interpret.

This is a good suggestion, and we have now clarified this in lines 99-103.

Figure 3A : CD4-NBs appear to mediate GFP inactivation at higher VLP amounts (20pg per cell) even in CD4 negative cells. Because the SupT1 CD4+ and CD4- cells do not appear to display the same number of GFP copies, it is difficult to conclude regarding the specificity of the CD4-NBs. Could the authors perform

$eGFP^{+}$ $CD4^{+}$ and $CD4^{-}$ Sup-T1cells were generated using the same lentiviral vector stock at the same low moi of 0.01 and subsequently sorted based on eGFP emission. Therefore, it is highly unlikely that differences in expression are due to variation in copy number between cells. The observed differences in fluorescence intensity may be attributed to differences in genomic integration sites. To account for this variability in eGFP expression, MFI values were normalized to mock-treated controls.

Line 299-301 : Authors compare their CD4-nanobody pseudotyping strategy to that of HIV-1 envelope pseudotyping described in Hamilton JR 2021. However, it is not straightforward to make such a comparison since Hamilton JR used a hybrid VLP that co-delivers a transgene together with the Cas9 and this could affect the efficiency of Cas9 delivery.

We agree with this statement and have more nuanced this expression in lines 330-332.

Furthermore, the authors claim over 80 % of transduction of primary T CD4 cells but in Figure 3C/D only 70 % of gene editing efficiency was achieved and this was for only one of the three donors as the other two donors showed a 20 % gene editing efficiency.

We agree with this statement and have more nuanced this expression in lines 330-332.

10th Jun 2026

Dear Prof. Van Cleemput,

Thank you for the submission of your revised manuscript to EMBO Molecular Medicine As you will see from their reports pasted below, referee #2 remains critical particularly regarding the low clinical relevance, while the referees #1 and #3 support publication of the manuscript but also note low medical implication at this stage. Based on the initial referee reports and after an editorial discussion, we agreed that the authors responded adequately to the referees' criticism and that the manuscript fits the scope of EMBO Molecular Medicine. Therefore, I am pleased to inform you that we will be able to accept your manuscript pending the following final amendments:

1) We agree with shared referees' opinion that the study shows limited translational implication and that it rather represents a proof-of-concept. Please indicate in the abstract and in the manuscript where appropriate that this is a proof-of-concept study. Also, clearly state the limitation of the study regarding low in vivo efficiency and limited clinical implications. Please address referee #2 concerns by discussion. No additional experiments are required.
2) In the main manuscript file, please do the following:
- Please address all comments suggested by our data editors listed below:
o Figure legends:
1. Please note that the exact p values are not provided in the legends of figures 2E, 5B, EV3 C, E; EV4.
2. Please note that information related to n is missing in the legends of figures EV5 A, B.
3. Please note that the error bars are not defined in the legends of figures 3B, 4C, 6B-D; EV5 C.
- Add callouts for Figure 6E.
- Remove data not shown (p. 6 and 11).
- Please remove Reagents and Tools Table and uploaded it as a separate .docx file. More information on how to adhere to this format as well as downloadable templates (.docx) for the Reagents and Tools Table can be found in our author guidelines:
https://www.embopress.org/page/journal/17574684/authorguide#structuredmethods
- Please rename "Methods and Protocols" to "Methods.
- In Methods, provide the antibody dilutions that were used for each antibody.
- Indicate in legends exact n and exact p values, not a range, along with the statistical test used. To keep the figures "clear" some authors found providing an Appendix table Sx with all exact p-values preferable. You are welcome to do this if you want to.
- Author contributions: Please remove it from the manuscript and specify author contributions in our submission system. CRediT has replaced the traditional author contributions section because it offers a systematic machine-readable author contributions format that allows for more effective research assessment. You are encouraged to use the free text boxes beneath each contributing author's name to add specific details on the author's contribution. More information is available in our guide to authors:
https://www.embopress.org/page/journal/17574684/authorguide#authorshipguidelines
- Remove the sentence "All data can be found in the source data files." From data availability statement.
- Please change nomenclature of EV figures in the legends to Figure EV1 etc.
3) Tables: Combine the 3 tables into Table 1: Primers and Probes and place it between main and EV figure legends. Please update table callouts.
4) Synopsis:
- Synopsis image: Please provide a visual abstract as a high-resolution jpeg file exactly 550 px-wide x 300 pixels high to illustrate your article.
- Please check your synopsis text and image before submission with your revised manuscript. Please be aware that in the proof stage minor corrections only are allowed (e.g., typos).
5) As part of the EMBO Publications transparent editorial process initiative (see our Editorial at http://embomolmed.embopress.org/content/2/9/329), EMBO Molecular Medicine will publish online a Review Process File (RPF) to accompany accepted manuscripts. This file will be published in conjunction with your paper and will include the anonymous referee reports, your point-by-point response and all pertinent correspondence relating to the manuscript. Let us know whether you agree with the publication of the RPF and as here, if you want to remove or not any figures from it prior to publication. Please note that the Authors checklist will be published at the end of the RPF.
6) Please provide a point-by-point letter INCLUDING my comments as well as the reviewer's reports and your detailed responses (as Word file).

I look forward to reading a new revised version of your manuscript as soon as possible.

Yours sincerely,

Zeljko Durdevic

Zeljko Durdevic
Senior Editor
EMBO Molecular Medicine

*** Instructions to submit your revised manuscript ***

*** PLEASE NOTE *** As part of the EMBO Publications transparent editorial process initiative (see our Editorial at https://www.embopress.org/doi/pdf/10.1002/emmm.201000094), EMBO Molecular Medicine will publish online a Review Process File to accompany accepted manuscripts.

In the event of acceptance, this file will be published in conjunction with your paper and will include the anonymous referee reports, your point-by-point response and all pertinent correspondence relating to the manuscript. If you do NOT want this file to be published, please inform the editorial office at contact@embomolmed.org.

*****

When preparing your revised manuscript, please refer to our guidelines: https://link.springer.com/journal/44321/submission-guidelines#cms-Revised-submissions. We perform an initial quality control of all revised manuscripts before re-review; failure to include requested items will delay the evaluation of your revision.

We require:

1) A .docx formatted version of the manuscript text (including legends for main figures, EV figures and tables). Please make sure that the changes are highlighted to be clearly visible.

2) Individual production quality figure files as .eps, .tif, .jpg (one file per figure). For guidance, download the 'Figure Guide PDF': https://media.springernature.com/original/springer-cms/rest/v1/content/27825798/data/v1.

3) A .docx formatted letter INCLUDING the reviewers' reports and your detailed point-by-point responses to their comments. As part of the EMBO Press transparent editorial process, the point-by-point response is part of the Review Process File (RPF), which will be published alongside your paper.

4) A complete author checklist, which you can download from our author guidelines. Please insert information in the checklist that is also reflected in the manuscript. The completed author checklist will also be part of the RPF.

5) Please note that all corresponding authors are required to supply an ORCID ID for their name upon submission of a revised manuscript.

6) It is mandatory to include a 'Data Availability' section after the Materials and Methods. Before submitting your revision, primary datasets produced in this study need to be deposited in an appropriate public database, and the accession numbers and database listed under 'Data Availability'. Please remember to provide a reviewer password if the datasets are not yet public.

In case you have no data that requires deposition in a public database, please state so in this section. Note that the Data Availability Section is restricted to new primary data that are part of this study.

7) For data quantification: please specify the name of the statistical test used to generate error bars and P values, the number (n) of independent experiments (specify technical or biological replicates) underlying each data point and the test used to calculate p-values in each figure legend. The figure legends should contain a basic description of n, P and the test applied. Graphs must include a description of the bars and the error bars (s.d., s.e.m.).

8) At EMBO Press we ask authors to provide source data for the main manuscript figures. You will receive a separate email with instructions for providing source data with your revised manuscript, including how to upload and organize the files.

9) Our journal encourages inclusion of *data citations in the reference list* to directly cite datasets that were re-used and obtained from public databases. Data citations in the article text are distinct from normal bibliographical citations and should directly link to the database records from which the data can be accessed. In the main text, data citations are formatted as

follows: "Data ref: Smith et al, 2001" or "Data ref: NCBI Sequence Read Archive PRJNA342805, 2017". In the Reference list, data citations must be labeled with "[DATASET]". A data reference must provide the database name, accession number/identifiers and a resolvable link to the landing page from which the data can be accessed at the end of the reference.

10) We replaced Supplementary Information with Expanded View (EV) Figures and Tables that are collapsible/expandable online. A maximum of 5 EV Figures can be typeset. EV Figures should be cited as 'Figure EV1, Figure EV2" etc... in the text and their respective legends should be included in the main text after the legends of regular figures.

- For the figures that you do NOT wish to display as Expanded View figures, they should be bundled together with their legends in a single PDF file called *Appendix*, which should start with a short Table of Content. Appendix figures should be referred to in the main text as: "Appendix Figure S1, Appendix Figure S2" etc.

- Additional Tables/Datasets should be labeled and referred to as Table EV1, Dataset EV1, etc. Legends have to be provided in a separate tab in case of .xls files. Alternatively, the legend can be supplied as a separate text file (README) and zipped together with the Table/Dataset file.

11) The paper explained: EMBO Molecular Medicine articles are accompanied by a summary of the articles to emphasize the major findings in the paper and their medical implications for the non-specialist reader. Please provide a draft summary of your article highlighting

- the medical issue you are addressing,

- the results obtained and

- their clinical impact.

This may be edited to ensure that readers understand the significance and context of the research. Please refer to any of our published articles for an example.

12) Author contributions: You will be asked to provide CRediT (Contributor Role Taxonomy) terms in the submission system. These replace a narrative author contribution section in the manuscript.

13) A Conflict of Interest statement should be provided in the main text.

14) Every published paper includes a 'Synopsis' to further enhance discoverability. Synopses are displayed on the journal webpage and are freely accessible to all readers. They include a short stand first (maximum of 300 characters, including space) as well as 2-5 one-sentences bullet points that summarizes the paper. Please write the bullet points to summarize the key NEW findings. They should be designed to be complementary to the abstract - i.e. not repeat the same text. We encourage inclusion of key acronyms and quantitative information (maximum of 30 words / bullet point). Please use the passive voice. Please attach these in a separate file or send them by email, we will incorporate them accordingly.

Please also suggest a striking image or visual abstract to illustrate your article as a PNG file 550 px wide x 300 px high.

15) Include a Reagents and Tools Table as part of the Methods section, which can be downloaded from our author guidelines.

EMBO Molecular Medicine has a "scooping protection" policy, whereby similar findings that are published by others during review or revision are not a criterion for rejection. Should you decide to submit a revised version, I do ask that you get in touch after three months if you have not completed it, to update us on the status.

*Additional important information regarding Figures

Each figure should be given in a separate file and should have the following resolution:
Graphs 800-1,200 DPI
Photos 400-800 DPI
Colour (only CMYK) 300-400 DPI"

Figures are not edited by the production team. All lettering should be the same size and style; figure panels should be indicated by capital letters (A, B, C etc). Gridlines are not allowed except for log plots. Figures should be numbered in the order of their appearance in the text with Arabic numerals. Each Figure must have a separate legend and a caption is needed for each panel.

*Additional important information regarding figures and illustrations can be found at
https://media.springernature.com/original/springer-cms/rest/v1/content/27825798/data/v1

***** Reviewer's comments *****

Referee #1 (Remarks for Author):

The revised manuscript addresses important points regarding nanoblade-induced editing in primary CD4+ T cells, as well as provides evidence of in vivo editing in ART-treated HIV-infected humanized mice. Moreover, the authors provide rationale for the low levels of Tat editing observed in vivo, which are relevant and add context to the data. This works represents a novel approach which will likely lay the foundation for future efforts improving the effectiveness and translational capacity of nanoblade-mediated excisions of HIV provirus to aid HIV cure efforts.

Referee #2 (Comments on Novelty/Model System for Author):

Although it is novel to develop the CD4-directed nanoblades for gene editing-based HIV suppression, such approach appeared to only demonstrate the modest anti-HIV effects at the most in vitro including the use of primary cells based on the current data. I appreciate that the authors presented the in vivo data using the humanized mice models of HIV, but such data have not been significantly improved despite that substantial time has been provided for revision. Unfortunately, the anti-HIV effects of CD4-directed nanoblades across the majority of data from the humanized mice experiments remained poor without any statistical significance. With the currently presented results, there is a doubt whether such approach could be further developed to treat HIV in clinic, leading to relatively low medical impact.

Referee #2 (Remarks for Author):

The authors have addressed some of my concerns. I also appreciate that the more primary cells have been included for evaluation during revision. However, some of my main concerns still remain. Although the study presented the novel application of CD4-directed nanoblades for gene editing-based HIV suppression, current experimental evidence only support that they might demonstrate some modest effects in vitro including the use of human primary cells. Unfortunately, even given the substantial revision time, the anti-HIV outcome of such approach in vivo using humanized mice models has not been significantly improved and still remained poor without any statistical significance. Thus, I have the concern whether it could be further developed as a promising anti-HIV approach to treat AIDS patients in future.

Referee #3 (Comments on Novelty/Model System for Author):

The new controls added by authors now confirm detectable genome editing in vivo and not only rely on changes in viral loads. These results have strengthened their conclusions. Authors have also repeated ex vivo experiments using additional donors and now have more robust statistical power to support their conclusions.

Referee #3 (Remarks for Author):

Authors have answered to all my comments. Notably, they performed ex vivo experiments in primary CD4 T cells with additional donors. They also quantified proviral genome editing in primary cells and ex vivo using Nanopore sequencing, thus providing proof that Cas9-mediated excision of a fragment of the tat coding sequence was indeed achieved.
These new results considerably strengthen the study, although the efficiency of genome editing in primary CD4 cell transduced ex vivo or in vivo remain very low and might not allow a significant clinical improvement in restricting HIV-1 replication and spreading in humans living with HIV-1. Nevertheless, the work represents an interesting proof of concept that could serve as a foundation for further developments in this area.
.

Dear editor and reviewers,

Thank you again for your thorough revisions and critical reflections to improve our manuscript. Please find our detailed answers to your suggestions and concerns below, written in blue. In the new manuscript version, adaptations to the original manuscript are visible in track changes.

Sincerely,
On behalf of all authors,
Prof. dr. Jolien Van Cleemput and Prof. dr. Linos Vandekerckhove

****

Editorial comments

1) We agree with shared referees' opinion that the study shows limited translational implication and that it rather represents a proof-of-concept. Please indicate in the abstract and in the manuscript where appropriate that this is a proof-of-concept study. Also, clearly state the limitation of the study regarding low *in vivo* efficiency and limited clinical implications. Please address referee #2 concerns by discussion. No additional experiments are required.

We agree with this comment and have explained the "proof-of-concept" nature of our study in lines 36, 358-360, 385-386.

Reviewer comments

Referee #1 (Remarks for Author):

The revised manuscript addresses important points regarding nanoblade-induced editing in primary CD4+ T cells, as well as provides evidence of *in vivo* editing in ART-treated HIV-infected humanized mice. Moreover, the authors provide rationale for the low levels of Tat editing observed *in vivo*, which are relevant and add context to the data. This works represents a novel approach which will likely lay the foundation for future efforts improving the effectiveness and translational capacity of nanoblade-mediated excisions of HIV provirus to aid HIV cure efforts.

We thank the reviewer for this positive feedback.

Referee #2 (Comments on Novelty/Model System for Author):

Although it is novel to develop the CD4-directed nanoblades for gene editing-based HIV suppression, such approach appeared to only demonstrate the modest anti-HIV effects at the most *in vitro* including the use of primary cells based on the current data. I appreciate that the authors presented the *in vivo* data using the humanized mice models of HIV, but such data have not been significantly improved despite that substantial time has been provided for revision. Unfortunately, the anti-HIV effects of CD4-directed nanoblades across the majority of data from the humanized mice experiments remained poor without any statistical significance. With the currently presented results, there is a doubt whether such approach could be further developed to treat HIV in clinic, leading to relatively low medical impact.

We agree that the effect of our CD4-directed Nanoblades is only modest is *in vivo*, but we did demonstrate a significant effect on HIV viremia in both experiments. With further optimization, CD4-NBs could be further developed into (part of) a potential HIV cure. Moreover, their *in vitro* and *in vivo* specificity for CD4+ cells and their potential to edit primary CD4+ T cells *in vitro* with little effect on cell viability (compared to electroporation) makes them also applicable in a broader medical context. This has been discussed in lines 380-382. Finally, we also made it more clear in the manuscript that it is a proof-of-concept study in lines 36, 358-360, 385-386.

Referee #2 (Remarks for Author):

The authors have addressed some of my concerns. I also appreciate that the more primary cells have been included for evaluation during revision. However, some of my main concerns still remain. Although the study presented the novel application of CD4-directed nanoblades for gene editing-based HIV suppression, current experimental evidence only support that they might demonstrate some modest effects *in vitro* including the use of human primary cells. Unfortunately, even given the substantial revision time, the anti-HIV outcome of such approach *in vivo* using humanized mice models has not been significantly improved and still remained poor without any statistical significance. Thus, I have the concern whether it could be further developed as a promising anti-HIV approach to treat AIDS patients in future.

See previous comment.

Referee #3 (Comments on Novelty/Model System for Author):

The new controls added by authors now confirm detectable genome editing *in vivo* and not only rely on changes in viral loads. These results have strengthened their conclusions. Authors have also repeated *ex vivo* experiments using additional donors and now have more robust statistical power to support their conclusions.

We thank the reviewer for this positive feedback.

Referee #3 (Remarks for Author):

Authors have answered to all my comments. Notably, they performed *ex vivo* experiments in primary CD4 T cells with additional donors. They also quantified proviral genome editing in primary cells and *ex vivo* using Nanopore sequencing, thus providing proof that Cas9-mediated excision of a fragment of the tat coding sequence was indeed achieved.

These new results considerably strengthen the study, although the efficiency of genome editing in primary CD4 cell transduced *ex vivo* or *in vivo* remain very low and might not allow a significant clinical improvement in restricting HIV-1 replication and spreading in humans living with HIV-1. Nevertheless, the work represents an interesting proof of concept that could serve as a foundation for further developments in this area.

We thank the reviewer for this positive feedback and agree with this comment. We have now pointed out more clearly that it is a proof-of-concept study in 36, 358-360, 385-386.

24th Jun 2026

Dear Prof. Van Cleemput,

We are pleased to inform you that your manuscript is accepted for publication and is now being sent to our publisher to be included in the next available issue of EMBO Molecular Medicine.

Your manuscript will be processed for publication by EMBO Press. It will be copy edited and you will receive page proofs prior to publication. Please note that you will be contacted by Springer Nature Author Services to complete licensing and payment information.

You may qualify for financial assistance for your publication charges - either via a Springer Nature fully open access agreement or an EMBO initiative. Check your eligibility: https://link.springer.com/journal/44321/how-to-publish-with-us

Should you be planning a Press Release on your article, please notify the production team via this form in order to coordinate publication and release dates: https://forms.cloud.microsoft/e/eH4A8JVP6g

If you have any questions, please do not hesitate to contact the Editorial Office. Thank you for your contribution to EMBO Molecular Medicine.

Yours sincerely,
Zeljko Durdevic

Zeljko Durdevic
Senior Editor
EMBO Molecular Medicine

>>> Please note that it is EMBO Molecular Medicine policy for the transcript of the editorial process (containing referee reports and your response letter) to be published as an online supplement to each paper. If you do NOT want this, you will need to inform the Editorial Office via email immediately. More information is available here: https://link.springer.com/partners/embo-press/editorial-policies#Peer%20review

EMBO Press Author Checklist

Corresponding Author Name: Jolien Van Cleemput	
Journal Submitted to: EMBO Molecular Medicine	
Manuscript Number: EMM-2025-22529	

USEFUL LINKS FOR COMPLETING THIS FORM
The EMBO Journal - Author Guidelines
EMBO Reports - Author Guidelines
Molecular Systems Biology - Author Guidelines
EMBO Molecular Medicine - Author Guidelines

Reporting Checklist for Life Science Articles (updated January
This checklist is adapted from Materials Design Analysis Reporting (MDAR) Checklist for Authors. MDAR establishes a minimum set of requirements in transparent reporting in the life sciences (see Statement of Task: 10.31222/osf.io/9sm4x). Please follow the journal's guidelines in preparing your
Please note that a copy of this checklist will be published alongside your article.

Abridged guidelines for figures
1. Data
The data shown in figures should satisfy the following conditions:
- the data were obtained and processed according to the field's best practice and are presented to reflect the results of the experiments in an accurate and unbiased manner.
- ideally, figure panels should include only measurements that are directly comparable to each other and obtained with the same assay.
- plots include clearly labeled error bars for independent experiments and sample sizes. Unless justified, error bars should not be shown for technical
- if n<5, the individual data points from each experiment should be plotted. Any statistical test employed should be justified.
- Source Data should be included to report the data underlying figures according to the guidelines set out in the authorship guidelines on Data

2. Captions
Each figure caption should contain the following information, for each panel where they are relevant:
- a specification of the experimental system investigated (eg cell line, species name).
- the assay(s) and method(s) used to carry out the reported observations and measurements.
- an explicit mention of the biological and chemical entity(ies) that are being measured.
- an explicit mention of the biological and chemical entity(ies) that are altered/varied/perturbed in a controlled manner.
- the exact sample size (n) for each experimental group/condition, given as a number, not a range;
- a description of the sample collection allowing the reader to understand whether the samples represent technical or biological replicates (including how many animals, litters, cultures, etc.).
- a statement of how many times the experiment shown was independently replicated in the laboratory.
- definitions of statistical methods and measures:
  - common tests, such as t-test (please specify whether paired vs. unpaired), simple χ2 tests, Wilcoxon and Mann-Whitney tests, can be unambiguously identified by name only, but more complex techniques should be described in the methods section;
  - are tests one-sided or two-sided?
  - are there adjustments for multiple comparisons?
  - exact statistical test results, e.g., P values = x but not P values < x;
  - definition of 'center values' as median or average;
  - definition of error bars as s.d. or s.e.m.

Please complete ALL of the questions below.
Select "Not Applicable" only when the requested information is not relevant for your study.

Materials

Newly Created Materials	Information included in the manuscript?	In which section is the information available? (Reagents and Tools Table, Materials and Methods, Figures, Data Availability Section)
New materials and reagents need to be available; do any restrictions apply?	Yes	Reagents and Tools Table, Materials and Methods

Antibodies	Information included in the manuscript?	In which section is the information available? (Reagents and Tools Table, Materials and Methods, Figures, Data Availability Section)
For **antibodies** provide the following information:<br>- Commercial antibodies: RRID (if possible) or supplier name, catalogue number and or/clone number<br>- Non-commercial: RRID or citation	Yes	Reagents and Tools Table, Materials and Methods

DNA and RNA sequences	Information included in the manuscript?	In which section is the information available? (Reagents and Tools Table, Materials and Methods, Figures, Data Availability Section)
**Short novel DNA or RNA including primers, probes**: provide the sequences.	Yes	Reagents and Tools Table, Materials and Methods

Cell materials	Information included in the manuscript?	In which section is the information available? (Reagents and Tools Table, Materials and Methods, Figures, Data Availability Section)
**Cell lines:** Provide species information, strain. Provide accession number in repository **OR** supplier name, catalog number, clone number, and/**OR** RRID.	Yes	Reagents and Tools Table, Materials and Methods
**Primary cultures:** Provide species, strain, sex of origin, genetic modification status.	Yes	Reagents and Tools Table, Materials and Methods
Report if the cell lines were recently **authenticated** (e.g., by STR profiling) and tested for mycoplasma contamination.	Yes	Materials and Methods

Experimental animals	Information included in the manuscript?	In which section is the information available? (Reagents and Tools Table, Materials and Methods, Figures, Data Availability Section)
**Laboratory animals or Model organisms:** Provide species, strain, sex, age, genetic modification status. Provide accession number in repository **OR** supplier name, catalog number, clone number, **OR** RRID.	Yes	Reagents and Tools Table, Materials and Methods, Results, Figure 6
**Animal observed in or captured from the field:** Provide species, sex, and age where possible.	Not Applicable	
Please detail **housing and husbandry conditions**.	Yes	Materials and Methods

Plants and microbes	Information included in the manuscript?	In which section is the information available? (Reagents and Tools Table, Materials and Methods, Figures, Data Availability Section)
**Plants:** provide species and strain, ecotype and cultivar where relevant, unique accession number if available, and source (including location for collected wild specimens).	Not Applicable	
**Microbes:** provide species and strain, unique accession number if available, and source.	Yes	Reagents and Tools Table

Human research participants	Information included in the manuscript?	In which section is the information available? (Reagents and Tools Table, Materials and Methods, Figures, Data Availability Section)
If collected and within the bounds of privacy constraints report on age, sex and gender or ethnicity for all study participants.	Not Applicable	

Core facilities	Information included in the manuscript?	In which section is the information available? (Reagents and Tools Table, Materials and Methods, Figures, Data Availability Section)
If your work benefited from core facilities, was their service mentioned in the acknowledgments section?	Yes	

Design

Study protocol	Information included in the manuscript?	In which section is the information available? (Reagents and Tools Table, Materials and Methods, Figures, Data Availability Section)
If study protocol has been **pre-registered, provide DOI in the manuscript**. For clinical trials, provide the trial registration number **OR** cite DOI.	Not Applicable	
Report the **clinical trial registration number** (at ClinicalTrials.gov or equivalent), where applicable.	Not Applicable	

Laboratory protocol	Information included in the manuscript?	In which section is the information available? (Reagents and Tools Table, Materials and Methods, Figures, Data Availability Section)
Provide DOI OR other citation details if **external detailed step-by-step protocols** are available.	Not Applicable	

Experimental study design and statistics	Information included in the manuscript?	In which section is the information available? (Reagents and Tools Table, Materials and Methods, Figures, Data Availability Section)
Include a statement about **sample size** estimate even if no statistical methods were used.	Yes	Results and Figure Legends
Were any steps taken to minimize the effects of subjective bias when allocating animals/samples to treatment (e.g. **randomization procedure**)? If yes, have they been described?	Yes	Materials and Methods
Include a statement about **blinding** even if no blinding was done.	Yes	Materials and Methods
Describe **inclusion/exclusion criteria** if samples or animals were excluded from the analysis. Were the criteria pre-established?<br><br>If sample or data points were omitted from analysis, report if this was due to attrition or intentional exclusion and provide justification.	Yes	Materials and Methods
For every figure, are **statistical tests** justified as appropriate? Do the data meet the assumptions of the tests (e.g., normal distribution)? Describe any methods used to assess it. Is there an estimate of variation within each group of data? Is the variance similar between the groups that are being statistically compared?	Yes	Materials and Methods, Figure Legends

Sample definition and in-laboratory replication	Information included in the manuscript?	In which section is the information available? (Reagents and Tools Table, Materials and Methods, Figures, Data Availability Section)
In the figure legends: state number of times the experiment was **replicated** in laboratory.	Yes	Figure Legends
In the figure legends: define whether data describe **technical or biological replicates**.	Yes	Figure Legends

Ethics

Ethics	Information included in the manuscript?	In which section is the information available? (Reagents and Tools Table, Materials and Methods, Figures, Data Availability Section)
Studies involving **human participants**: State details of **authority granting ethics approval** (IRB or equivalent committee(s), provide reference number for approval.	Not Applicable	
Studies involving **human participants**: Include a statement confirming that **informed consent** was obtained from all subjects and that the experiments conformed to the principles set out in the WMA Declaration of Helsinki and the Department of Health and Human Services Belmont Report.	Not Applicable	
Studies involving **human participants: For** publication of **patient photos**, include a statement confirming that consent to publish was obtained.	Not Applicable	
Studies involving experimental **animals**: State details of **authority granting ethics approval** (IRB or equivalent committee(s), provide reference number for approval. Include a statement of compliance with ethical regulations.	Yes	Materials and Methods
Studies involving **specimen and field samples:** State if relevant **permits** obtained, provide details of authority approving study; if none were required, explain why.	Not Applicable	

Dual Use Research of Concern (DURC)	Information included in the manuscript?	In which section is the information available? (Reagents and Tools Table, Materials and Methods, Figures, Data Availability Section)
Could your study fall under dual use research restrictions? Please check biosecurity documents and list of **select agents and toxins** (CDC): https://www.selectagents.gov/sat/list.htm	Not Applicable	
If you used a select agent, is the security level of the lab appropriate and reported in the manuscript?	Not Applicable	
If a study is subject to dual use research of concern regulations, is the name of the **authority granting approval and reference number** for the regulatory approval provided in the manuscript?	Not Applicable	

Reporting

The MDAR framework recommends adoption of discipline-specific guidelines, established and endorsed through community initiatives. Journals have their own policy about requiring specific guidelines and recommendations to complement MDAR.

Adherence to community standards	Information included in the manuscript?	In which section is the information available? (Reagents and Tools Table, Materials and Methods, Figures, Data Availability Section)
State if relevant guidelines or checklists (e.g., **ICMJE, MIBBI, ARRIVE, PRISMA**) have been followed or provided.	Not Applicable	
For **tumor marker prognostic studies,** we recommend that you follow the **REMARK** reporting guidelines (see link list at top right). See author guidelines, under 'Reporting Guidelines'. Please confirm you have followed these guidelines.	Not Applicable	
For **phase II and III randomized controlled trials**, please refer to the **CONSORT** flow diagram (see link list at top right) and submit the CONSORT checklist (see link list at top right) with your submission. See author guidelines, under 'Reporting Guidelines'. Please confirm you have submitted this list.	Not Applicable	

Data Availability

Data availability	Information included in the manuscript?	In which section is the information available? (Reagents and Tools Table, Materials and Methods, Figures, Data Availability Section)
Have **primary datasets** been deposited according to the journal's guidelines (see 'Data Deposition' section) and the respective accession numbers provided in the Data Availability Section?	Not Applicable	
Were **human clinical and genomic datasets** deposited in a public access-controlled repository in accordance to ethical obligations to the patients and to the applicable consent agreement?	Not Applicable	
Are **computational models** that are central and integral to a study available without restrictions in a machine-readable form? Were the relevant accession numbers or links provided?	Not Applicable	
If publicly available data were reused, provide the respective **data citations in the reference list**.	Not Applicable	
