# Supplementary material for: CD4-directed nanoblades enable selective genome editing in CD4+ cells and HIV suppression in vitro and in vivo
Source: EMBO Mol Med. 2026 Jul 6;18(8):3345–73. doi: 10.1038/s44321-026-00481-x (PMC13470122; doi:10.1038/s44321-026-00481-x)
Supplement: Supplementary file 3 [file 44321_2026_481_MOESM9_ESM.pdf]

Expanded View Figures

Figure EV1. Sequential gating to identify specific human PBMC subsets incubated with CD4-directed kirsch nanoblades (CD4 K-NBs).

The gating strategy of a representative sample (0 ng mCherry donor 1) is shown by arrows.

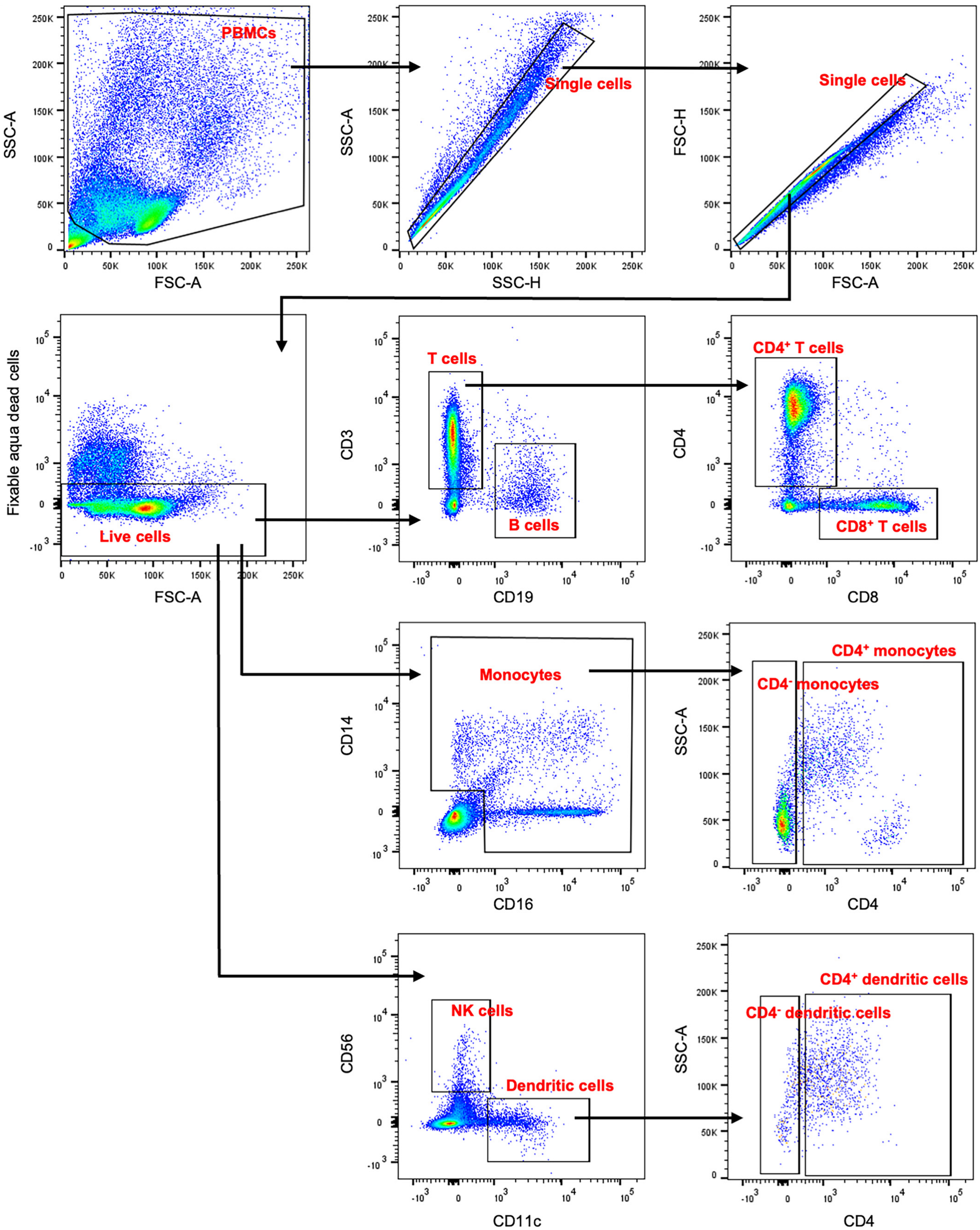
PBMCs
SSC-A
FSC-A
Single cells
SSC-A
SSC-H
Single cells
FSC-H
FSC-A
Fixable aqua dead cells
Live cells
FSC-A
T cells
B cells
CD3
CD19
CD4⁺ T cells
CD8⁺ T cells
CD4
CD8
Monocytes
CD14
CD16
CD4⁺ monocytes
CD4⁻ monocytes
SSC-A
CD4
NK cells
Dendritic cells
CD56
CD11c
CD4⁺ dendritic cells
CD4⁻ dendritic cells
SSC-A
CD4

© The Author(s)

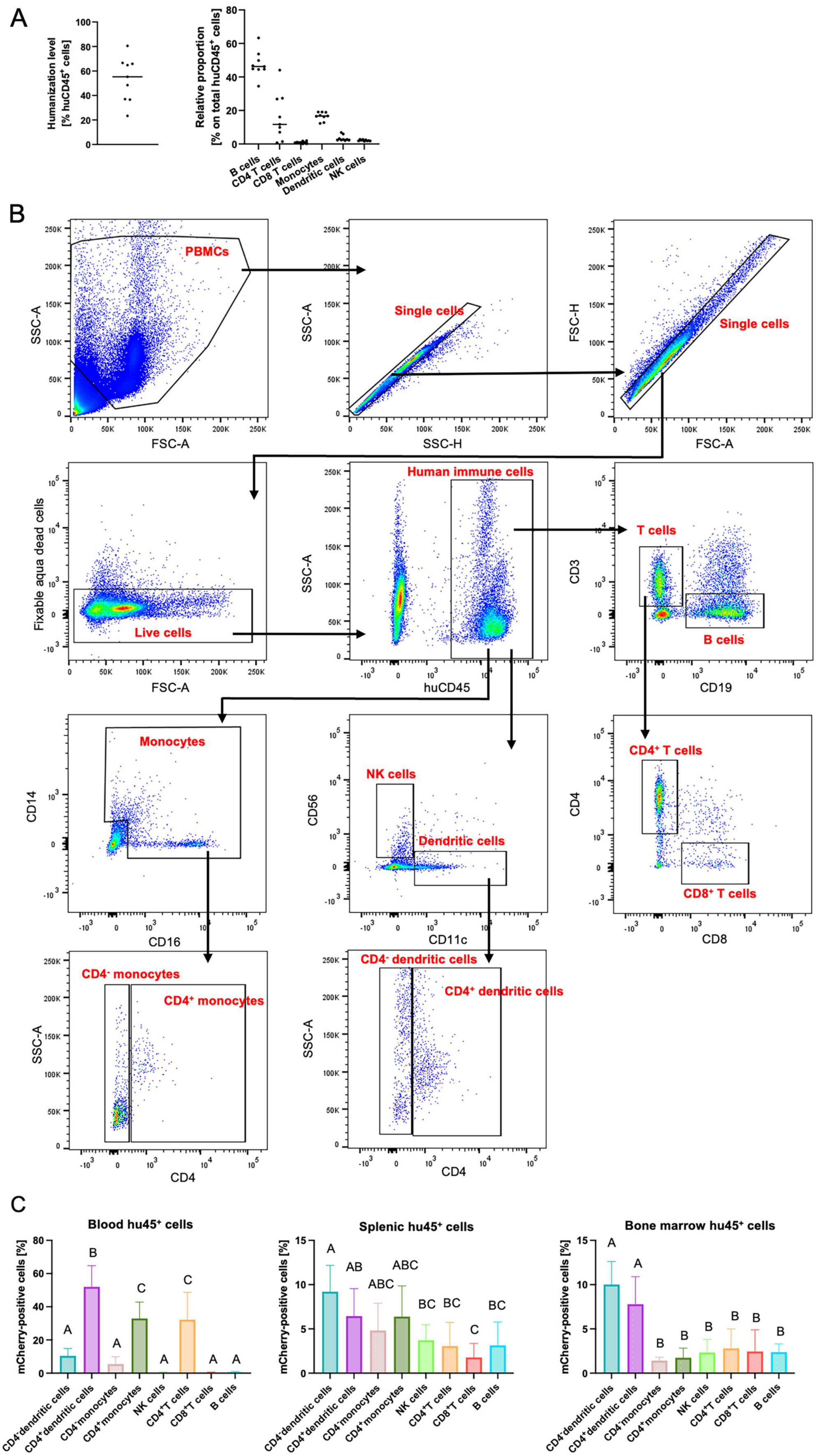
A
Humanization level [% huCD45+ cells]
Relative proportion [% on total huCD45+ cells]
B cells
CD4 T cells
CD8 T cells
Monocytes
Dendritic cells
NK cells
B
PBMCs
Single cells
Single cells
Live cells
Human immune cells
T cells
B cells
Monocytes
NK cells
Dendritic cells
CD4+ T cells
CD8+ T cells
CD4- monocytes
CD4+ monocytes
CD4- dendritic cells
CD4+ dendritic cells
SSC-A
FSC-A
SSC-H
FSC-H
Fixable aqua dead cells
huCD45
CD3
CD19
CD14
CD16
CD56
CD11c
CD4
CD8
C
Blood hu45+ cells
Splenic hu45+ cells
Bone marrow hu45+ cells
mCherry-positive cells [%]
CD4-dendritic cells
CD4+dendritic cells
CD4-monocytes
CD4+monocytes
NK cells
CD4+T cells
CD8+T cells
B cells

◀ **Figure EV2. Characterization of humanized mice and gating strategy to determine in vivo binding of CD4 K-NBs to human immune cells.**

(**A**) Left panel: dot blot with grand mean of humanization levels of mice ($n = 9$) prior to injection with CD4 K-NBs shown as % of $huCD45^+$ cells over total $muCD45^+$ and $huCD45^+$ cells. Right panel: Relative proportion of specific human immune cell subsets in these humanized mice represented as % of specific cell types over total $huCD45^+$ cells. (**B**) Sequential gating to identify specific human immune cell subsets in humanized mice injected with CD4 K-NBs. The gating strategy of a representative sample (0 ng mCherry) is shown by arrows. (**C**) Binding of CD4 K-NBs to $huCD45^+$ immune cell subsets, expressed as % of mCherry-positive cells in blood, spleen, and bone marrow of humanized mice 3 h post injection. Analysis was done with one-way ANOVA followed by a Tukey post hoc test (mean + SD, different letters indicate significant [$p < 0.05$] differences, $n = 7$ mice for blood and 5 mice for spleen and bone marrow).

 © The Author(s)

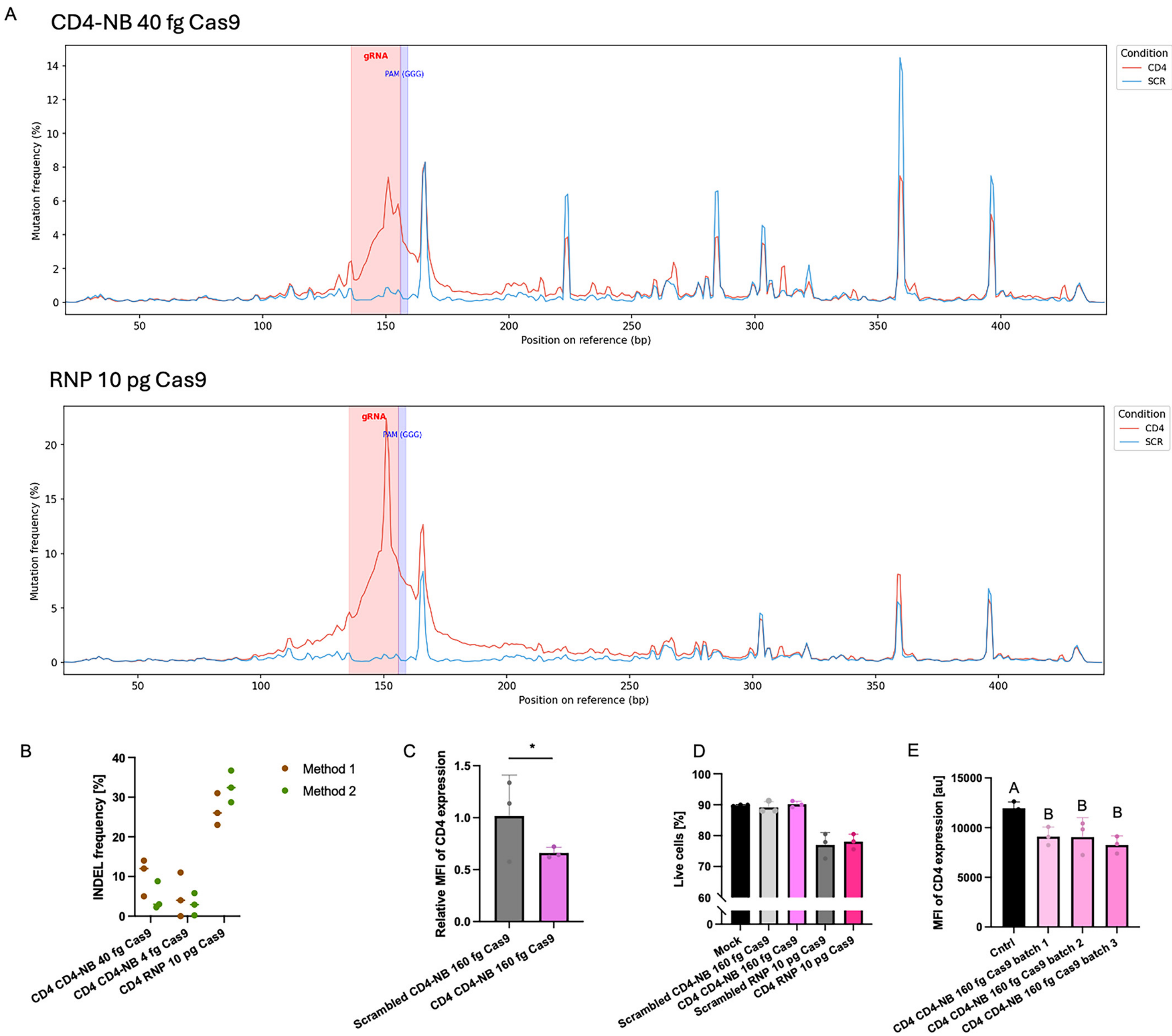

Figure EV3. Additional analyses of CD4 gene editing by CD4-NBs in primary human CD4⁺ cells.

(**A**) Mutation frequencies of *CD4* following treatment with CD4-NBs (upper panel) or sgRNA-Cas9 RNPs (lower panel) in primary CD4⁺ cells. The scrambled and CD4 gRNA conditions are depicted in blue and red, respectively. The gRNA and PAM sequences are shaded in red and blue on the graphs, respectively. (**B**) Bar chart representing INDEL detection frequency at the *CD4* locus following CD4-NB treatment (40 or 4 fg Cas9/cell) or sgRNA-Cas9 electroporation (10 pg Cas9/cell) using two different methods on PCR amplicons. Method 1: Sanger sequencing followed by tracking INDELs via Synthego Inference of CRISPR Edits analysis (Nakamae and Bono, 2022); method 2: Oxford Nanopore Technologies (ONT) sequencing to determine mutation frequencies 10 bp around the gRNA cut site of *CD4* (PathoSense, Gent, Belgium). Analysis was done using a two-way ANOVA ($F_{1,12} = 0.086$, $p = 0.774$, $n = 3$ biological replicates). (**C**) Bar chart representing CD4 knockdown, expressed as CD4 MFI, in primary CD4⁺ cells following a high dose of scrambled or CD4 CD4-NB transduction. Analysis was done using a one-way ANOVA ($n = 3$ technical replicates of three biological replicates, $^*p = 0.05$). (**D**) Bar chart showing cell viability following different doses of CD4-NB transduction or sgRNA-Cas9 RNP electroporation ($n = 3$ technical replicates of 3 biological replicates). (**E**) Bar chart representing CD4 knockdown, expressed as CD4 MFI, in primary CD4⁺ cells following transduction with three different batches of CD4 CD4-NBs on one donor. Analysis was done with one-way ANOVA followed by a Tukey post hoc test ($n = 3$ technical replicates). Significant letters are indicated by different letters ($p < 0.05$, exact p values are reported in Appendix Table S4).

© The Author(s)

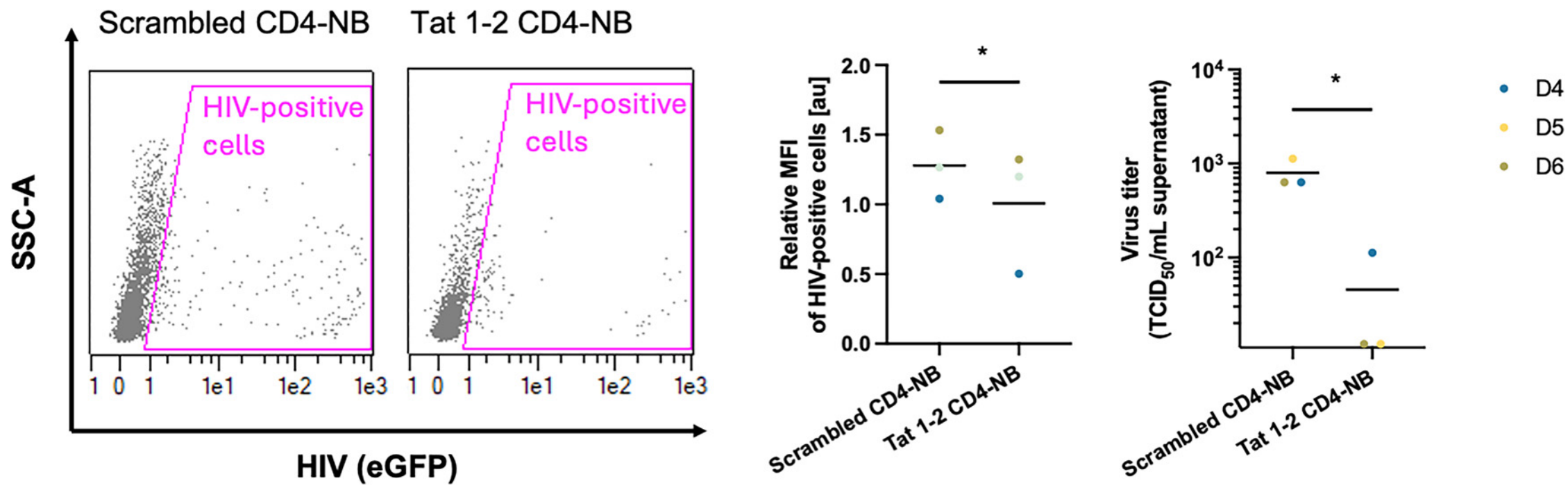

Figure EV4. CD4-NB treatment of three additional donors following HIV infection at lower MOI (0.01).

Left panel: Representative scatter dot plots analyzed by flow cytometry for HIV gene (*eGFP*) expression of live primary CD4⁺ cells treated with scrambled or tat 1-2 CD4-NBs. Middle panel: dot blot with grand median representing average eGFP MFI of HIV-infected primary CD4⁺ cells treated with different CD4-NBs at a dose of 40 fg Cas9/cell. Analysis was done using one-way ANOVA (three technical replicates on three biological replicates, $^*p = 0.042$). Right panel: dot blot with grand mean representing virus titer, expressed as $TCID_{50}$/mL, in supernatant of $HIV_{NLENG1\text{-}IRES}$-infected primary CD4⁺ cells treated with different CD4-NBs at a dose of 40 fg Cas9/cell. Analysis was done using an independent samples *t*-test (three biological replicates, $^*p = 0.029$).

© The Author(s)

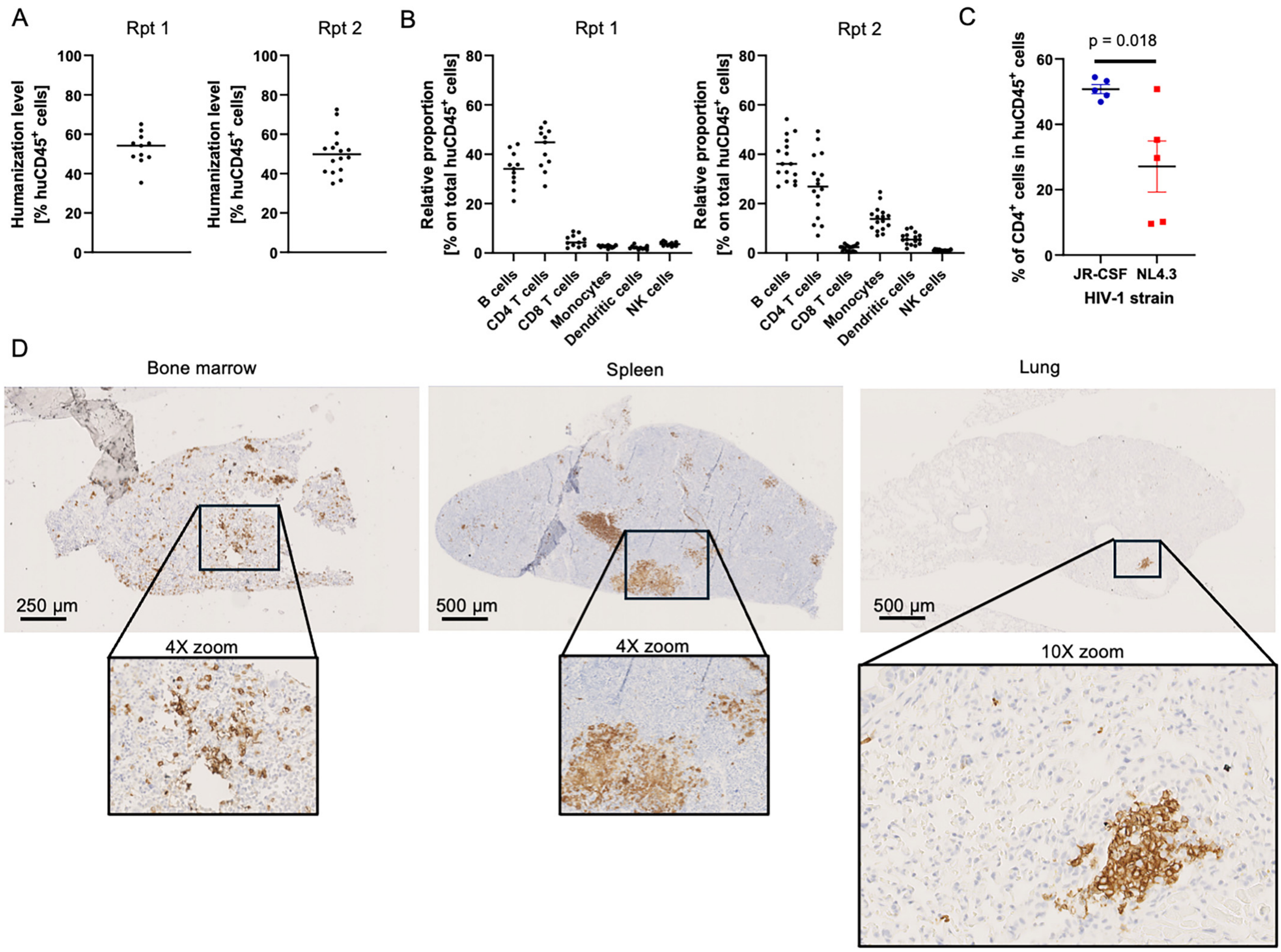

Figure EV5. Characterization of humanized mice used for HIV infection experiments.

(**A**) Humanization levels of mice prior to $HIV_{JR\text{-}CSF}$ infection shown as % of $huCD45^+$ cells over total $muCD45^+$ and $huCD45^+$ cells ($n = 11$ and 16 for repeat [rpt] 1 and 2, respectively). (**B**) Relative proportion of specific human immune cell subsets in these humanized mice represented as % of specific cell types over total $huCD45^+$ cells ($n = 11$ and 16 for rpt 1 and 2, respectively). (**C**) CD4 cell levels in humanized mice upon infection with $HIV_{JR\text{-}CSF}$ or $HIV_{NL4.3}$. Analysis was done using an independent samples *t*-test ($n = 5$ biological replicates). The scatter plot shows the mean ± SEM. (**D**) Expression of human HLA-DR in the spleen and bone marrow of a representative humanized mouse (scrambled CD4-NB) confirms human cell reconstitution in different tissues at the end of the experiment.

© The Author(s)
